# A large therian mammal from the Late Cretaceous of South America

**DOI:** 10.1038/s41598-024-53156-3

**Published:** 2024-02-03

**Authors:** Nicolás R. Chimento, Federico L. Agnolín, Jordi García-Marsà, Makoto Manabe, Takanobu Tsuihiji, Fernando E. Novas

**Affiliations:** 1 Laboratorio de Anatomía Comparada y Evolución de los Vertebrados (LACEV), Museo Argentino de Ciencias Naturales “Bernardino Rivadavia” (MACN-CONICET), Av. Ángel Gallardo 470, C1405DJR Ciudad Autónoma de Buenos Aires, Argentina; 2 Fundación de Historia Natural “Félix de Azara”, Departamento de Ciencias Naturales y Antropología, CEBBAD - Universidad Maimónides, Hidalgo 767, C1405BDB Buenos Aires, Argentina; 3 Consejo Nacional de Investigaciones Científicas y Técnicas (CONICET), Buenos Aires, Argentina; 4 National Museum of Nature and Science, 4‑1‑1 Amakubo, Tsukuba, 305‑0005 Japan; 5 Department of Earth and Planetary Science, The University of Tokyo, 7‑3‑1 Hongo, Bunkyo-ku, Tokyo, 305‑0005 Japan

**Keywords:** Palaeontology, Palaeontology

## Abstract

Theria represent an extant clade that comprises placental and marsupial mammals. Here we report on the discovery of a new Late Cretaceous mammal from southern Patagonia, *Patagomaia chainko* gen. et sp. nov., represented by hindlimb and pelvic elements with unambiguous therian features. We estimate *Patagomaia chainko* attained a body mass of 14 kg, which is considerably greater than the 5 kg maximum body mass of coeval Laurasian therians. This new discovery demonstrates that Gondwanan therian mammals acquired large body size by the Late Cretaceous, preceding their Laurasian relatives, which remained small-bodied until the beginning of the Cenozoic. *Patagomaia* supports the view that the Southern Hemisphere was a cradle for the evolution of modern mammalian clades, alongside non-therian extinct groups such as meridiolestidans, gondwanatherians and monotremes.

## Introduction

Mesozoic mammals are usually regarded as small-sized, insectivore, shrew-like creatures of nocturnal habits^1–4^. This traditional view has recently changed thanks to discoveries that notably increased the ecological and morphological disparity of Mesozoic mammals, including swimming, burrowing and gliding taxa^5–10^, as well as dog-sized forms^11–13^. Among them, the fossil record of Late Cretaceous therian mammals from the Laurasian continents includes a wide array of small-bodied shrew-like insectivores of therian affinities^1,2^. Because the fossil record of modern-line mammals is notably rich in the Northern Hemisphere, it has been usually argued that the early evolution and origin of therians occurred exclusively on Laurasian landmasses, with therian mammals being rare—if not entirely absent—in Gondwana^14–18^. However, some molecular analyses^19–26^ in conjunction with sparce paleontological data^27–35^ are lending support to the view that some therian lineages, at least, evolved and diversified in the Southern Hemisphere in Late Mesozoic times.

Here we report on a therian mammal from the Mesozoic of Patagonia. Present discovery is of high relevance, because it supports the idea that southern landmasses constituted an important theatre for the early evolution of modern mammals. It is also indicative of the persistence of wide gaps in the fossil record of the Southern Hemisphere, up to now representing about 5% of the global fossil record of Mesozoic mammals^33^.

Class Mammalia Linnaeus 1758

Subclass Theria Parker and Haswell, 1897

***Patagomaia*** nov. gen.

### Generic diagnosis

*Patagomaia* is a large mammal (~ 14 kg) distinguished by the following unique combination of character states: fused acetabulum with a complete rim lacking a dorsal emargination; femur with subspherical head having a well-defined fovea capitis; femur head separated from the rest of the bone by a well-defined and medially tilted neck; lesser trochanter of femur small and located on the posteromedial surface of the shaft; distal end of femur with nearly symmetrical distal condyles and reduced epicondyles. *Patagomaia* further differs from other Mesozoic mammals in having the autapomorphic condition of a thick, well-defined, and obliquely oriented intercondylar ridge delimiting a deep fossa at the distal end of the femur.

### Type species

*Patagomaia chainko* sp. nov.

### Etymology

*Patago*, from Patagonia; *maia*, mother in Greek.

***Patagomaia chainko*** sp. nov.

Zoobank registration: urn:lsid:zoobank.org:pub:39766F43-6876-4460-9830-D80646C312CC

### Holotype

**Figure 1:**
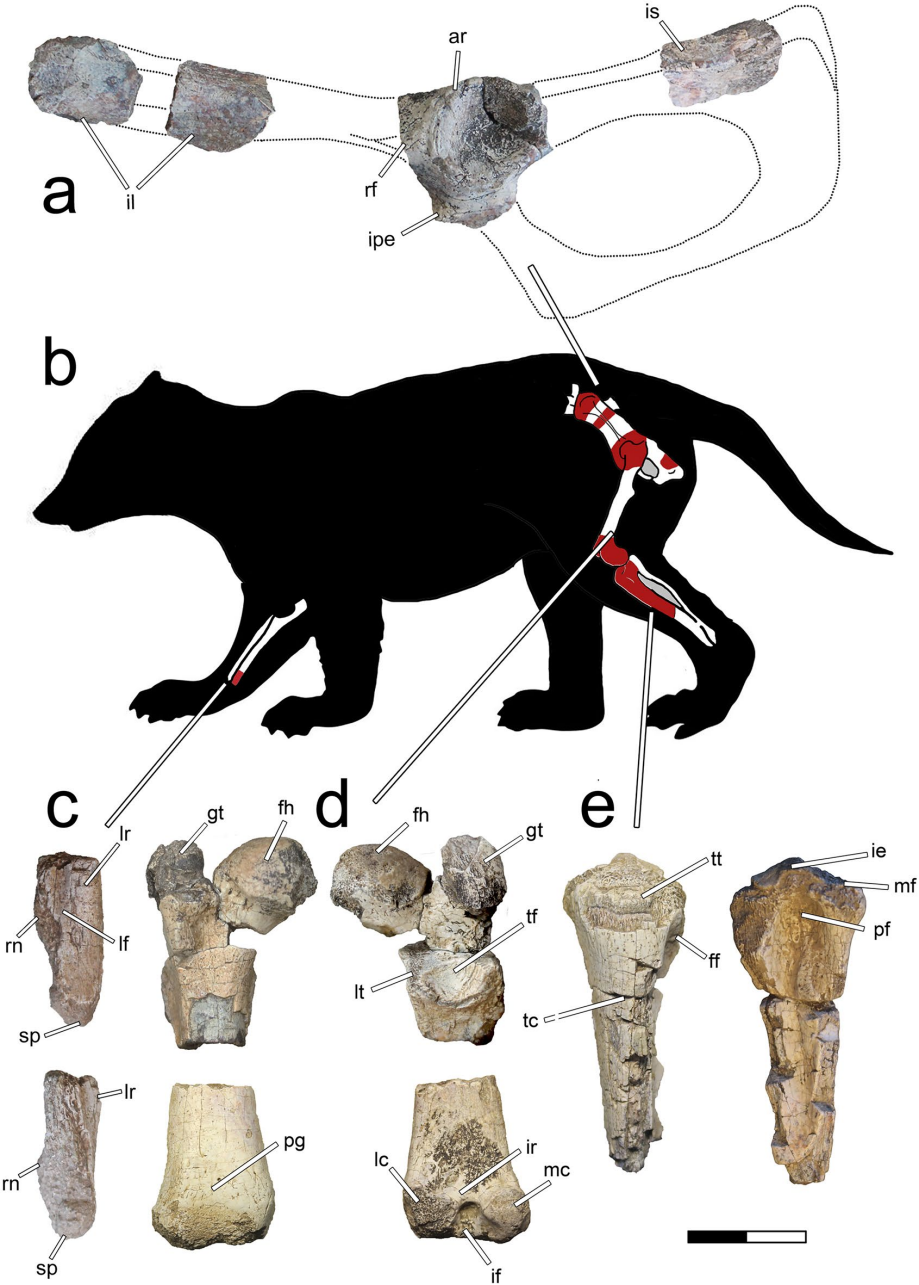
Images of *Patagomaia chainko* holotype remains, MPM-PV-23365 References: (**a**) fragments of the left pelvis; (**b**) silhouette and skeletal scheme with details of the preserved bones; (**c**) distal end of the left ulna; (**d**) proximal end of the right femur and distal end of the left femur; e, proximal half of the left tibia. Scale bar: 20 mm.

MPM-PV-23365 (Museo Padre Molina, Río Gallegos, Santa Cruz, Argentina), associated postcranial remains including the distal end of the left ulna, two fragments of the preacetabular wing of the left ilium, acetabular region of the left hemipelvis, fragment of the ischial blade, proximal end of the right femur; distal end of the left femur, proximal end of the left tibia, and other indeterminate bone fragments (Fig. 1; Supplementary Figs. 1, 3, 4, 6, 7).

### Referred material

MPM-PV-23366, partial left acetabulum and ischium (see Supplementary Fig. 2); incomplete right femoral shaft (see Supplementary Fig. 5).

### Etymology

The species name is derived from the Aonikenk language: *chaink*, large and *ko*, bone.

### Diagnosis

The same as for genus by monotypy.

### Type locality and age

La Anita Farm, Santa Cruz, Argentina. The remains were collected at a new site (S 50° 30′ 39.888″, W 72° 33′ 18.035″) geographically close to and at the same stratigraphic level as the *Isasicursor* 2 site (Chorrillo Formation, lower Maastrichtian, Upper Cretaceous)^36,37^. Both the holotype and referred specimens were found within an area of about 20 × 30 m. In close proximity, fossil wood and indeterminate hadrosaurid remains were found.

## Description

The preacetabular wing of the ilium has a well-defined acetabular ridge that separates the dorsal gluteal fossa from the ventral iliac fossa, as in other early diverging therians^38–40^. There is a thickened surface for the origin of the *m*. *rectus femoris* anterior to the acetabulum, similar to some placental mammals but in contrast to other therians^41^, non-therian mammals and some early diverging therians (e.g., multituberculates *Akidolestes, Henkelotherium, Vincelestes, Ukhaatherium*)^41–43^ in which there is a distinct preacetabular tubercle (see Supplementary Information 1; Supplementary Fig. 8).

The pubis, ischium, and ilium of *Patagomaia* are strongly fused in the acetabular region, in sharp contrast with the unfused condition in many non-therian mammaliaforms (e.g., morganucodontids, dryolestoids, docodontans, multituberculates, eutriconodonts)^7,11,42–47^.

In *Patagomaia,* the acetabulum is deep and subspherical, and it is surrounded by a complete rim lacking the dorsal emargination that characterizes non-therian mammaliaforms (e.g., docodontans, multituberculates, *Vincelestes*; see Supplementary Figs. 1, 2, 11, 16)^7,45^. The acetabular rim shows a prominent anterior wall, corresponding with a subspherical femoral head. The lunate surface of the acetabulum is wide, with a deep acetabular fossa posteroventrally continuous with a narrow acetabular notch. Although the iliopubic eminence is represented only by its base, it appears to have been robust, as in monotremes and some therians^42^, but different from many other Mesozoic taxa (e.g., *Adalatherium, Vincelestes*; see Supplementary Fig. 11).

The femoral head of *Patagomaia* is clearly offset from the rest of the bone, unlike non-therian mammals in which it is close to the main axis of the femur (e.g., monotremes, *Akidolestes*, *Vincelestes*)^42^. The femoral neck is constricted and medially oriented, forming an angle of about 55° with the main axis of femur, whereas non-therian mammals lack a constricted femoral neck (e.g., monotremes, *Akidolestes*, *Vincelestes*)^42^. The trochanteric fossa is well-defined, wide, and distally bounded by a crescent-shaped crest for the insertion of the *m. quadratus femoris*^39,40^, in contrast to the shallow poorly defined fossa in non-therian mammals (e.g., *Akidolestes, Henkelotherium, Vincelestes*)^42,43^. The greater trochanter is robust, anteroposteriorly broad, and projected posteriorly; it reaches the same level as the femoral head proximally, resembling many therians^39,48,49^ but unlike non-therian mammals (e.g., monotremes, multituberculates, *Henkelotherium, Vincelestes*)^42–45,50,51^. The lesser trochanter of *Patagomaia* is relatively small and posteroventrally displaced. A thick crest extends posterodistally from the base of the greater trochanter. Such a crest is usually associated with the presence of a prominent third trochanter, suggesting that this trochanter was also developed in *Patagomaia* but is broken off and missing from the holotype (Supplementary Figs. 3 and 11).

The proximal end of the femur of *Patagomaia* lacks diagnostic features of multituberculates, such as the presence of a post-trochanteric fossa, a prominent greater trochanter that extends proximally beyond the femoral head and is separated from it by a deep incisure, a plate-like lesser trochanter at the confluence of the greater trochanter and neck that strongly protrudes ventrally, or the presence of a subtrochanteric tubercle^45,50,51^.

The posterior position of the lesser trochanter gives the femur of *Patagomaia* a subquadrangular contour in proximal view, as in therian mammals (e.g., *Argyrolagus*, *Ukhaatherium,* leptictids*, Meniscotherium, Microgale*)^41,49,52^. This condition differs from most non-therian mammaliaforms (e.g., morganucodontans, docodontans, *Henkelotherium*, *Vincelestes, Necrolestes*)^43,53,54^ in which the greater trochanter, femoral head, and lesser trochanter are almost aligned (see Supplementary Fig. 9).

The distal end of the femur of *Patagomaia* is transversely narrow, with the distal condyles proximo-distally low and nearly symmetrical in size and shape, in contrast with most non-therian mammals (e.g., morganucodontans, monotremes, *Haldanodon*; *Docofossor, Necrolestes*)^44,53–55^ in which the distal condyles are markedly asymmetrical (see Supplementary Fig. 10). The femoral condyles of *Patagomaia* are separated posteriorly by a deep but narrow intercondylar groove. There is a thick and prominent intercondylar ridge that is obliquely oriented and is distally delimited by a pit-like concavity (see Supplementary Fig. 4). This condition is unknown in most other mammals, being observed in only a few extant therians (e.g., *Arctictis, Hystrix, Meles*; see Supplementary Fig. 12). In *Patagomaia,* the patellar surface is represented by a shallow concavity delimited by low crests.

The tibia of *Patagomaia* shows symmetrical proximal facets for articulation with the femoral condyles, in contrast to the asymmetrical articular facets of non-therian mammaliaforms (e.g., morganucodontans, docodontans, *Akidolestes*, *Vincelestes*; see Supplementary Figs. 6)^7,42,54^. A deep and well-defined fossa extends along the posterior surface of the shaft of the tibia, a trait only seen in some eutherians (e.g., leptictids^56^, periptychids^57^, carnivores; see Supplementary Fig. 13). The anterior tibial tubercle is low and extends distally as a crest.

**Figure 2:**
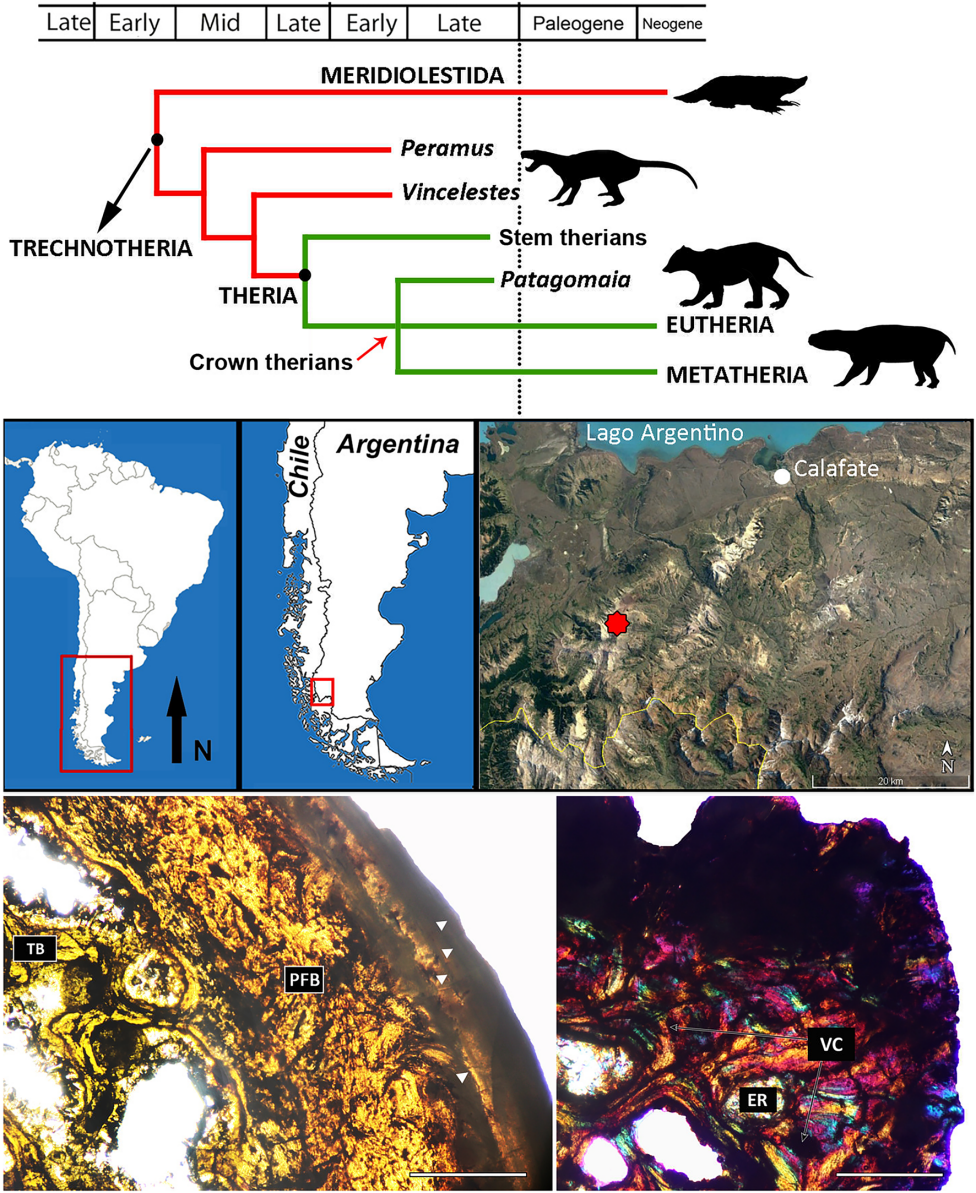
Simplified time-calibrated cladogram showing the phylogenetic affinities of *Patagomaia chainko*, geographic location and paleohistological images. The simplified cladogram shows our interpretation unifying the three analyses conducted (see Supplementary Information 5). Map showing the fossil locality. The specimen here reported was recovered at the new site located at S 50° 30′ 39.888″ and W 72° 33′ 18.035″, close to the *Isasicursor* 2 site (marked with a red star) in the Maastrichtian Chorillo Formation. Transverse section of the femur (left) showing the External Fundamental System (white arrowheads); and tibia (right) in polarized light with lambda compensator. ER, Erosion room; PFB, parallel-fibered bone tissue; TB, trabecular bone; VC, vascular canal. Scale bar: 0.75 mm.

Available elements show that they belong to a medium-sized mammal, comparable in size to a the canid *Lycalopex culpaeus*. Following methodologies described in the SI, we estimated a body mass of ~ 14 kg for the holotype specimen of *Patagomaia chainko* (Supplementary Information 4; Supplementary Table 1; Supplementary Fig. 16). The histology of the femur and tibia of *Patagomaia* reveals that the cortex is composed of parallel-fibered and lamellar bone tissue and sparse/moderate density of longitudinal canals, closely resembling some Mesozoic eutherians (e.g., *Barunlestes*, *Zalambdalestes*)^58^. The presence of a well-defined External Fundamental System indicates the somatic maturity of the holotype of *Patagomaia chainko* (Fig. 2; Supplementary Information 2). This suggests that the size and body mass estimated here for the holotype of *Patagomaia chainko* likely represents the maximum ones that this animal could have reached.

## Discussion

To analyse the phylogenetic affinities of *Patagomaia chainko*, it was scored into three different comprehensive mammalian data matrices^13,59,60^. The incomplete nature of the holotype specimen, plus the fact that most of these datasets heavely rest on skull and teeth information, we were able to score just a small number of characters (less than 2.5%) for *Patagomaia*. However, the analyses consistently recover *Patagomaia* within Theria (Supplementary Information 5; Supplementary Figs. 17, 18 and 19). Synapomorphic features of Theria^7,44,47,52^ present in *Patagomaia* are: acetabulum completely fused and devoid of a dorsal emargination; femur with well-differentiated and medially-oriented head and neck; lesser trochanter small and posteroventrally displaced; distal end of femur with symmetrical articular condyles and reduced epicondyles; proximal end of tibia with symmetrical articular facets (in agreement with the condition of distal femoral condyles). This set of characters clearly distinguishes *Patagomaia* from non-therian mammals of the Southern Hemisphere, such as meridiolestidans (i.e., *Necrolestes*), multituberculates^50^, and gondwanatherians (i.e., *Adalatherium*)^13^.

It is worth noting that, although *Patagomaia* reveals therian affinities, it differs from Paleogene South American representatives of this clade (e.g., notoungulates, litopterns, astrapotherians, xenarthrans, and sparassodonts) in having a shallow patellar groove delimited by poorly defined crests that do not form a deep trochlea. In sum, *Patagomaia* does not exhibit morphological features that may ally it with any of the mammalian clades (i.e., Gondwanatheria, Dryolestoidea, Marsupialia, Ungulata) frequently recorded in Cretaceous and early Paleogene beds from South America. The prevailing view has been that the early evolutionary radiation of therian mammals occurred in the Northern Hemisphere^15–17^ although it has overlooked the occurrences of therians and stem-therians in the Cretaceous of Africa, Madagascar, India and South America^27–33^. In this context, *Patagomaia* constitutes an important addition to the meagre record of Mesozoic therians from Gondwana and indicates that some stages of early therian evolution occurred in the Southern Hemisphere^19–26^.

We estimate that the body mass of the holotype individual of *Patagomaia* ranged between 2.6 and 26 kg, with an average estimate of approximately 14 kg (a value obtained using the mean of 14 different regressions; see Materials and Methods and Supplementary Information 4). Even the smallest estimates would place it among the larger Mesozoic mammals, while the average and higher estimates exceed by far those of the largest Mesozoic mammals previously known: the Early Cretaceous Chinese eutriconodont *Repenomamus* (approximately 10 kg), and the Late Cretaceous gondwanatherian *Vintana* (8.9 kg). This would make *Patagomaia* the largest known Mesozoic mammal.

Most mammalian taxa recorded in Campanian–Maastrichtian assemblages of the Northern Hemisphere have an estimated body mass below 100 g, and only 1% of them are estimated to have reached a body mass of 1 kg^61^. In sharp contrast, at least 17 valid mammalian taxa (mainly belonging to Monotremata, Gondwanatheria and Meridiolestida) are known from Patagonian faunal assemblages, eight of which surpass 1 kg in body mass (see Supplementary Information 4). Similarly, large-bodied (much larger than 1 kg) mammals such as *Vintana sertichi* and *Adalatherium hui* are known from the latest Cretaceous of Madagascar^53^. This suggests that the evolution of larger body size among Gondwanan mammalian taxa began prior to the end-Cretaceous mass extinction event, thus preceding their Laurasian relatives in reaching large body size by at least 5 million years.

This new discovery demonstrates that Late Cretaceous mammalian faunas from South America were taxonomically diverse, not only including gondwanatherians, dryolestoids and monotremes, but also early therians. *Patagomaia* also reveals that the evolution of large body size among Late Cretaceous mammals was more complex than previously understood.

## Materials and methods

### Description

We follow the terminology of postcranial anatomy and myology used by Muizon^38^, Argot^39,40,62,63^, Fostowicz-Frelik^64^, Gambaryan et al.^65^ and Warburton et al.^66^. Paleohistological methods are explained in the Supplementary Information 3.

### Body mass estimation

Estimates of the body mass of *Patagomaia* were made by taking measurements of the postcranial remains, using regressions that have already been used in other fossil mammals. Measurements were taken with a digital caliper. In addition, other regressions, based on cranial and dental measurements already published by other authors, were used to calculate the body mass of other mammals from the Cretaceous of South America. The averages of all regressions were calculated and compared with the results obtained in *Patagomaia*. The methodology used for the body mass estimations is explained in the Supplementary Information 4.

### Phylogenetic analyses

Three data matrices were used with the aim of testing the phylogenetic position of *Patagomaia chainko* among mammals. We chose these data sets because they are very complete and updated matrices, and each one is focused on different groups of Mesozoic mammals (see Supplementary Information 5).

## Supplementary Information

Supplementary Information.

## Data Availability

The datasets analyzed during the current study are included in this published article (and its Supplementary Information file).
