# Supplementary material for: A large therian mammal from the Late Cretaceous of South America
Source: Sci Rep. 2024 Feb 3;14:2854. doi: 10.1038/s41598-024-53156-3 (PMC10838296; doi:10.1038/s41598-024-53156-3)
Supplement: Supplementary file 1 — Supplementary Information. [file 41598_2024_53156_MOESM1_ESM.pdf]

SUPPLEMENTARY INFORMATION

A LARGE THERIAN MAMMAL FROM THE LATE CRETACEOUS OF SOUTH AMERICA

Nicolás Chimento[1,3], Federico L. Agnolín[1,2,3], Jordi García-Marsà[1,3], Makoto Manabe[3], Takanobu Tsuihiji[4] & Fernando E. Novas[1,3]

[1]Laboratorio de Anatomía Comparada y Evolución de los Vertebrados (LACEV), Museo Argentino de Ciencias Naturales "Bernardino Rivadavia" (MACN-CONICET), Av. Ángel Gallardo 470, C1405DJR Ciudad Autónoma de Buenos Aires, Argentina.

[2]Fundación de Historia Natural "Félix de Azara", Departamento de Ciencias Naturales y Antropología, CEBBAD - Universidad Maimónides, Hidalgo 767, C1405BDB Buenos Aires, Argentina.

[3]Consejo Nacional de Investigaciones Científicas y Técnicas (CONICET)

[4]National Museum of Nature and Science; 4-1-1 Amakubo, Tsukuba 305-0005, Japan.

[5]Department of Earth and Planetary Science, The University of Tokyo; 7-3-1 Hongo, Bunkyo-ku, Tokyo 305-0005, Japan.

This PDF file includes:

SI 1. Extended description

SI 2. Comparative figures

SI 3. Paleohistology

SI 4. Body mass estimation

SI 5. Phylogenetic analyses

SUPPLEMENTARY INFORMATION 1. EXTENDED DESCRIPTION

Pelvic girdle. The pelvic girdle consists of two anterior fragments of preacetabular iliac blade, a complete acetabulum, with its iliac, ischiadic and pubic parts well fused, and a fragment of ischiatic blade (SF1a-b).

The preacetabular lamina shows a wide acetabular crest, separating the dorsal gluteal fossa and the ventral iliac fossa, both narrow and poorly marked. At the anterior end of the iliac blade there is a subcircular, slightly concave contour surface, possibly marking the bearing surface of the *sartorius* muscle. In dorsal view, at the anterodorsal end there is a marked tuberosity (anterodorsal iliac spine). Posteriorly, a dorsal iliac crest is projected. On the ventral side, there is a poorly marked anteroventral tubercle, from which projects a ventral iliac crest, less extended than the dorsal iliac crest. The iliac fossa is more dorsoventrally extended than the gluteal fossa, which is rather long anteroposteriorly and low. The medial surface is flat and slightly concave, and exhibits roughness, which could correspond to the articulation surface with the sacrum. This describes morphology resembles the rod-like shaped, elongate and not expanded anterior end ilia of many mammals[1,2,3,4,5].

The left acetabulum shows firmly fused ischium, pubis and ilium, lacking any suture traces (see SF3). The base of the ilium is dorsoventrally 19.22 mm and is subtriangular in cross-section. The lateral surface of the iliac branch shows subtriangular bump, that is possibly the insertion for the *rectus femoris* muscle[1]. This is dorsally delimited by a concave surface, possibly marking the posterior end of the insertion area of the *gluteus minimus* muscle (gm).

On the ventrolateral side of the iliac branch there is a pronounced concavity, limited posteriorly by the anterior edge of the acetabulum. This concavity may correspond to the posterior end of the insertion area of the iliac muscle (mi). The acetabulum is

subcircular in outline, with a complete, broad lunate surface (ls), posteroventrally open by an acetabular notch. The maximum length of the acetabulum is 16.54 mm by a height of 16.79 mm. The acetabular rim (ac) is robust and well-marked. The anterior edge of the acetabulum is more laterally projected than the rest of the margin. The dorsal side of the acetabulum is closed, and in dorsal view it is transversely wide. In the center of the acetabulum there exists an acetabular fossa, which differs from the lunate surface in that it exhibits a rough texture. The acetabular notch is posteriorly extended by a deep, well-marked canal, with a high ventral border and dorsally bounded by the posterior margin of the acetabulum.

Ventral to the acetabulum, there exists the base of the pubic ramus which is transversely flattened and anteroposteriorly narrower than the diameter of the acetabulum. There exists an anteroventral projection representing the base of the iliopubic eminence (ipe) (possibly for the *psoas minor* muscle, pm).

The posterior margin of the pubic ramus forms the anterior border of the obturator foramen. The latter is at the level of the posterior border of the acetabulum, suggesting that the obturator foramen would have been posterior to the acetabulum. The ventral border of the ischial branch and the posterior border of the pubic branch mark the anterior margin of the obturator foramen, which would have been relatively large, considering the separation between both branches. On the medial side of the pubic branch there is a wide groove, that extends along the entire length of the base of this branch. This groove could mark the vaginal artery and vein, its medial flange probably representing the separation between the both structures. Another possibility is that this groove corresponds to the *pars ilium* of the *obturator internus* muscle (piloi).

The posteromedial concavity located between the pubic and ischial branchs could corresponds to the insertion area of the *pars ischiopubicus* of the *obturator internus* muscle (pipoi). Posteriorly, the ischial branch is subtriangular in cross-section.

The dorsal view the anterior part of the acetabulum shows the insertion area of the *gluteus minimus* muscle. Posterior to the acetabulum there is a well-defined groove that extends at the mid-level of the acetabulum and which may correspond to the attachment of the *gemelli* muscle (ag).

The fragment of the ischial blade shows a subtriangular contour in section, with a flat lateral and dorsal surface, and concave medial surface. The flat, narrow and elongate dorsal surface, possibly is the attachment surface of the *gemelli* muscle.

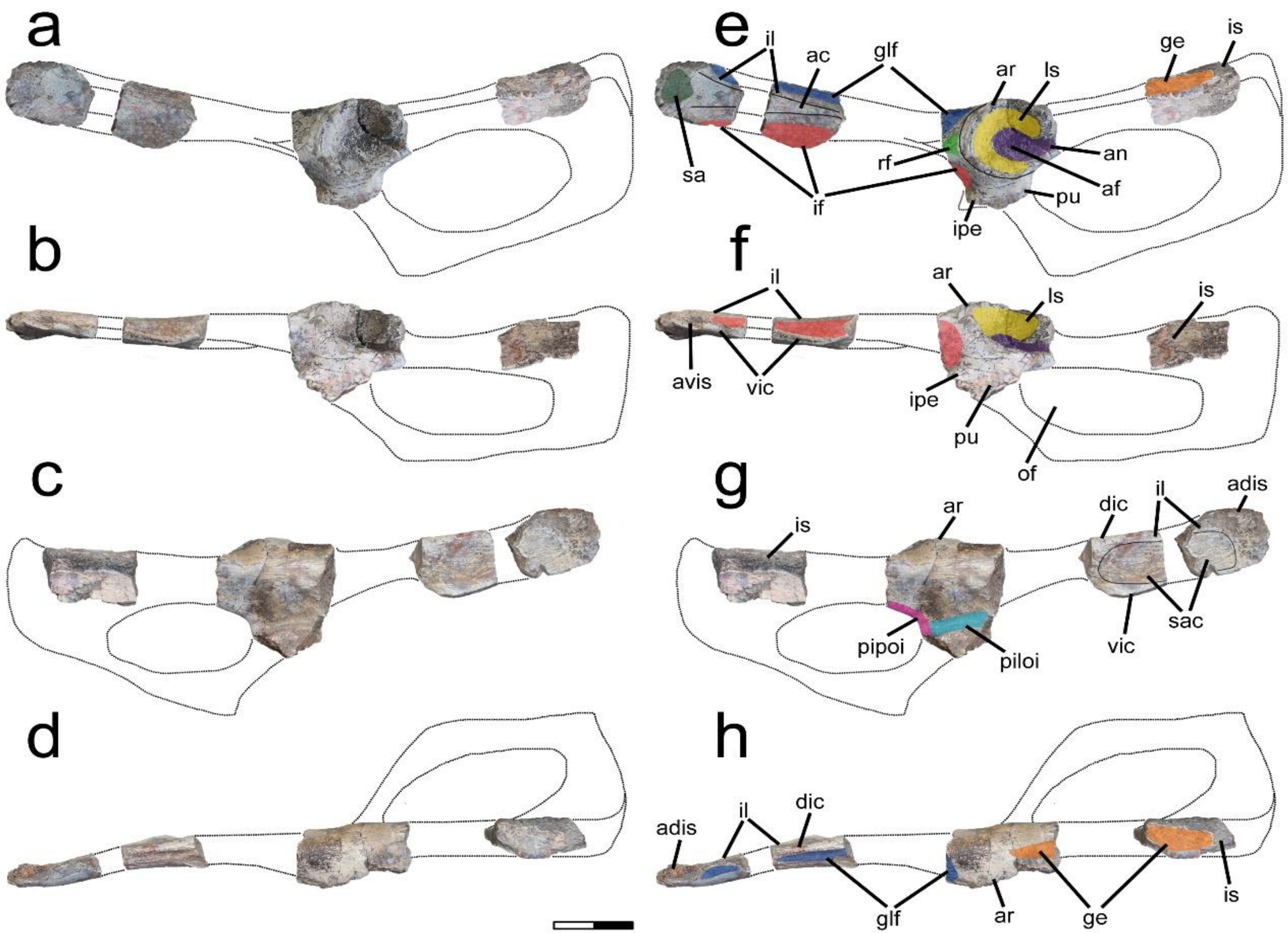

a
b
c
d
e
il
ac
glf
ar
ls
ge
is
sa
rf
an
af
pu
if
ipe
f
il
ar
ls
is
avis
vic
ipe
pu
of
g
is
ar
dic
il
adis
sac
vic
pipoi
piloi
h
adis
il
dic
glf
ar
ge
is

Supplementary Figure 1. Left pelvic fragments (left) of the holotype (MPM-PV-23365) and anatomical references (right) of *Patagomaia chainko* in lateral (a), ventral (b), medial (c), and dorsal views (d). **Abbreviatures**: **ac**, acetabular crest; **adis**, anterodorsal iliac spine; **af**, acetabular fossa; **an**, acetabular notch; **ar**, acetabular rim; **avis**, anteroventral iliac spine; **dic**, dorsal iliac crest; **ge**, attachment of *gemelli* muscle; **glf**, gluteal fossa; **if**, iliac fossa; **il**, ilium; **ipe**, iliopubic eminence (for the *psoas minor* muscle); **is**, ischium; **ls**, lunate surface; **pu**, base of pubis; **of**, obturator foramen; **piloi**, *pars ilium* of the *obturator internus* muscle; **pipoi**, *pars ischiopubicus* of the *obturator internus* muscle; **rf**, attachment of *rectus femoris* muscle; **sa**, attachment of *sartorius* muscle; **sac**, surface of the sacral articulation; **vic**, ventral iliac crest. Scale bar: 20 mm.

MPM-PV-23366 is a fragment of left acetabulum with part of the ischial blade. The acetabular morphology is equal of the holotype specimen, but the part of the ischial blade is more complete than in the latter. The isquial blade is robust and subtriangular in section. In this specimen it can be seen that the canal posterior to the acetabular notch is wide and extends just to the base of the ischial wing. The ventrolateral surface of the ischial blade is smoother than the rest, which could correspond to the attachment of the *obturator externus* muscle.

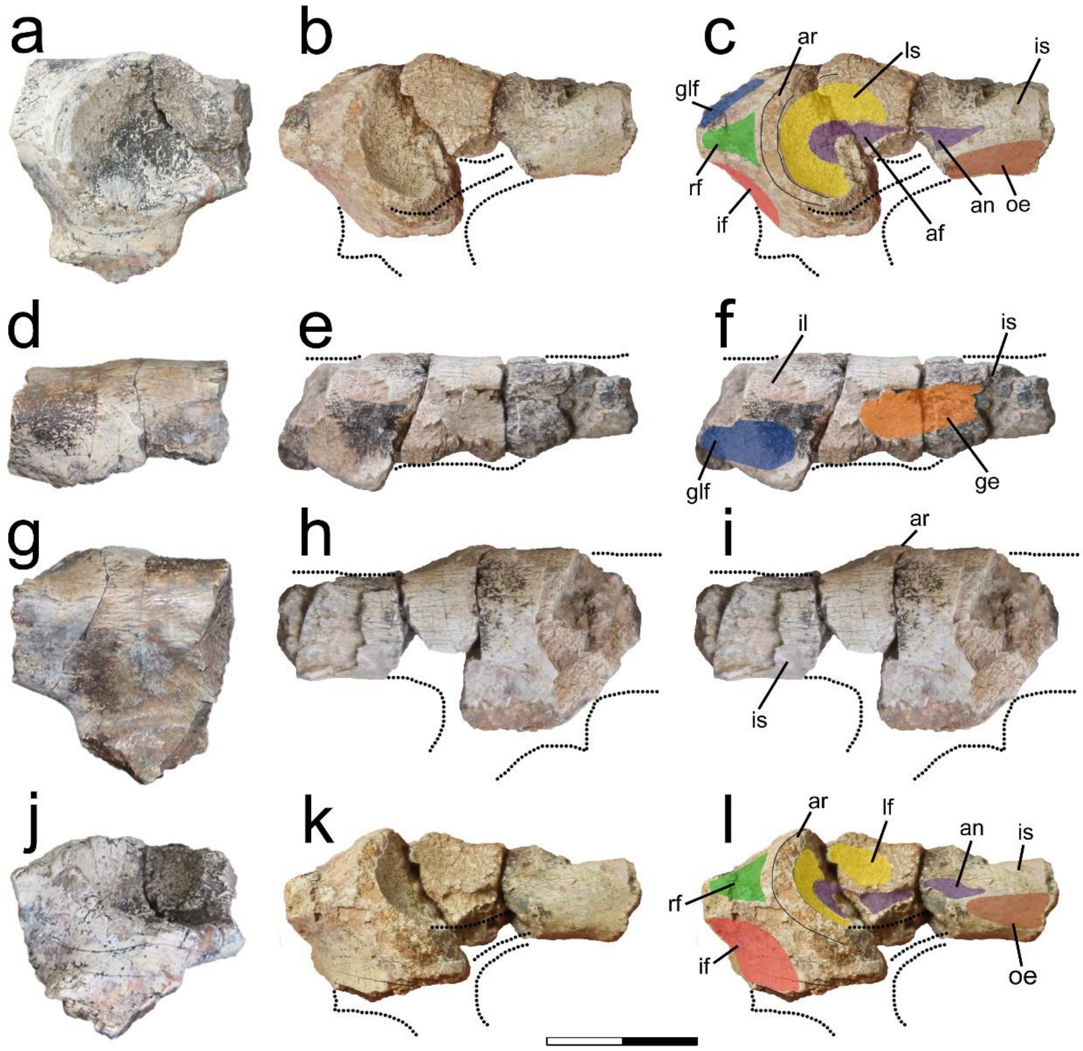

Supplementary Figure 2. Comparative figure of the MPM-PV-23366 (centrum and left) with the holotype specimen (right) in lateral (a-c), dorsal (d-f), medial (g-i) and ventral views (j-l). **Abbreviatures**: **oe**, attachment of obturator externus muscle; other abbreviatures as in SF 1. Scale bar: 20 mm.

Femur. The proximal half of the right femur has 36.4 mm of maximum transverse width. The proximal end measures 31.4 mm lateromedial diameter and 25 mm anteroposterior diameter. The transverse diameter of the diaphysis is about 29 mm. The femoral head measures 16.8 mm in lateromedial diameter, 14.4 mm in proximodistal diameter and 15.5 mm in anteroposterior diameter.

The femoral head is subspherical in shape, with a smooth surface, and a well-marked *fovea capitis*. The femoral head is projected medially and anteriorly with respect to the proximodistal axis of the femur. It presents a marked neck, with some striations on the anterior side. On the lateral side, the base of the greater trochanter is observed. In posterior view, a semicircular ridge is present at the middle of the femoral body, corresponding to the area of insertion of the *quadratus femoris* muscle[1,6], which delimits the distal margin of the trochanteric fossa. Laterally, a robust great trochanter is observed. This trochanter has a major axis oriented anteroposteriorly, so it is slightly compressed lateromedially, and less robust than the femoral head. The proximal border of the greater trochanter is at the same level as the femoral head. The entire trochanter is projected posteriorly. The greater trochanter has a smooth posterior surface, possibly corresponding to the insertion area of the *gluteus medius* muscle. On the medial side of the greater trochanter there is a proximodistal ridge, corresponding to the trochanteric crest. Medially, the ridge of the *quadratus femoris* muscle ended at the lesser trochanter, which is represented by its base. It was small and not medially extended. The lesser trochanter was distally positioned and well-separated from the femoral head. It should be barely observed in anterior view. On the lateral side, the ridge for the *quadratus femoris* muscle projects proximolaterally to the base of the greater trochanter. Proximal to this ridge there exists a concave surface, which marks the distal limit of the intertrochanteric fossa. In medial the lesser trochanter was posteriorly projected. Distal to the base of the lesser trochanter there extends a sharp ridge that marks the limit between the posterior and medial sides of the femur, which form an angle of about 90°. On the medial surface, distal to the lesser trochanter, there is a concave surface, probably where the *iliopsoas* muscle complex would be inserted.

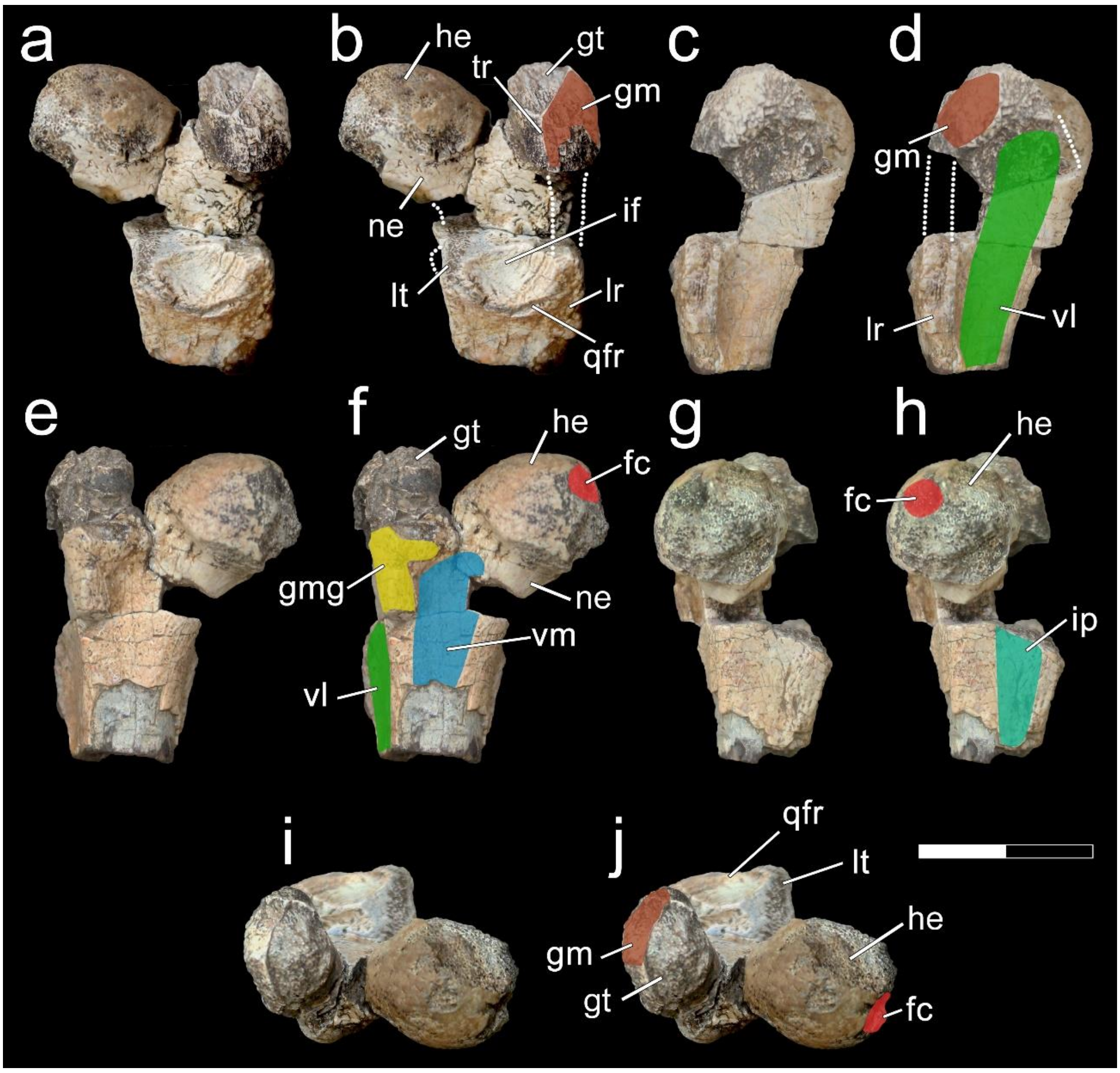

Supplementary Figure 3. Proximal end of the right femur of the *Patagomaia chainko* holotype (MPM-PV-23365) in posterior (a-b), lateral (c-d), anterior (e-f), medial (g-h) and proximal view (i-j). Scale bar: 20 mm. **Abbreviatures**: **fc**, fovea capitis; **he**, head; **gm**, attachment of *gluteus medius* muscle; **gmg**, attachment of gluteal muscle group; **gt**, great trochanter; **if**, intertrochanteric fossa; **ip**, attachment of iliopsoas muscle complex; **lr**, lateral ridge; **lt**, lesser trochanter; **ne**, neck; **qfr**, quadratus femoris ridge; **tr**, trochanteric ridge; **vl**, attachment of *vastus lateralis* muscle; **vm**, attachment of *vastus medius* muscle. Scale bar: 20 mm.

The base of the greater trochanter is subtriangular in cross-section. Along its anterior edge, the base of the greater trochanter exhibits a broad and transversely oriented ridge, and a thick

and proximodistally projecting crest, both showing a very rough surface. These ridges may correspond to the area of the attachment of the gluteal muscle group (*superficial gluteus, g. medius, g. profundus, g. minimus*)[1,3,7]. The lateral surface is smooth, and the posterior border present a marked ridge of proximodistal direction. The proximal part of this ridge arises along with the ridge for the *quadratus femoris*, at the greater trochanter, and would end distally at the third trochanter. The lateral face and posterior face are well separated from each other, which could mark the muscular separation between the *vastus lateralis* muscle (on the lateral aspect), and the *vastus medius* muscle (on the anterior and medial aspect)[1,3,8].

In proximal view, the proximal end of the femur is subrectangular in contour, with well-defined anterior, posterior, medial and lateral faces. On the medial edge of the femoral head, a concave and subcircular, proximomedially oriented *fovea capitis* (i.e., the insertion cavity of the acetabular ligament) is present.

The diaphysis, is subcircular in cross-section, with a maximum anteroposterior length of 11.6 mm and a máximum transverse diameter of 12.3 mm.

The distal end of the femur measures 31.86 mm in maximum preserved length, 23.92 mm in maximum transverse diameter and 17.97 mm in maximum anteroposterior diameter. In cross-section, the diaphysis is subcircular in contour. The lateral and medial margins of the diaphysis are subparallel to each other, and are nearly continuous with the borders of the distal condyles, resulting in a symmetrical aspect of the distal end of the bone. In distal view, the lateral condyle is somewhat transversely wider than the medial one. There are no well-marked epicondyles. There is only a very small medial epicondyle (me), which is located at the proximomedial border of the medial condyle. Lateral to the medial epicondyle there is a small concavity for insertion of the *gastrocnemius medialis* muscle (fgm). The medial condyle is subcircular in outline, and appears more robust than the lateral condyle in posterior view. Its medial margin is slightly concave, due to the presence of the concavity for the tibial collateral ligament (tcl). On the lateral edge of the medial condyle there is a deep notch,

possibly for the insertion or the medial cruciate ligament (mcl). The lateral condyle is incompletely preserved, and is transversely wider than the medial one. It is subtriangular in outline, with a concave lateral edge for anchoring of the fibular collateral ligament (fcl). The proximal edge of the lateral condyle shows a strong concavity that marks the distal limit of a large, suboval depression, possibly for the insertion of the *gastrocnemius lateralis* muscle (gl). Between this depression and the intercondyloid fossa is a broad intercondyloid ridge (ir). This ridge is very broad and obliquely oriented with respect to the main axis of the bone. The proximal edge of the intercondyloid ridge extends from the lateral condyle to the center of the diaphysis, while the distal edge of this ridge extends from the lateral condyle to the medial one. This ridge separates the intercondylar fossa on the distal side from a smooth concavity on the proximolateral side, which could show the support surface of the *gastrocnemius lateralis* o *externus* muscle. The good development of this muscle could indicate that *Patagomaia* was preferably terrestrial or saltatorial[1]. The intercondyloid fossa is very deep, showing a marked surface for the lateral cruciate ligament (lcl). In anterior view a poorly marked patellar surface (ps) is present, being delimited by poorly defined lateral and medial ridges. The distal end is subrectangular in outline, with a transverse diameter greater than the anteroposterior diameter, and well-marked condyles. The medial condyle is more posteriorly projected than the lateral condyle. The medial condyle shows the insertion surface of the medial cruciate ligament (mcl). On the anterior side, a medial ridge (mr) is more anteriorly projected than the lateral ridge.

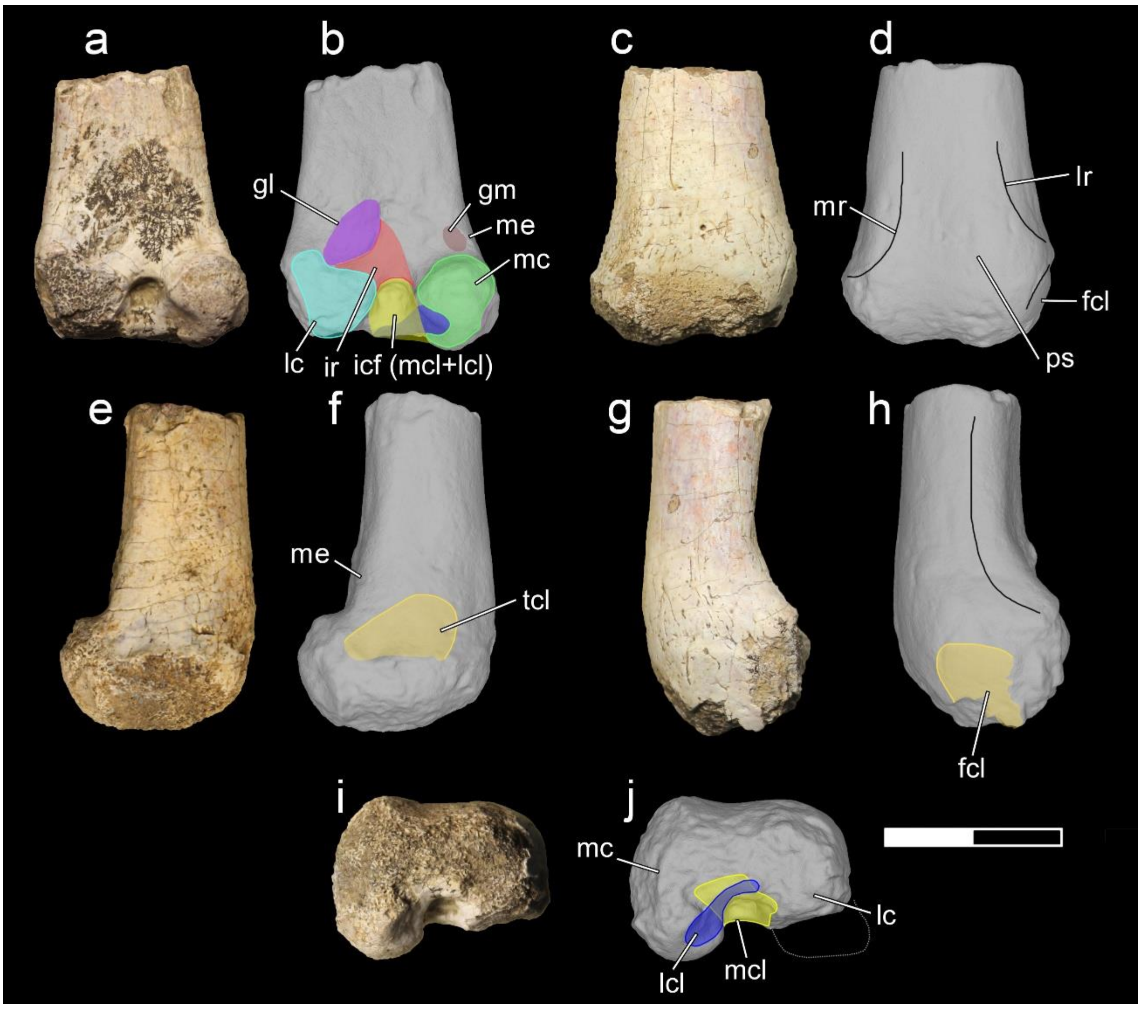

Supplementary Figure 4. Distal end of the left femur of the *Patagomaia chainko* holotype (MPM-PV-23365). Images (a,c,e,g,i) and 3D reconstructions (b,d,f,h,j) in posterior (a-b), anterior (c-d), medial (e-f), lateral (g-h) and distal views (i-j). **Abbreviatures: fcl**, fibular collateral ligament; **gl**, area of insertion of the *gastrocnemius lateralis* muscle; **gm**, area of insertion of the *gastrocnemius medialis* muscle; **icf**, intercondylar fossa; **ir**, intercondylar ridge; **lc**, lateral condyle; **lcl**, attachment of the *lateralis cruciate* ligament; **lr**, lateral ridge; **mc**, medial condyle; **mcl**, attachment of the *medialis cruciate* ligament; **me**, medial epicondyle; **mr**, medial ridge; **ps**, patellar surface. Scale bar: 20 mm.

In addition, a shaft fragment of a left femur from another specimen was preserved. This specimen (MPM-PV-23366) shows the same morphology of the type specimen (see SF 5).

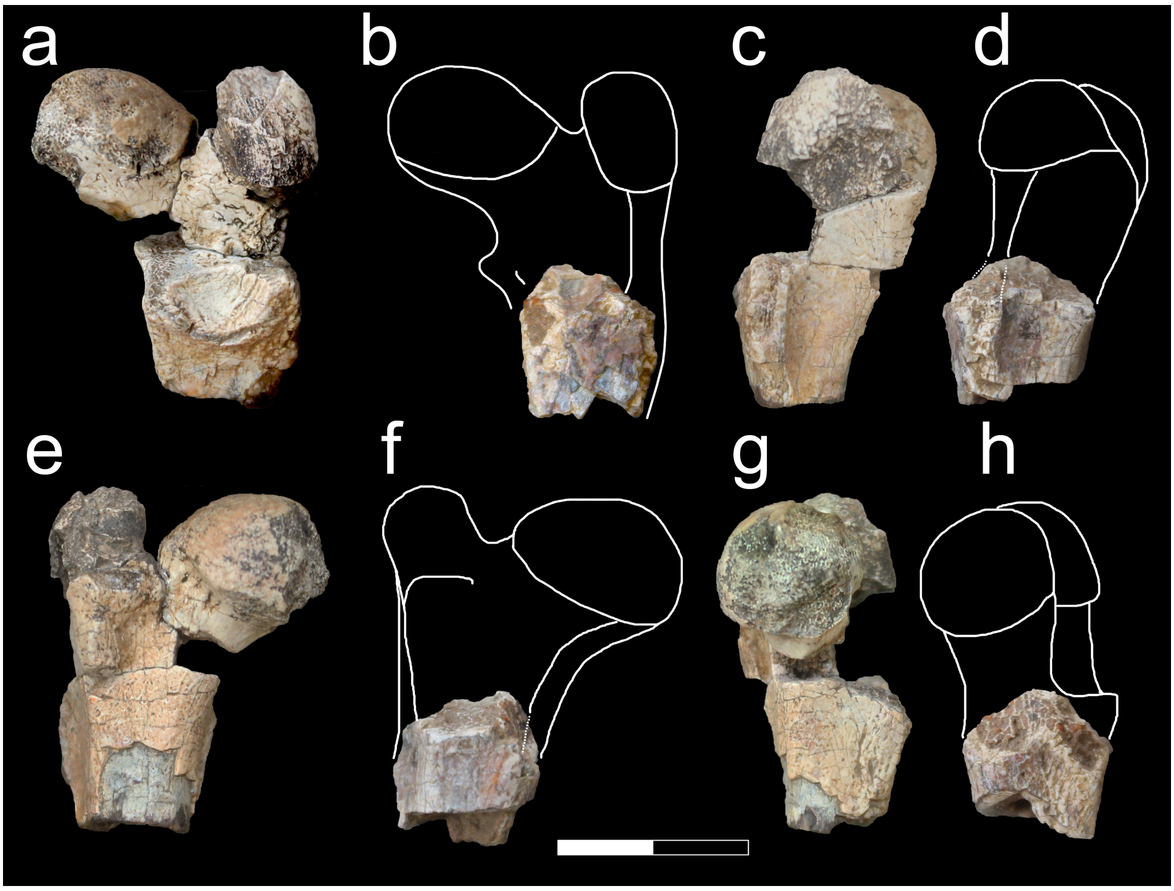

Supplementary Figure 5. Comparative figure of right femora of the holotype specimen (a,c,e,g) and referred specimen MPM-PV-23366 (b,d,f,h), in posterior (a-b), lateral (c-d), anterior (e-f) and medial views (g-h). Scale bar: 20 mm.

Tibia. The preserved tibia measures 48.03 mm long, with a transverse diameter of 19.15 mm, and the anteroposterior length being about 16.32 mm. In proximal view, its proximal end is subtriangular in outline. The medial facet is of subcircular outline, it is proximally concave and its lateral edge is proximally projected with respect to the medial edge, due to presence of an intercondylar eminence. The tibial tuberosity is bump-like and transversely broad, being distally continuous with a robust and broad tibial crest. On the medial side there is a marked popliteal line, which extends from the proximal end. The lateral surface of the diaphysis is excavated by an elongate fossa, possibly for the support of the *tibialis anterior* muscle. In

posterior view, a deep posterior fossa is observed, with a thick, well-marked medial ridge separating it into two surfaces, of which the lateral one is wider and deeper, possibly for accommodating the *flexor digitorum profundus* muscle[1,3,9-11]. Medially to the medial crest, the fossa is much narrower and poorly defined, probably representing the attachment surface of the *popliteus* muscle.

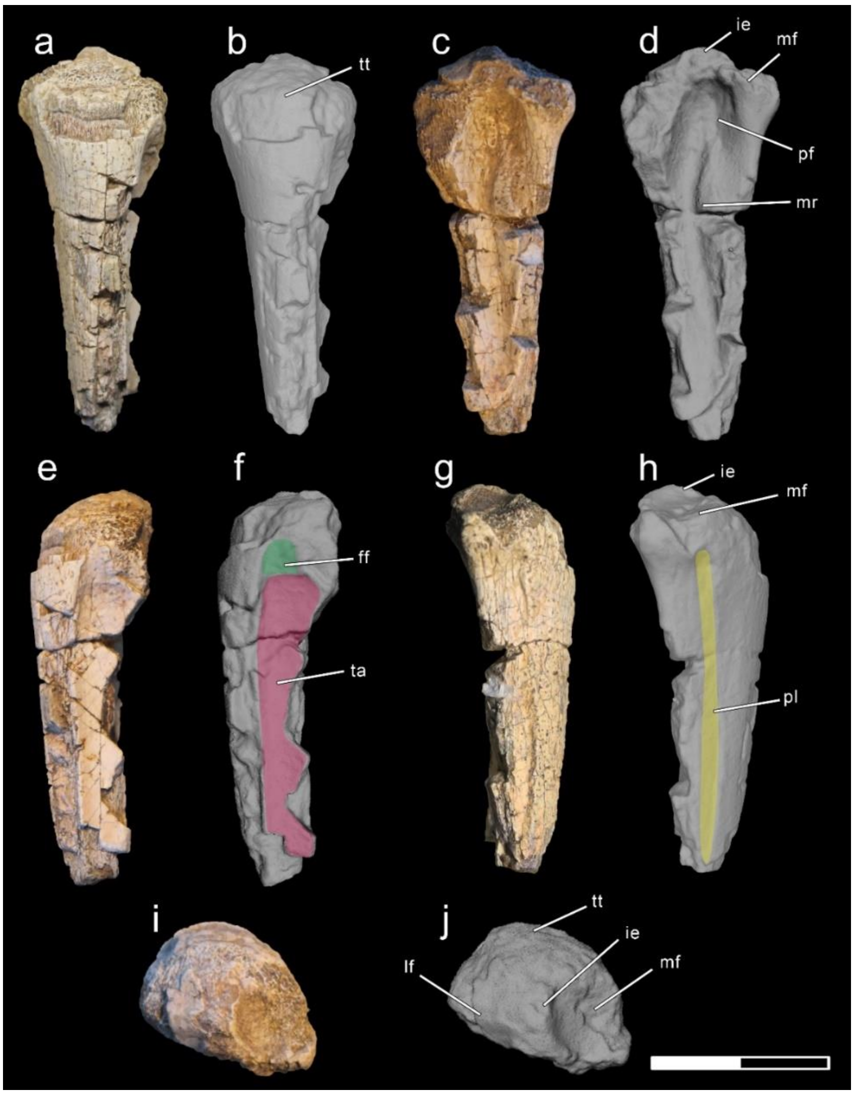

Supplementary Figure 6. Left tibia of the *Patagomaia chainko* holotype (MPM-PV-23365). Images (a,c,e,g,i) and squemes (b,d,f,h,j) in anterior (a-b), posterior (c-d), lateral (e-f), medial (g-h) and proximal views (i-j). Abbreviatures: **ff**, fibular facet; **ie**, intercondylar eminence; **lf**, lateral facet; **mf**, medial facet; **mr**, medial ridge; **pf**, posterior fossa; **pl**, popliteal line; **ta**, attachment of the *tibialis anterior* muscle; **tt**, tibial tuberosity. Scale bar: 20 mm.

Ulna. Only a distal end of the left ulna was preserved. Although the surface of the bone is somewhat corroded, the main structures can be noted. In lateral view, a robust styloid process is observed, projected distally, with a rounded distal surface and a subtriangular section. On the posterior border there is a robust lateral ridge marking the posterior limit of the lateral ulnar fossa, which could indicate the insertion area of the abductor pollicis longus muscle[6,12]. In medial view there is a robust pronator rigde, although the proximal part is missing, and the terminal part almost reaches the distal end.

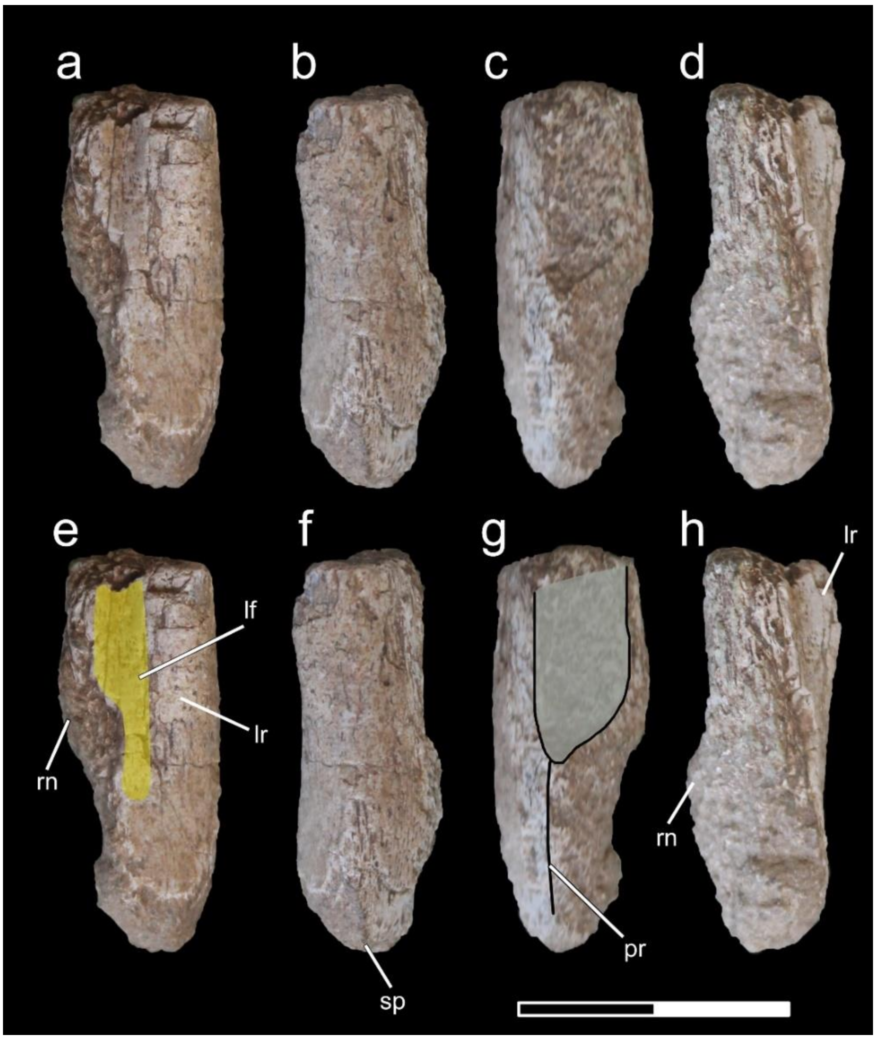

Supplementary figure 7. Distal end of the left ulna ot the *Patagomaia chainko* holotype (MPM-PV-23365), in lateral (a,e), posterior (b,f), medial (c,g) and anterior views (d,h). **Abbreviatures: lf**, lateral fossa; **lr**, lateral ridge; **pr**, pronator ridge; **rn**, radial notch, **sp**, styloid process. Scale bar: 20 mm.

SUPPLEMENTARY INFORMATION 2. COMPARATIVE FIGURES

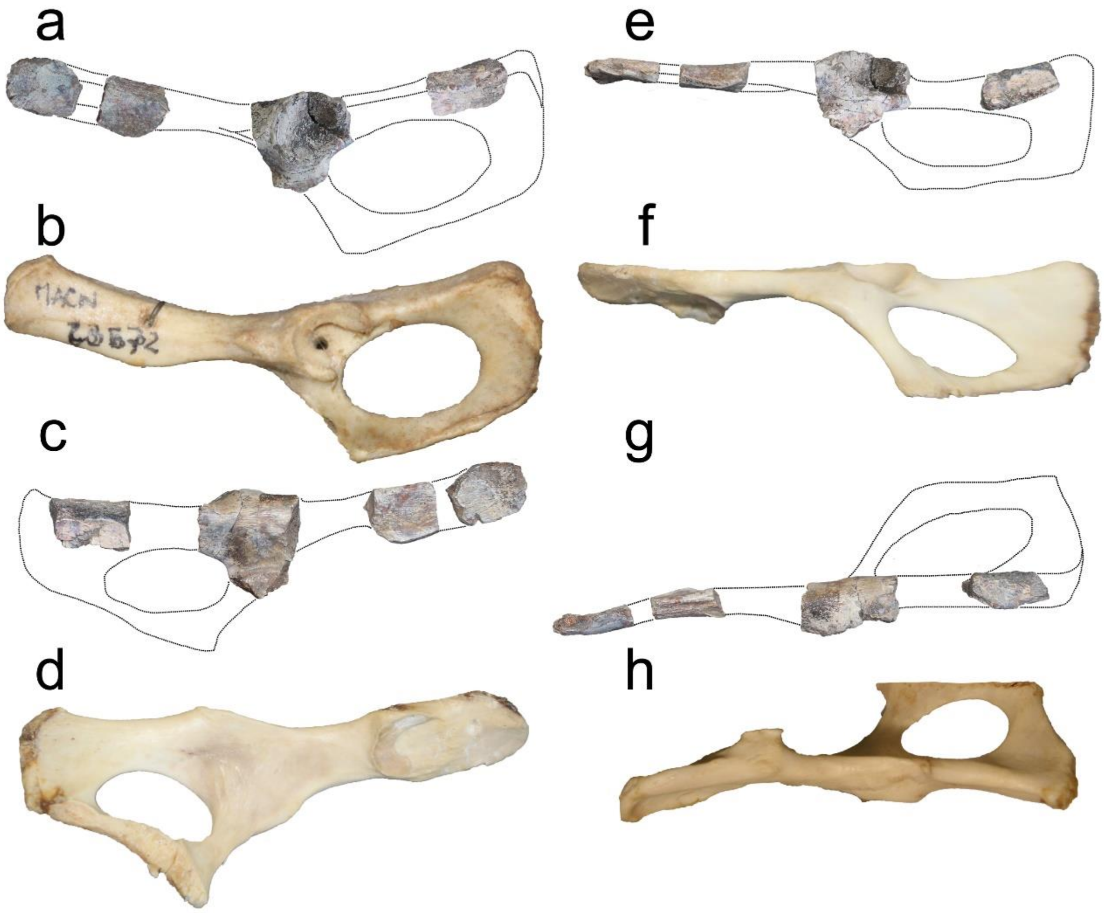

Supplementary figure 8. Left pelvic girdle of *Patagomaia* compared with therian mammals in lateral (**a-b**), medial (**c-d**), ventral (**e-f**) and dorsal views (**g-h**). References: **a,c,e,g**, *Patagomaia chianko* (MPM-PV-23365); **b**, *Dasyurus hallucatus* (MACN-Ma 23.572); **d,f,h**, *Leopardus pardalis* (MACN-Ma 49.343). Not to scale.

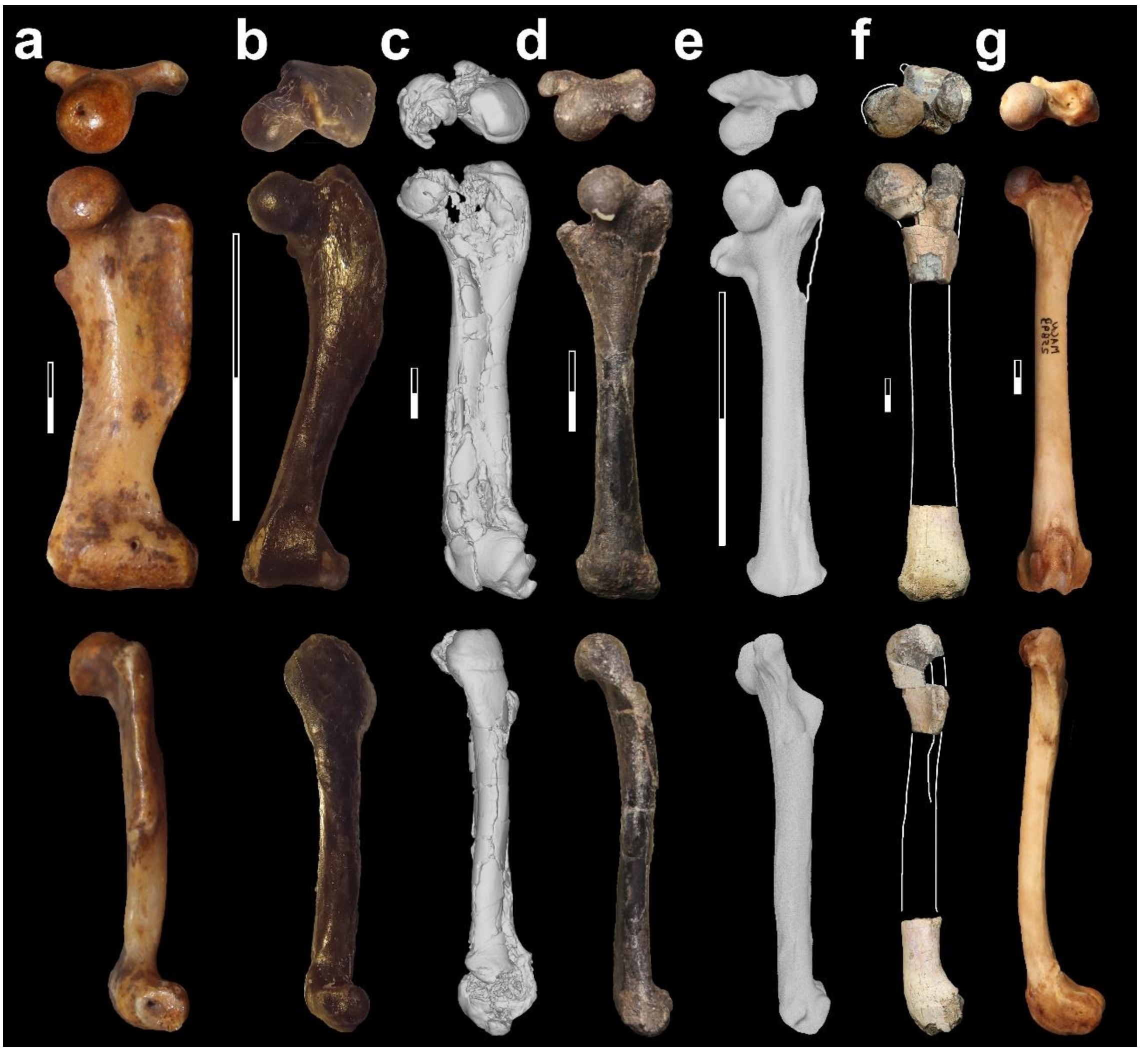

Supplementary figure 9. Left femora of selected mammals in proximal, anterior and lateral views. **References**: **a**, *Tachyglossus aculeatus* (MACN-Ma 7.6); **b**, *Necrolestes neuquenianus* (MACN A-5747, reverted); **c**, *Adalatherium hui* (UA 9030); **d**, *Vincelestes neuquenianus* (MACN-N 38); **e**, *Henkelotherium guimarotae* (Gui Mam 138/76); **f**, *Patagomaia chainko*, proximal end reverted (MPM-PV-23365); **e**, *Lycalopex gimnocercus* (MACN-Ma 25.893, reverted). Scale bars: 10 mm.

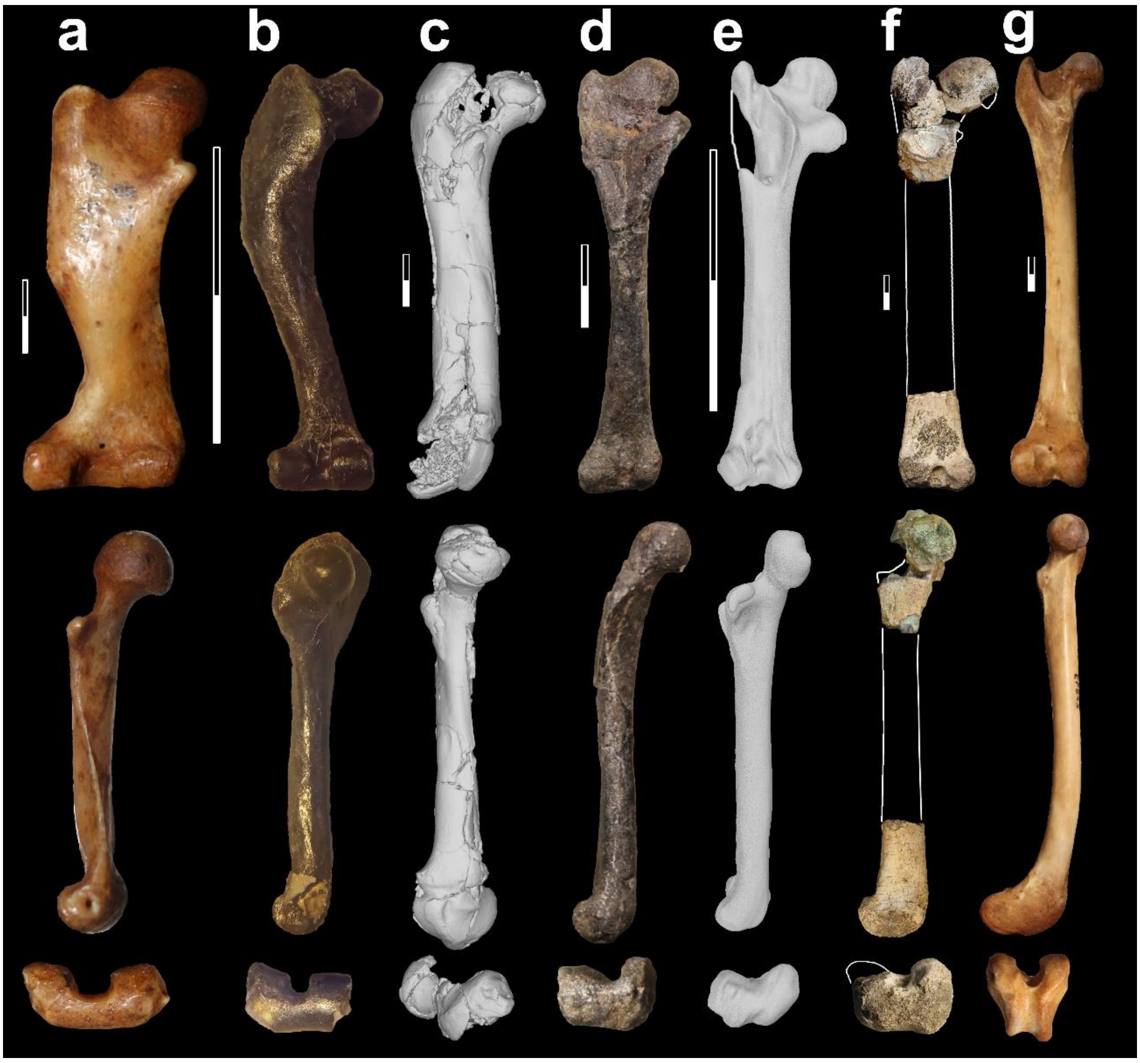

Supplementary figure 10. Left femora of selected mammals in posterior, medial and distal views. References as SF6.

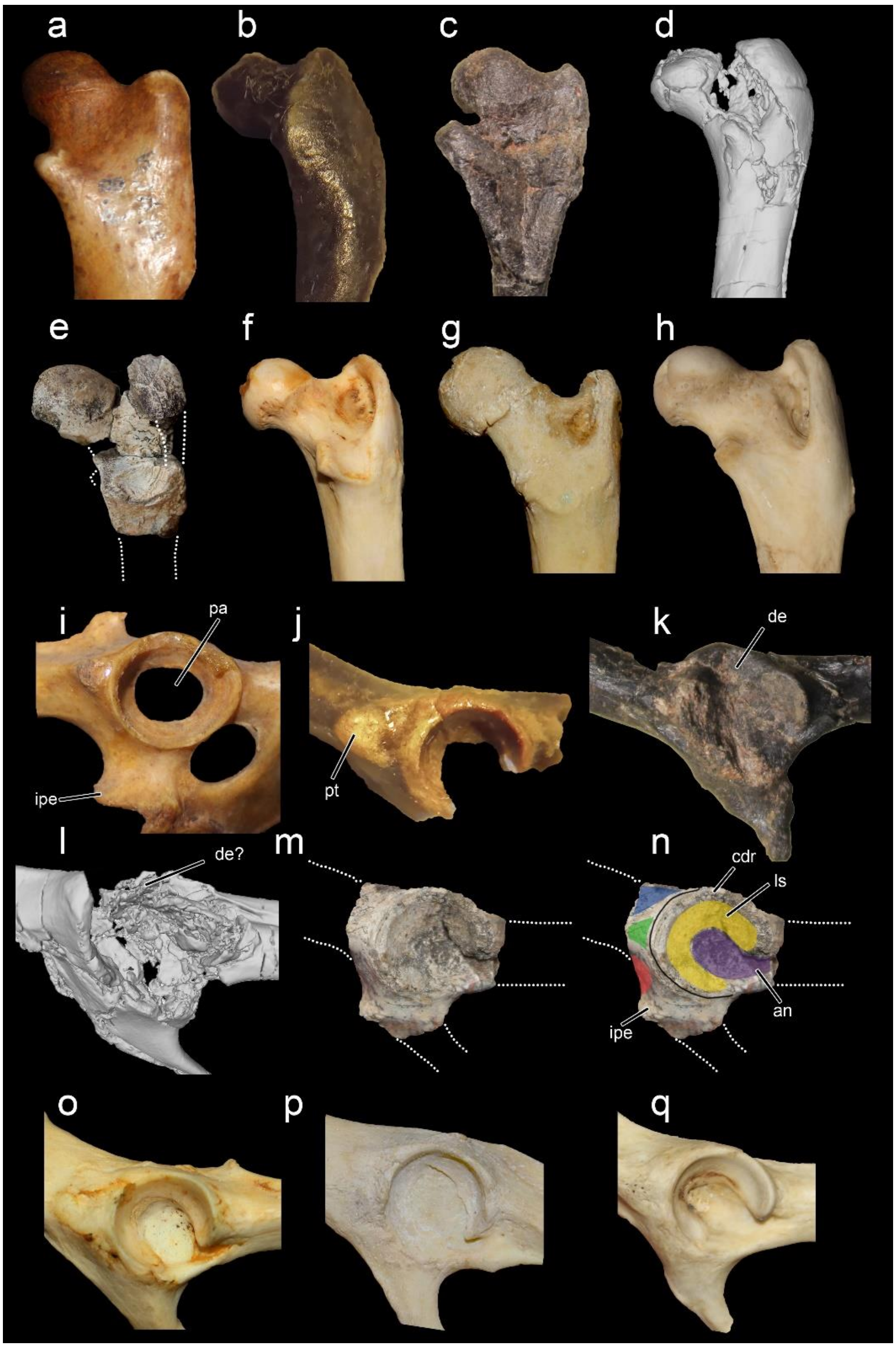

Supplementary figure 11. Rigth femora and left acetabuli selected mammals. **References:** **a,i**, *Tachyglossus aculeatus* (MACN-Ma 6.8); **b,j**, *Necrolestes neuquenianus* (MACN A-5747 and MACN A-5749); **c,k**, *Vincelestes neuquenianus* (MACN-N 38); **d,l**, *Adalatherium hui* (UA 9030); **e,m,n,** *Patagomaia chainko* (MPM-PV-23365); **f,o**, *Leopardus pardalis* (MACN-Ma 49.343); **g,p**, *Arctictis binturong* (MACN-Ma 43.67); **h,q,** *Procyon cancrivorus* (MACN-Ma 29.285). **Abbreviatures: an**, acetabular notch; **cdr**, complete dorsal rim; **de**, dorsal emargination; **ipe**, iliopubic eminence; **ls**, lunate surface; **pa**, perforate acetabulum; **pt**, preacetabular tubercle. Not to scale.

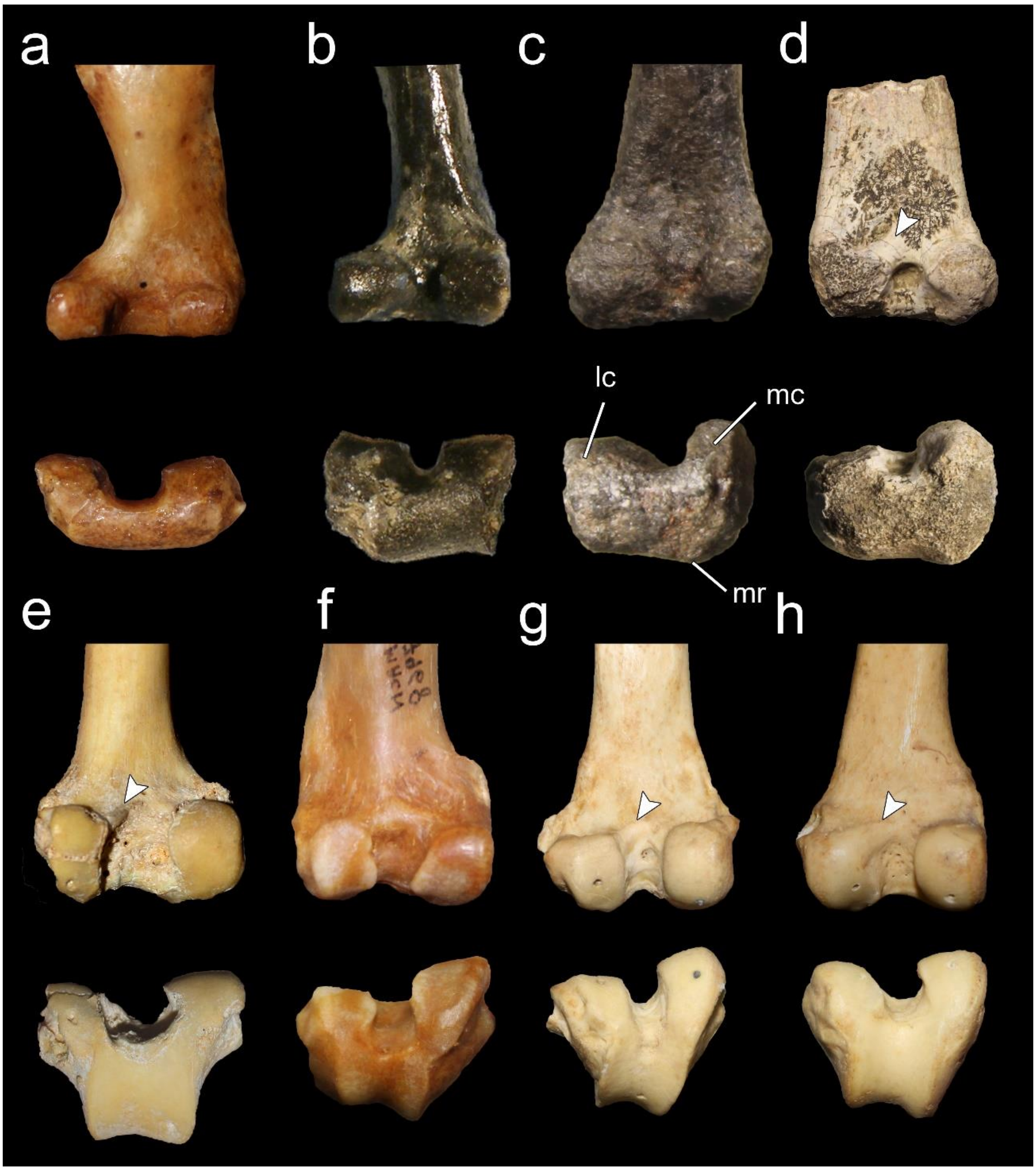

Supplementary figure 12. Distal ends of femora of selected mammals in posterior (upper) and distal views (lower). **References: a**, *Tachyglossus aculeatus* (MACN-Ma 6.8); **b**, *Necrolestes neuquenianus* (MACN A-5747); **c**, *Vincelestes neuquenianus* (MACN-N 38, reverted); **d**, *Patagomaia chainko* (MPM-PV-23365); **e**, *Arctictis binturong* (MACN-Ma 43.67, reverted); **f**, *Chaetophractus villosus* (MACN-Ma 27.968, reverted); **g**, *Hystrix cristata* (MACN-Ma 5.51); **h**, *Meles meles* (MACN-Ma 5.36, reverted). **Abbreviatures: lc**, lateral condyle; **mc**, medial condyle; **mr**, medial ridge. The arrows indicate the presence of the obliquely oriented intercondylar ridge in some mammals. Not to scale.

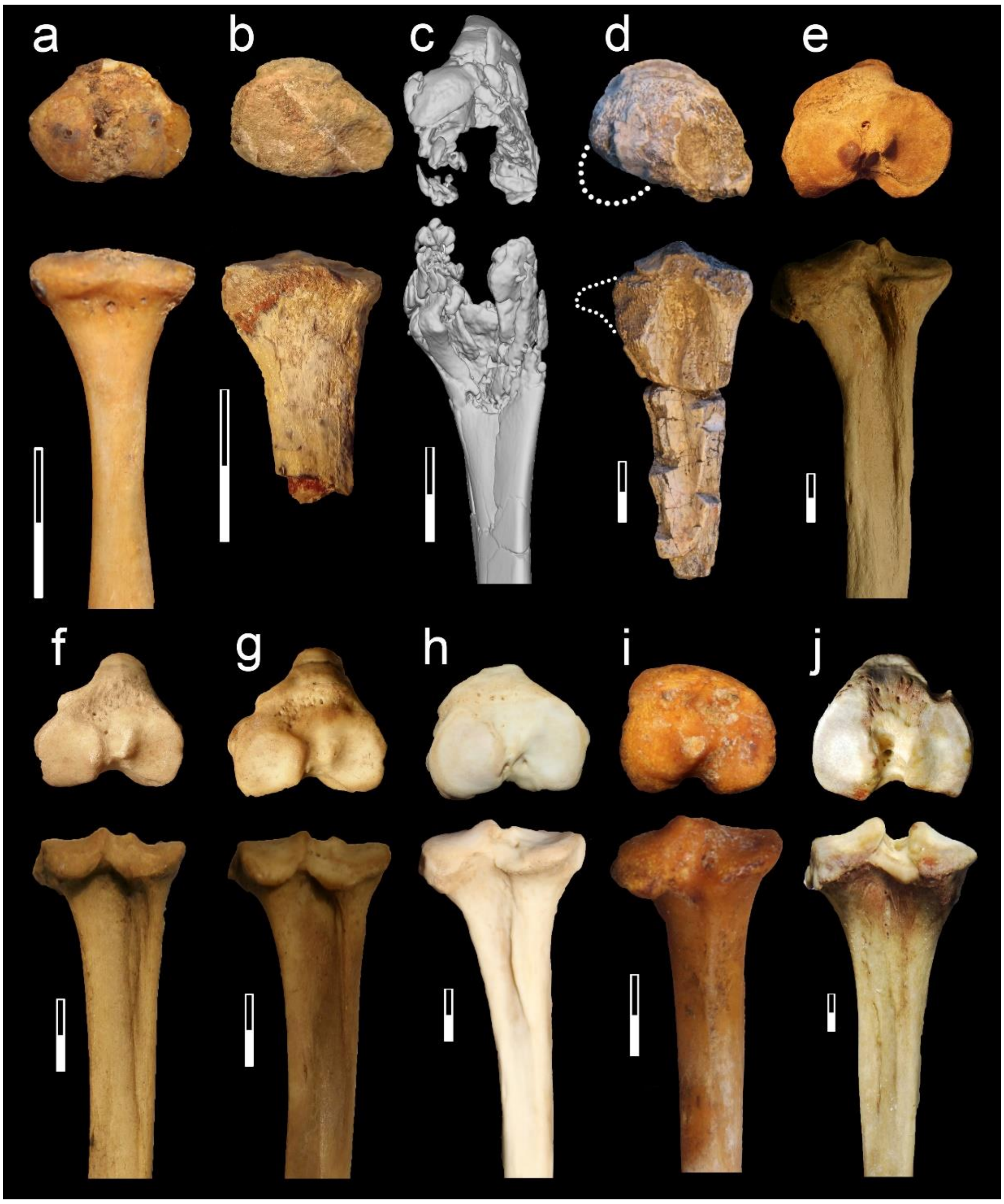

Supplementary Figure 13. Comparative figure of the left tibia of selected mammals, in proximal (upper) and posterior (lower) views. **References: a**, *Tachyglossus aculeatus* (MACN-Ma 6.8); **b**, *Vincelestes neuquenianus* (MACN-N 01); **c**, *Adalatherium hui* (UA 9030); **d**, *Patagomaia chainko* (MPM-PV-23365); **e**, *Panthera onca* (MACN-Ma 29.911, reverted); **f**, *Leopardus geoffroyi* (MACN-Ma 34.335); **g**, *Herpailurus yagouaroundi* (MACN-Ma 23.173, reverted); **h**, *Procyon cancrivorus* (MACN-Ma 29.285); **i**, *Lemur* sp. (MACN-Ma 23.785); **j**, *Hydrochoerus hydrochaeris* (MACN-Ma 30.582, reverted). Note de presence of the deep posterior fossa in felid species, and the low tibial tuberosity in *Vincelestes* (b) and many eutherian mammals (e.g. c,h,i,j). Scale bars: 10 mm.

SUPPLEMENTARY INFORMATION 3. PALAEOHISTOLOGY

Material and methods

Thin sections were prepared following the method outlined by Chinsamy and Raath[13]. The bones were embedded in a clear epoxy resin (Araldite© GY 279, catalysed with Aradur® hY 951) and left for 24 hours to set. They were cut into smaller blocks perpendicular to the long axis of the bone using a cut-off diamond tipped saw within a Ken 9025 grinding machine. One surface of each resin block was then affixed to a frosted petrographic glass slide using the same resin that was used for embedding and left to set for a further 24 hours. The sections were wet-ground to approximately 60 μm thick and polished using a Prazis APL-S polishing machine with abrasive papers of increasing grit size (P80, P120, P320, P400, P600, P1200, P1500, P2000, P3000). Samples were studied using a Zeiss Axio Scope.A1 petrographic polarising microscope under normal, polarised, and lambda light regimes.

Vascularisation in the samples has not been quantified. Therefore, the relative densities of vascular canals are assessed visually and described qualitatively, we follow the terminology of Warshaw[14], with modifications. The following three terms are used: (i) sparse vascularization, vascular canals are irregularly distributed, with avascular stretches between them that generally exceed three times the diameter of the canals; (ii) moderate vascularization, vascular canals are more regularly distributed, with the distance between canals frequently less than three times the diameter of the canals; (iii) dense vascularization, vascular canals are separated from each other by less than the diameter of two canals.

With respect to the ontogeny of the studied specimen, bone tissues exhibiting reduced vascularization, predominance of longitudinal canals and a lack of anastomoses, narrowing of zones deposited between successive Lines of Arrested Growth (LAGs), and formation of an External Fundamental System (EFS) at the bone perimeter are all indicative of a slowing growth rate in tetrapods, and are thus indirectly indicative of maturity[15-20]. Some of these traits are present in the material reported here.

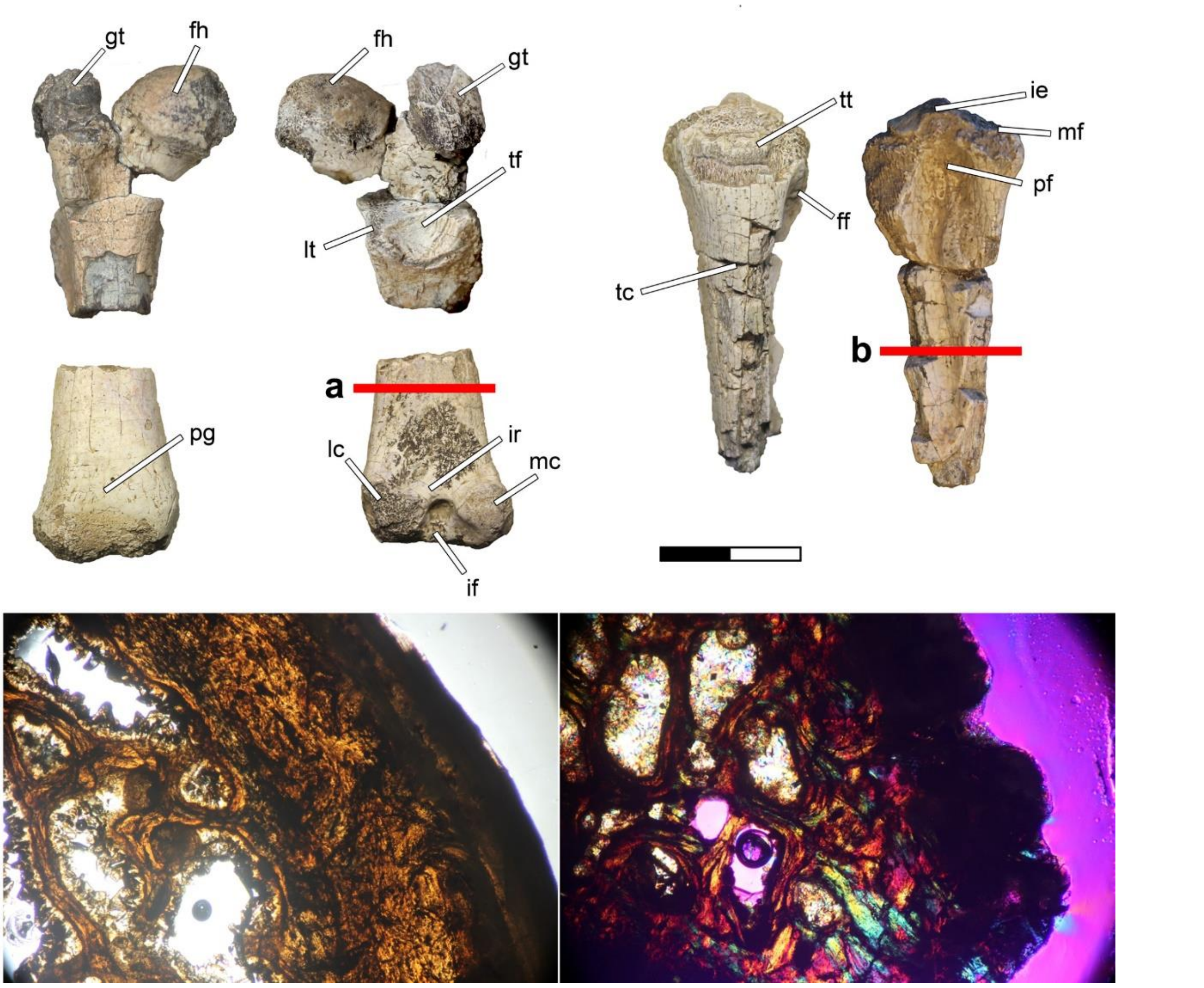

Supplementary figure 14. Preserved limb bones of *Patagomaia* (top) and location (a-b) of the sections of performed osteohistology (bottom). Scale bar: 20 mm.

Results

***Femur*.** The pyrolusite affected the subperiosteum of the cross section and some areas are fractured. Despite the affected areas, the microstructural features provide sufficient information.

The cortex is thin, the inner and middle cortex is constituted by parallel-fibred bone, and the subperiosteum is composed by lamelar bone tissue interrupted by one annulus and three closely spaced LAGs that apparently form an EFS. The inner and middle cortex shows moderate vascularization, consisting mainly of longitudinal vascular canals, and to a much

lesser extent, radial canals. The subperiosteal surface is avascular, suggesting, along with the EFS, an important decrease in growth and thus the somatic maturation of the specimen. The medullar cavity is infilled with thin trabeculae constituted by lamellar bone tissue.

Osteocyte lacunae with an oval morphology are distributed across the inner and middle cortical surface without any clear pattern. The osteocyte lacunae of the subperiosteum are elongated and equidistant. In the trabeculae, the morphology of the osteocyte lacunae is also elongated.

Tibia. The pyrolusite strongly affected the subperiosteum and some areas are fractured. The inner and middle cortex presents some fractures. Despite the affected area, the microstructural features of the inner and middle cortex are informative.

The cortex is thin and constituted by parallel-fibred bone with some erosion rooms (ER) in the inner cortex. The vascularization is sparse and consists of longitudinal vascular canals. There is no EFS. The medullar cavity is infilled with thin trabeculae constituted by lamellar bone tissue.

Osteocyte lacunae with an oval morphology are distributed across the cortical surface without any clear pattern. In the trabeculae, the morphology of the osteocyte lacunae is elongated.

Growth pattern and comparison with other taxa

Patagomaia shows significant differences in its growth patterns in relation to basal Probainognathia, such as *Chiniquodon* sp.[21] and *Trucidocynodon riograndensis*[22]. Both exhibit sustained growth, whereas *Patagomaia* shows cyclic growth in its femur. The vascularisation of *Patagomaia* is similar to *Chiniquodon* sp.[21], but much lower than *Trucidocynodon*[22], even though *Patagomaia* is larger than *Chiniquodon* and *Trucidocynodon*.

Patagomaia and *Probainognathus* both exhibit growth pattens that are indicative of cyclical and slow growth[23]. This contrasts with the more basal Probainognathia, and more closely resembles that of non-mammalian Prozostrodontia. However, within Prozostrodontia, the reduced cyclicity found specifically in Brasilodontidae show a trend towards decreased plasticity during development[21,22,24-27]. However, in contrast to *Probainognathus, Patagomaia* presents less developed cyclical growth and a denser vascularization.

The *Patagomaia* femur shows a subperiosteal annulus between the middle and outer cortex, making it comparable to the prozostrodontians *Prozostrodon brasiliensis* and especially *Irajatherium hernandezi* in its cyclic growth[27]. *Prozostrodon* and *Irajatherium* present wide zones of slow-growing bone tissue traversing the midcortex of the limb bones. However, prozostrodontians show signs of rapid growth in early ontogeny[27], while in *Patagomaia,* growth is even slower in early ontogeny, particularly in the tibia.

Patagomaia contrasts with *Tritylodon* in its vascularization, which is less developed than in *Tritylodon*. Some authors[22,26] suggested that *Tritylodon* presents highly vascularized, rapidly forming fibrolamellar bone tissue and sustained growth up to skeletal maturity due to the presence of peripheral lamellar bone containing multiple LAGs in the radius and fibula. The subperiosteal LAGs of *Tritylodon* resemble the EFS of the *Patagomaia* femur.

Patagomaia exhibits a similar cyclicity to brasilodontids, a sister taxon to Mammaliaformes, which exhibit woven-fibered and parallel-fibered bone tissue[27]. Brasilodontids present a slower growing bone tissue, present even during the early stages of ontogeny, with longitudinally oriented primary osteons[27], features similar to *Patagomaia*. Botha-Brink et al.[27] postulated than the relatively slower growth in *Brasilodon* and *Brasilitherium* may be related to their small body size compared with *Prozostrodon* and *Irajatherium*. However, *Patagomaia,* with a similar growth rate, is the biggest between all these taxa, suggesting other causes for their growth pattern (environmental, phylogeny, or both) independent of size. The growth patterns of *Patagomaia* are similar to those of the Early Jurassic mammalia form

Morganucodon and the Late Cretaceous multituberculate mammals *Kryptobaatar* and *Nemegtbaatar*[26].

When compared with mammaliaforms, *Patagomaia* microstructurally resembles *Morganucodon* in the density of vascularisation and cyclical growth[26]. However, early in ontogeny, *Morganucodon* sp. has woven-fibered bone tissue that is unknown in *Patagomaia*.

Basal Probainognathia (e.g., *Chiniquodon* sp., *Trucidocynodon*)[21,22] show growth patterns that indicate a decrease in growth plasticity. This condition differs from other derived non-mammaliaforms Probainognathia, *Morganucodon*, *Patagomaia,* and eutherian mesozoic mammals that show significant growth plasticity[24-27].

The mandibular cortex from the Paleocene multituberculate *Ptilodus* is seemingly made of "lamellar" tissue displaying simple vascular canals arranged radially and longitudinally[28]. These features resemble *Patagomaia*, nevertheless, in *Patagomaia* the major part of the cortex is parallel-fibered bone tissue.

Other multituberculates studied by Chinsamy and Hurum[26] differ widely from *Patagomaia* in their osteohistological features. *Nemegbaatar gobiensis* presents a small patch of periosteally woven bone tissue, suggesting initial periosteal cortical bone tissue formed rapidly. *Kryptobaatar dashzevegi* presents a cortex of woven bone tissue with primary osteons, suggesting a rapid rate of bone formation. In contrast, *Patagomaia* shows a slow rate from the beginning of its ontogeny.

Patagomaia resembles *Ornithorhynchus* in some osteohistological features. The analysis of *Ornithorhynchus*[26,28,29] suggests intraspecific variation. Chinsamy and Hurum[26] indicated that the cortex of *Ornithorhynchus* is composed of woven-fibered and fibrolamellar bone tissue, and that some areas are interrupted by lamellar bone tissue (forming annuli), but the cyclical growth is less developed in *Patagomaia*. The orientation and density of the vascular canals and primary osteons in *Ornithorhynchus* is similar to *Patagomaia*.

Patagomaia differs from *Tachyglossus aculeatus*[29] in that it has cyclical growth marks. The vascularization in *Tachyglossus* is longitudinal and the cortex is constituted by intermediate woven-fibered and parallel-fibered bone tissues[29]. Whereas in *Patagomaia,* the vascularization is lower and lacks woven-fibered bone tissue.

Patagomaia and *Didelphis* share some osteohystological features. *Didelphis* species are relatively small in size and the limb bone cortices are composed of parallel-fibered tissue[19,26,28-30]. A thin EFS of parallel-fibered or lamellar bone occurs in adults of *Didelphis,* and cyclical growth marks are rare in the long bone cortex[19,30]. These osteohistological features resemble *Patagomaia*. The vascular canals and primary osteons of *Didelphis* are longitudinal, oblique, or radial[29], and resemble *Patagomaia* in its vascular density. However, *Patagomaia* never forms woven-fibered bone tissue, but in *Didelphis,* towards the depth of the cortex, the bone matrix is woven-fibered type[19].

Patagomaia shares a similar vascular density and collagen-fiber organization with larger species of marsupials[29,30], where the primary osteons are oriented longitudinally or obliquely in approximately two-thirds of the cortex. Vascular density decreases toward the subperiosteum then disappears entirely, in a similar way to *Patagomaia*. The cortex consists of parallel-fibered tissue, like in *Patagomaia*. *Vombatus* and *M. rufogriseus* femora do not show a clear EFS but they display faint cyclical growth marks, resembling the EFS recorded for *Patagomaia*. However, in *M. fuliginosus,* the histological structure of bone in young specimens, and that found in the deep cortex of older individuals, consists of woven and parallel-fibered bone tissue with reticular vascular orientation[29], differing strongly from *Patagomaia*.

The microstructural features for *Didelphis*, *Vombatus* and *Macropus* suggest that marsupial long bones reveal the succession of two growth phases, a phase of fast growth followed by a long-lasting phase of slow growth[29]. This differs from *Patagomaia,* where the growth rate is slow even during the beginning of the ontogeny.

The histological features of *Patagomaia* resemble the eutherians *Zalamdalestes* and *Barunlestes*[26], however *Zalamdalestes* and *Barunlestes* do not record an EFS or similar subperiosteal structure. Chinsamy and Hurum[26] indicated that the growth rate among Mesozoic multituberculates and eutherians appeared slower compared to present-day monotremes and placentals. Nevertheless, unlike the multituberculate *Nemegtbaatar* and *Kryptobaatar*, which showed an initial high rate of bone formation composed by woven-fibered bone tissue, followed by a slower rate during later stages, the Mesozoic eutherians *Zalambdalestes* and *Barunlestes* show a slow rate of growth with growth marks.

Chinsamy and Hurum[26] proposed that, among eutherians, mammals developed rapidly and demonstrated a trend towards uninterrupted growth during the Paleocene, stimulating an increase in body size. Modern eutherians have retained the plesiomorphic condition of uninterrupted growth recorded in much more basal taxa. Developmental plasticity has a tendency to decrease in the more Eutherian-derived taxa[25,26]. However, studies on seasonal growth in ruminants seem to indicate that growth flexibility is a universal feature of animals, even those with endothermic metabolism[31]. Recently, Buffrenil et al.[29] indicated that there are no histological features unique to mammals, that all the tissues observed in mammal skeletons also occur in other tetrapods, and that tissues are influenced by the cascade of local or general morphogenetic processes that occur during bone growth and result in relatively complex structural patterns.

In sum, the histological features of *Patagomaia* strongly resemble the mesozoic Eutheria *Barunlestes* and *Zalambdalestes*[26], suggesting paleobiological and phylogenitcal affinities with these taxa.

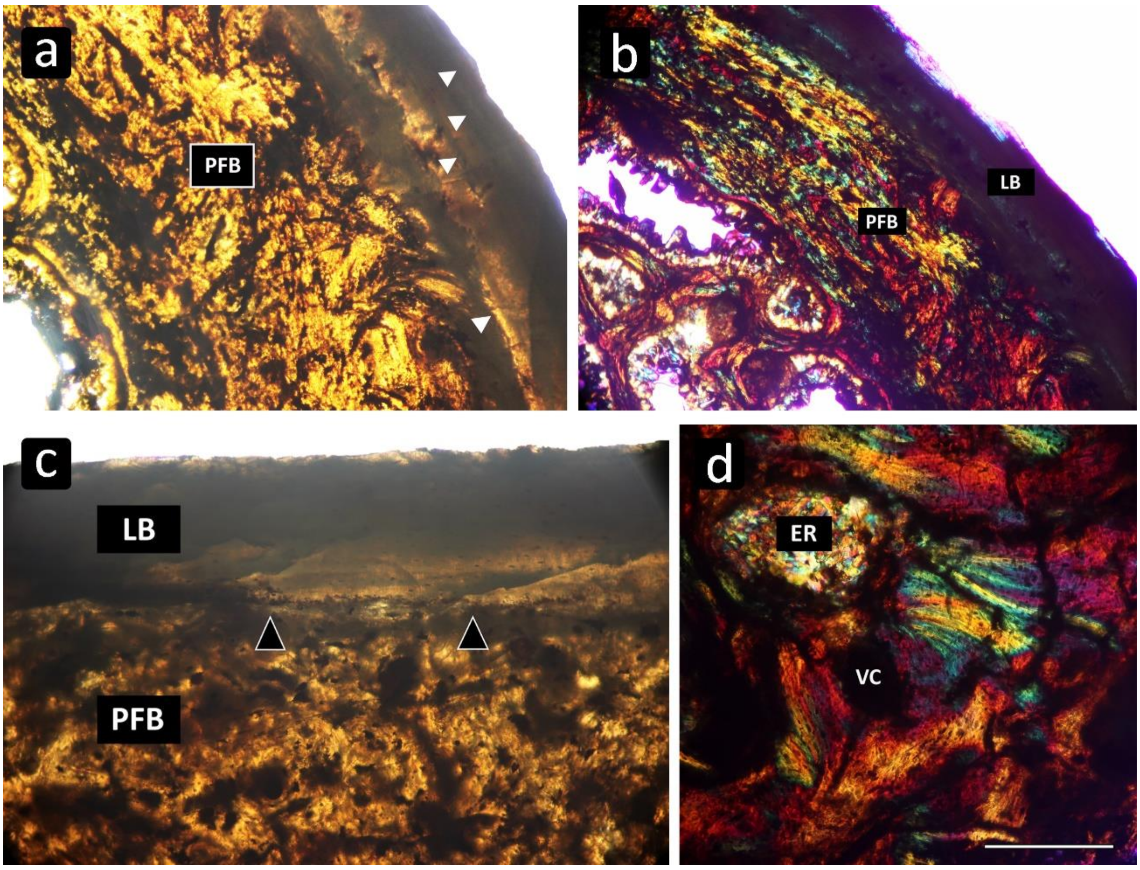

Supplementary figure 15. Transverse section of the femora showing the EFS and the annulus (white arrowheads), and the inner and middle cortex dominated by PFB (a); the same image showing the LB of subperiosteum in polarized light with lambda compensator (b); detail of the femur showing the osteocyte lacunae in the subperiosteum composed of LB and the rest of the cortex delimited by one LAG (black arrowheads) (c); detail of the tibia showing the osteocyte lacunae in polarized light with lambda compensator (d). **Abbreviations**: **ER**, erosion room; **LB**, lamellar bone tissue; **PFB**, parallel-fibered bone tissue; **VC**, vascular canal. Scale bars: 0,75mm (A, B); 0, 35 mm (C); 0,40 mm (D).

SUPPLEMENTARY INFORMATION 4. BODY MASS ESTIMATION

Body mass is a widely used measure in fossils, as it has been related to a wide variety of important biological and ecological factors[32]. However, fossil remains have limitations due to conservation bias, which makes body mass estimates for many taxa not comparable with each other.

Recent works inferred body size based on the combined results of different regression formulas based on different calculations. In this sense, authors choose the results of all of them, and calculate a mean value with the aim to give obtain an approximate body mass number[33,34].

Very few contributions have provided estimates of the body mass of South American Mesozoic mammals[35], possibly because most are known from very fragmentary remains, and most taxa lack associated postcranial remains.

Based on Mesozoic mammals only known by isolated teeth, body mass was calculated based on regression formula grounded on the size of m1 and M1[36-39], as employed by previous authors[35,39-41]. SI2 summarizes the measurements of Cretaceous South American mammals as provided by the indicated bibliographic sources, and the regressions used for body mass estimations are also provided. These estimates were taken from measurements provided by the bibliography. Valid taxa follow recent review by Rougier et al.[42].

In the case of Meridiolestida, most calculations are based on tooth size. For *Groebertherium stipanicici* Bonaparte[43], the measurements are based on a left upper molariform (holotype, MACN-RN 13). In the case of *Leonardus cuspidatus* Bonaparte[44], the dental arrangement proposed by Rougier et al.[42] is followed. In this way, the measurements taken from the Mf1 in Rougier et al.[42] correspond to the Mf2 of Chornogubsky[45] (Table 1); mf1 size is taken from specimen MACN-RN 06.

Size of *Mesungulatum houssayi* Bonaparte[46], and *Orretherium tzen* Martinelli et al.[47] was based on size of m1 published by Martinelli et al.[47]. *Reigitherium bunodontum* Bonaparte[44] size was calculated based on tooth size of specimens MPEF 2020, 2338, 2317, 2393, 2394[48,49]. *Casamiquelia rionegrina* Bonaparte[44] and *Paraungulatum rectangularis* Bonaparte[50] measurements were based on Bonaparte[50]. *Mesungulatum lamarquensis* Rougier et al.[51] and *Trapalcotherium matuastensis* Rougier et al.[51] size was calculated based on measurements published by Rougier et al.[51].

In the case of the gondwanatherians *Sudamerica ameghinoi* Scillato Yané and Pascual[52] and *Ferugliotherium windhausseni* Bonaparte[46] the first molariform size calculation carried on by Gurovich[35] is followed, based on 8 and 2 specimens, respectively. The body size of *Gondwanatherium patagonicum* Bonaparte[46] is based on the size of specimen MACN-RN 23, following data provided by Gurovich[35]. The body size of *Magallanodon baikashkenke* Goin et al.[53] was calculated on measurements provided by Goin et al.[53] and Chimento et al.[54].

From *Patagorhynchus pascuali* Chimento et al.[55] only one right m2 is known. The size of other monotremes known from more complete material, and from which size inferences have been made based on comparisons with the present platypus, was taken as a reference. For *Obdurodon insignis,* a lower m2 of 7.35 mm in length has been described[56]. In this sense, the m2 of *Patagorhynchus pascuali* is 5.8 mm long, which is 78.91% of the length of the m2 of *Obdurodon insignis*. If the mass of the latter has been estimated at 2 kgs[57], 78.91% corresponds to ~1.58 kgs. Thus, the body mass of *Patagorhynchus pascuali* is around the average mass of a current platypus (1.25 kgs)[57].

For more complete taxa, regressions based on cranial, mandibular and dental measurements were taken[40,58-62], based on published sources. These include the giant eutriconodont *Repenomamus giganticus*, the stem therian *Vincelestes neuquenianus* Bonaparte[46], the gondwanatherians *Vintana sertichi* Krause et al.[63] and *Adalatherium hui* Krause et al.[34], and

the meridiolestidans *Coloniatherium cilinskii* Rougier et al.[64], *Cronopio dentiacutus* Rougier et al.[65], and an indeterminate dryolestoid jaw from northern Patagonia[66].

In the case of the mesungulatid meridiolestidan *Coloniatherium cilinskii*, its cranial size was calculated through three different ways: 87,5 mm, 148,3 mm and 161,5 mm[64]. However, the latter two are high estimates, and in this sense, the last one surpasses the length of the skull of *Repenomamus giganticus* (160 mm)[67], a taxon that is up to the date, considered as the largest Mesozoic mammal. The mandible of *Coloniatherium cilinskii* is about 80 mm in total length[68,69], which is much smaller than that of *Repenomamus giganticus* (128 mm). Further, tooth size of *Coloniatherium cilinskii* (m1L: 5,7; m1W: 4,85; m1area: 27,65) is much smaller than those of *Repenomamus giganticus* (m1L: 7,5; m1W: 4,7; m1area: 35,25). Further, it is worthy to mention that a skull of 148,3mm long or 161,5 mm long as calculated by Rougier et al.[64] is not congruent with mandibular size, which is 80 mm. A skull that nearly duplicates mandibular size is a character not yet recorded among extant or extinct mammals. In sum, here is followed the estimation of 87,5 mm for total skull length[64].

Some additional taxa from the late Cretaceous of the Southern hemisphere had a very large body size. Of 17 valid species (based of 23 specimens) from the Late Cretaceous of South America, 8 taxa (represented by 12 specimens) resulted in having body size greater than 1 kg (see Supplementary Tables 2,3,4,5,6). The gondwanatherian *Vintana sertichi* from Madagascar was described as the largest mammal from the Southern Hemisphere with a body size of about 8,74 kg, an estimation based on its skull size[61,69]. Based on 3 regressions grounded on skull size[70-72], resulted in a mean value of 8,428 kg, very close to previous estimations. In the case of *Adalatherium hui*, Krause et al.[69] obtained a mean value of 3,077 kg, which is very close to that obtained here (3,228 kg) through a mean of 44 regressions based on tooth, skull and postcranial measurements.

Other taxa that resulted with large body masses are *Coloniatherium cilinskii* with 3.69 kg, and *Magallanodon baikashkenke*, with 6.74 kg.

Patagomaia is known mainly by hindlimb material, and thus, body mass estimation was almost based on femur size, as usual in several works based on extant and extinct mammals[67,73-77]. Particularly, the circumference of the femur has been considered as one of the more valuable means to obtain body size[78], and was considered as a main source to infer body size on fossils[75,79-82]. Femoral measurements used (i.e., APH, HH, MLH, MLD, MLWDia, APLDia, APD, DiaC, MLP, APP; see below) were taken with digital caliper.

For *Patagomaia*, the 14 regressions result in a mean of about 14,155 kg (range 2,77-26,07). This value is greater than any known Mesozoic mammal, including the original estimations for *Repenomamus giganticus* (12-14 kgs)[67]. However, regressions based on skull, jaw, and tooth size carried out here, resulted in a mean of about 6,603 kg, which are much closer to recent estimations[33]. In this sense, the few overlapping measurements between *Patagomaia* and *Repenomamus giganticus* (MLWDia and Cf) support that *Patagomaia* was a much larger taxon (Supplementary Table 1).

Measurements taken on the cast specimen (Unnumbered specimen housed in Tokyo University) of *Repenomamus giganticus* holotype (Institute of Vertebrate Paleontology, Beijing, China, V14155) showed higher values than those of *Patagomaia chainko*. It is important to note that in *Repenomamus giganticus,* both femora have been compressed anteroposteriorly, so that the mediolateral width results wider than the original condition. Due to compression, the anteroposterior dimension of the femoral head and shaft have become so narrow that we do not take measurements on them. Similarly, the tibiae are likely to have been mediolaterally compressed, such that the anteroposterior length of the head is probably somewhat greater than life condition. The acetabulum and other parts of the pelvic girdle are hidden by the femurs or broken, so we could not measure them. Finally, all measurement values listed in ST 1 are the average of values measured three times with a caliper.

Supplementary Table 1. AH, acetabular height; **AL**, acetabular length; **APD**, anteroposterior length of the distal end; **APDia**, anteroposterior lenght of the diaphysis; **APH**, anteroposterior length of the femoral head; **APP**, anteroposterior length of the proximal end; **APPu**, anteroposterior lenght of the pubis; **FC**, Femoral shaft cincumference; **HH**, height of the femoral head; **IH**, iliac height; **IsH**, isquiatic height; **MLD**, mediolateral width of the distal end; **MLH**, mediolateral width of the femoral head; **MLP**, Mediolateral width of the proximal end; **MLDia**, mediolateral width of the diaphysis; **MLP**, mediolateral width of the proximal end. Measurenments in mm.

	P. chainko	*R. giganticus*	*R. robustus*	*A. hui*
Pelvic girdle				
AL	16,54			
AH	16,79			
IH	19,22			
IsH	13,3			
APPu	18,4			
Femur				
APP	16,32			
MLP	31,4	36,89		
MLH	16,8			15,8
APH	15,5			
HH	14,4			20,1
MLDia	15,5	16,75	8-9	9,6
APDia	11,6			9,9
FC	42	31	26-27	31
MLD	23,92			
APD	17,97	28,17		
Tibia				
MLP	19,15			
APP	16,32	24,66		

Supplementary Table 2. Regressions based on tooth measurements. All measurements and BM (body mass) are expressed in grams. Taxa present in the Late Cretaceous of South America are shaded in blue. *For *Adalatherium hui* the second postcanine is considered as the first molar.

	**M1L**	**M1W**	**m1L**	**m1W**	**M1 area**	**m1 area**	F1	BM	F2	BM	F3	BM	F4	BM	F5	BM	F6	BM	F7	BM	F8	BM	F9	BM
Groebertherium stipanici	1.00	1.70			1.70												1.80	6.03	3.14	23.19	2.42	11.24		
Leonardus cuspidatus	1.25	2.50	1.47	2.25	3.125	3.3075	1.92	83.09	2.47	295.04	3.22	25.02	6.38	588.03	4.85	128.11	2.53	12.58	4.54	93.69	3.48	32.41	3.16	23.67
Mesungulatum houssayi			3.90	3.70		14.43	3.15	1410.16	3.16	1432.77	6.46	637.84	7.91	2734.62	7.22	1362.72							6.51	671.68
Mesungulatum lamarquensis	4.05	5.29			21.4245												6.41	608.97	7.25	1412.71	6.83	923.61		
	4.05	5.62			22.76												6.41	608.97	7.47	1758.68	6.93	1026.17		
Reigitherium bunodontum			2.57	1.73		4.4461	2.62	420.36	2.11	128.02	5.07	159.78	5.56	261.05	5.33	205.96							5.08	160.72
			2.31	2.66		6.1446	2.49	308.46	2.70	502.17	4.72	112.15	6.89	986.37	5.85	346.19							4.71	111.49
	2.45	4.51			11.05												4.75	115.94	6.68	792.98	5.68	291.82		
Coloniatherium cilinskii			5.70	4.85		27.65	3.63	4241.79	3.53	3385.33	7.72	2247.58	8.75	6310.97	8.26	3868.84							7.81	2467.74
Peligrotherium tropicalis			7.50	8.70		65.25	3.97	9406.49	4.34	21669.69	8.63	5588.48	10.56	38394.45	9.64	15352.79							8.75	6323.97
Casamiquelia rionegrina	1.10	1.40			1.54												2.11	8.25	2.44	11.48	2.25	9.46		
Orretherium tzen			3.12	3.64		11.36	2.87	737.97	3.13	1360.25	5.72	304.13	7.86	2599.90	6.83	927.84							5.74	312.51
Paraungulatum rectangularis	4.20	2.90			12.18												6.53	686.62	5.08	160.33	5.85	345.73		
Ferugliotherium widhausseny						3.23									4.81	123.33								
Gondwanatherium patagonicum						16.8504									7.47	1747.81								
Sudamerica ameghinoi						18.09875									7.58	1960.25								
Trapalcotherium matuastensis			2.5	2.07		5.1336	2.58	379.05	2.35	226.38	4.96	141.96	6.12	454.47	5.56	259.43							4.96	142.23
Magallanodon baikashkenke	5.3	3.7	8.3	6.31	19.61	52.6254	4.11	12800.41	3.89	7810.76	8.98	7949.06	9.56	14231.30	9.29	10871.87	7.30	1479.46	5.96	387.29	6.67	791.80	9.12	9100.87
Treslagosodon shehuensis			1.6	1.4		2.2400									4.23	68.54								
*Adalatherium hui**	5.40	6.80	6.50	3.10	36.72	20.15	3.79	6209.76	2.91	816.69	8.15	3475.56	7.37	1582.93	7.75	2328.88	7.36	1573.59	8.16	3506.10	7.77	2358.50	8.26	3871.55
Repenomamus giganticus	6.40	4.80	7.50	4.70	30.72	35.25	3.97	9406.49	3.49	3063.76	8.63	5588.48	8.65	5727.12	8.65	5714.47	7.92	2756.68	6.90	993.65	7.46	1729.09	8.75	6323.97
Gobiconodon ostromi			4.50	2.20		9.90	3.33	2136.11	2.44	274.71	6.93	1025.60	6.31	548.58	6.61	744.36							7.00	1097.16

Supplementary Table 2. Continue**.**

	M1L	M1W	m1L	m1W	M1 area	m1 area	F10	BM	F11	BM	F12	BM	F13	BM	F14	BM	F15	BM	F16	BM	F17	BM
Groebertherium stipanici	1.00	1.70			1.70						1.78	5.90	3.05	21.14	2.37	10.64						
Leonardus cuspidatus	1.25	2.50	1.47	2.25	3.125	3.3075	6.45	630.53	4.89	133.54	2.51	12.35	4.46	86.79	3.43	30.83	3.32	27.75	6.28	534.62	4.79	120.31
Mesungulatum houssayi			3.90	3.70		14.43	7.97	2903.24	7.29	1458.68							6.34	568.63	7.85	2553.95	7.09	1197.66
Mesungulatum lamarquensis	4.05	5.29			21.4245						6.40	603.87	7.21	1350.49	6.79	890.46						
	4.05	5.62			22.76						6.40	603.87	7.43	1685.52	6.90	989.76						
Reigitherium bunodontum			2.57	1.73		4.4461	5.64	281.39	5.37	215.84							5.05	156.40	5.46	233.99	5.25	190.86
			2.31	2.66		6.1446	6.96	1054.12	5.90	364.92							4.72	112.42	6.81	904.93	5.76	316.17
	2.45	4.51			11.05						4.74	114.45	6.62	753.00	5.63	280.05						
Coloniatherium cilinskii			5.70	4.85		27.65	8.80	6663.93	8.34	4190.01							7.52	1840.41	8.70	5980.80	8.10	3302.17
Peligrotherium tropicalis			7.50	8.70		65.25	10.60	40070.74	9.73	16886.35							8.37	4303.25	10.53	37552.21	9.44	12607.32
Casamiquelia rionegrina	1.10	1.40			1.54						2.09	8.09	2.34	10.38	2.19	8.96						
Orretherium tzen			3.12	3.64		11.36	7.92	2761.12	6.90	988.90							5.65	285.03	7.79	2425.99	6.71	824.28
Paraungulatum rectangularis	4.20	2.90			12.18						6.52	681.09	5.01	149.45	5.81	332.00						
Ferugliotherium widhausseny						3.23			4.86	128.50											4.75	115.94
Gondwanatherium patagonicum						16.8504			7.54	1876.12											7.33	1525.42
Sudamerica ameghinoi						18.09875			7.65	2106.86											7.44	1705.33
Trapalcotherium matuastensis			2.5	2.07		5.1336	6.19	488.13	5.61	272.58							4.94	140.06	6.02	411.34	5.48	238.86
Magallanodon baikashkenke	5.3	3.7	8.3	6.31	19.61	52.6254	9.61	14948.35	9.39	11911.65	7.29	1470.62	5.90	364.71	6.64	762.91	8.70	5977.10	9.52	13679.79	9.11	9014.51
Treslagosodon shehuensis			1.6	1.4		2.2400			4.26	70.95											4.18	65.50
*Adalatherium hui**	5.40	6.80	6.50	3.10	36.72	20.15	7.43	1686.49	7.83	2507.90	7.36	1564.46	8.13	3387.24	7.73	2282.45	7.92	2763.43	7.29	1464.29	7.61	2016.26
Repenomamus giganticus	6.40	4.80	7.50	4.70	30.72	35.25	8.71	6051.23	8.73	6216.00	7.92	2744.87	6.85	946.03	7.42	1671.25	8.37	4303.25	8.60	5418.30	8.48	4824.42
Gobiconodon ostromi			4.50	2.20		9.90	6.38	588.49	6.67	791.38							6.79	885.47	6.21	498.15	6.50	665.38

Supplementary Table 2. Continue.

	M1L	M1W	m1L	m1W	M1 area	m1 area	F18	BM	F19	BM	F20	BM	F21	BM	F22	BM	F23	BM	F24	BM	F25	BM
Groebertherium stipanici	1.00	1.70			1.70		1.83	6.23	3.27	26.27	2.49	12.07	1.80	6.022			3.59	36.07				
Leonardus cuspidatus	1.25	2.50	1.47	2.25	3.125	3.3075	2.56	12.97	4.65	104.17	3.55	34.66			3.67	39.39	4.57	96.75	5.36	211.71	3.995	54.35
Mesungulatum houssayi			3.90	3.70		14.43									6.07	433.39			7.55	1896.88	6.69	801.78
Mesungulatum lamarquensis	4.05	5.29			21.4245		6.42	616.09	7.32	1515.20	6.88	974.42	6.14	463.41			7.69	2190.72				
	4.05	5.62			22.76		6.42	616.09	7.54	1880.81	6.99	1082.15	6.24	514.06			7.79	2416.45				
Reigitherium bunodontum			2.57	1.73		4.4461									4.16	63.75			5.80	328.84	4.54	93.31
			2.31	2.66		6.1446									4.68	107.96			6.28	532.29	5.13	168.52
	2.45	4.51			11.05		4.77	118.24	6.75	857.05	5.73	309.31	5.00	148.96			6.62	749.12				
Coloniatherium cilinskii			5.70	4.85		27.65									7.13	1248.95			8.52	4992.51	7.87	2629.70
Peligrotherium tropicalis			7.50	8.70		65.25									8.53	5055.10			9.79	17925.68	9.44	12627.33
Casamiquelia rionegrina	1.10	1.40			1.54		2.14	8.52	2.57	13.13	2.32	10.17	1.63	5.084			3.43	30.73				
Orretherium tzen			3.12	3.64		11.36									5.68	293.46			7.19	1328.07	6.25	517.64
Paraungulatum rectangularis	4.20	2.90			12.18		6.54	694.24	5.18	177.00	5.90	366.19	5.17	176.03			6.78	877.23				
Ferugliotherium widhausseny						3.23									3.63	37.89			5.32	204.37	3.95	52.05
Gondwanatherium patagonicum						16.8504									6.32	557.85			7.78	2389.36	6.97	1064.36
Sudamerica ameghinoi						18.09875									6.44	626.68			7.89	2657.56	7.10	1212.82
Trapalcotherium matuastensis			2.5	2.07		5.1336									4.39	80.57			6.01	407.32	4.80	121.34
Magallanodon baikashkenke	5.3	3.7	8.3	6.31	19.61	52.6254	7.31	1490.32	6.05	422.57	6.73	835.87	5.99	398.19	8.18	3562.04	7.55	1898.03	9.47	13015.83	9.05	8525.05
Treslagosodon shehuensis			1.6	1.4		2.2400									3.04	20.88			4.78	118.53	3.28	26.67
*Adalatherium hui**	5.40	6.80	6.50	3.10	36.72	20.15	7.37	1584.67	8.22	3715.42	7.82	2478.87	7.06	1166.89	6.62	746.37	8.57	5245.59	8.04	3118.07	7.30	1475.65
Repenomamus giganticus	6.40	4.80	7.50	4.70	30.72	35.25	7.93	2768.56	6.98	1070.73	7.51	1819.61	6.76	859.46	7.53	1855.10	8.28	3928.51	8.88	7168.34	8.32	4099.52
Gobiconodon ostromi			4.50	2.20		9.90									5.46	234.69			6.99	1082.62	6.00	402.81

Supplementary Table 2. Continue**.**

	M1L	M1W	m1L	m1W	M1 area	m1 area	F26	BM	F27	BM	F28	BM	Average	Range	
Groebertherium stipanici	1.00	1.70			1.70								14.98	5.90	36.07
Leonardus cuspidatus	1.25	2.50	1.47	2.25	3.125	3.3075	4.95	141.85	4.72	112.07	5.14	170.35	142.10	12.35	630.53
Mesungulatum houssayi			3.90	3.70		14.43	7.22	1367.03	7.21	1357.15	7.44	1705.82	1440.82	433.39	2903.24
Mesungulatum lamarquensis	4.05	5.29			21.4245								1049.99	463.41	2190.72
	4.05	5.62			22.76								1198.41	514.06	2416.45
Reigitherium bunodontum			2.57	1.73		4.4461	5.41	223.57	5.22	184.93	5.60	270.57	210.55	63.75	420.36
			2.31	2.66		6.1446	5.91	367.73	5.77	319.82	6.11	448.79	415.56	107.96	1054.12
	2.45	4.51			11.05								411.90	114.45	857.05
Coloniatherium cilinskii			5.70	4.85		27.65	8.22	3715.64	8.31	4079.87	8.46	4715.51	3875.40	1248.95	6663.93
Peligrotherium tropicalis			7.50	8.70		65.25	9.54	13920.41	9.77	17461.20	9.80	18065.25	17247.69	4303.25	40070.74
Casamiquelia rionegrina	1.10	1.40			1.54								11.30	5.08	30.73
Orretherium tzen			3.12	3.64		11.36	6.85	945.83	6.81	904.77	7.07	1172.90	1099.45	285.03	2761.12
Paraungulatum rectangularis	4.20	2.90			12.18								422.36	149.45	877.23
Ferugliotherium widhausseny						3.23	4.92	136.77	4.68	107.66	5.10	164.15	118.96	37.89	204.37
Gondwanatherium patagonicum						16.8504	7.46	1735.22	7.48	1764.58	7.68	2173.99	1648.30	557.85	2389.36
Sudamerica ameghinoi						18.09875	7.57	1936.83	7.60	1991.54	7.80	2431.10	1847.66	626.68	2657.56
Trapalcotherium matuastensis			2.5	2.07		5.1336	5.63	278.91	5.46	235.90	5.83	338.80	271.61	80.57	488.13
Magallanodon baikashkenke	5.3	3.7	8.3	6.31	19.61	52.6254	9.21	10000.60	9.40	12133.14	9.47	12905.94	6740.72	364.71	14948.35
Treslagosodon shehuensis			1.6	1.4		2.2400	4.36	77.89	4.06	57.94	4.53	92.60	66.61	20.88	118.53
*Adalatherium hui**	5.40	6.80	6.50	3.10	36.72	20.15	7.73	2284.56	7.78	2388.51	7.96	2875.59	2517.01	746.37	6209.76
Repenomamus giganticus	6.40	4.80	7.50	4.70	30.72	35.25	8.59	5399.56	8.73	6156.44	8.84	6896.00	4125.03	859.46	9406.49
Gobiconodon ostromi			4.50	2.20		9.90	6.64	765.80	6.58	717.13	6.85	946.27	788.51	234.69	2136.11

Supplementary Table 3. Regressions based on skull and mandible measurements. Abbreviations: SL, skull length; ML, mandible length. The asterisk indicates that the measurements were taken from Forasiepi et al.[66].

	**SL**	**ML**	F29	BM	F30	BM	F31	BM	F32	BM	Average	Range	
Cronopio dentiacutus	27.00		1.66	45.77	1.44	27.38	-1.11	77.65			50.27	27.38	77.65
Coloniatherium cilinskii	87.50	80	3.44	2765.18	3.32	2072.95	0.49	3079.54	7.33	1525.38	2360.76	1525.38	3079.54
Edentolous mandible (Coniacian dryolestoid)*		34.30							4.82	123.97	123.97	123.97	123.97
Vincelestes neuquenianus	70.60	64.20	3.12	1308.07	2.97	941.03	0.20	1573.11	6.68	796.62	1154.71	796.62	1573.11
Vintana sertichii	124.10		3.97	9355.69	3.88	7500.33	0.96	9194.02			8683.35	7500.33	9355.69
Adalatherium hui	84.00	65.00	3.38	2398.20	3.25	1783.81	0.43	2710.16	6.72	826.44	1929.65	826.44	2710.16
Repenomamus giganticus	160.00	128	4.36	22697.11	4.28	19105.64	1.31	20365.52	8.73	6174.42	17085.67	6174.42	22697.11
Yubaatar zhongyuanensis	70.00	56.60	3.10	1269.70	2.96	911.93	0.19	1531.64	6.31	548.10	1065.34	548.10	1531.64
Gobiconodon ostromi		78.00							7.26	1419.71	1419.71	1419.71	1419.71

Supplementary Table 4. Regressions estimated on femoral measurements. Abbreviations: APH, anteroposterior length of femoral head; MLP, mediolateral width of the proximal end; MLH, mediolateral width of the femoral head; MLD, mediolateral width of the distal end; MLDia, mediolateral width of the diaphysis; APDia, anteroposterior length of the diaphysis; APD, anteroposterior length of the distal end; Fc, femoral circumference; FL, femoral total length.

	APH	MLP	MLH	MLD	MLDia	APDia	APD	Fc	FL	F33	BM	F34	BM	F35	BM	F36	BM	F37	BM	F38	BM	F39	BM
Vincelestes neuquenianus									55.00			-0.16	685.76	2.87	737.74	2.83	680.72						
Patagomaia chainko	15.50	31.40	16.80	23.92	15.50	11.60	17.97	42.00		1.32	20776.80							4.14	13873.61	4.17	14844.40	1.21	16167.13
Adalatherium hui			15.80	20.10	9.60	9.90		31.00	90.80	0.74	5490.77	0.44	2760.32	3.52	3307.89	3.41	2542.82	3.76	5754.65	3.78	6049.45	0.84	6950.01
Repenomamus giganticus					12.5			39	95	1.06	11430.73	0.50	3129.74	3.58	3787.27	3.46	2863.78	4.05	11192.52	4.08	11924.16	1.12	13157.08
Gobiconodon ostromi					7	7			69.00	0.36	2283.48	0.11	1287.50	3.16	1454.37	3.09	1235.57						

	APH	MLP	MLH	MLD	MLDia	APDia	APD	Fc	FL	F40	BM	F41	BM	F42	BM	F43	BM	F44	BM	F45	BM	F46	BM
Vincelestes neuquenianus									55.00														
Patagomaia chainko	15.50	31.40	16.80	23.92	15.50	11.60	17.97	42.00		1.07	11759.44	1.36	23041.56	0.98	9563.49	1.05	11300.67	1.00	10096	1.15	14022.81	0.90	8022.73
Adalatherium hui			15.80	20.10	9.60	9.90		31.00	90.80	0.88	7565.76	0.80	6263.19	0.79	6236.67					1.1	12000.29	0.70	5034.53
Repenomamus giganticus					12.5			39	95			1.11	12838.03										
Gobiconodon ostromi					7	7			69.00	0.46	2883.42	0.42	2653.52										

	APH	MLP	MLH	MLD	MLDia	APDia	APD	Fc	FL	F47	BM	F48	BM	F49	BM	Average	Range	
Vincelestes neuquenianus									55.00							701.41		
Patagomaia chainko	15.50	31.40	16.80	23.92	15.50	11.60	17.97	42.00		1.42	26067.06	1.20	15869.10	0.44	2767.80	14155.17	2767.80	26067.06
Adalatherium hui			15.80	20.10	9.60	9.90		31.00	90.80	0.86	7292.21	1.01	10331.90			6255.75	2542.82	12000.29
Repenomamus giganticus					12.5			39	95	1.17	14712.41					9448.41	2863.78	14712.41
Gobiconodon ostromi					7	7			69.00	0.50	3148.59	0.61	4041.34			2373.47	1235.57	4041.34

Supplementary Table 5. Average BM regressions for taxa with dental, cranial, mandibular, and postcranial measurements.

	Dentition																						
Vincelestes neuquenianus																							
Coloniatherium clinskii	4241.79	3385.33	2247.58	6310.97	3868.84				2467.74	6663.93	4190.01				1840.41	5980.80	3302.17					1248.95	
Adalatherium hui	6209.76	816.69	3475.56	1582.93	2328.88	1573.59	3506.10	2358.50	3871.55	1686.49	2507.90	1564.46	3387.24	2282.45	2763.43	1464.29	2016.26	1584.67	3715.42	2478.87	1166.89	746.37	5245.59
Repenomamus giganticus	9406.49	3063.76	5588.48	5727.12	5714.47	2756.68	993.65	1729.09	6323.97	6051.23	6216.00	2744.87	946.03	1671.25	4303.25	5418.30	4824.42	2768.56	1070.73	1819.61	859.46	1855.10	3928.51
Gobiconodon ostromi	2136.11	274.71	1025.60	548.58	744.36				1097.16	588.49	791.38				885.47	498.15	665.38					234.69	

	Dentition					Skull and mandible			
Vincelestes neuquenianus						1308.07	941.03	1573.11	796.62
Coloniatherium clinskii	4992.51	2629.70	3715.64	4079.87	4715.51	2765.18	2072.95	3079.54	1525.38
Adalatherium hui	3118.07	1475.65	2284.56	2388.51	2875.59	2398.20	1783.81	2710.16	826.44
Repenomamus giganticus	7168.34	4099.52	5399.56	6156.44	6896.00	22697.11	19105.64	20365.52	6174.42
Gobiconodon ostromi	1082.62	402.81	765.80	717.13	946.27				1419.71

	Postcranium												Mean (grs)	Range	
Vincelestes neuquenianus		685.76	737.74	680.72									960.43	680.72	1573.11
Coloniatherium clinskii													3586.89	1248.95	6663.93
Adalatherium hui	5490.77	2760.32	3307.89	2542.82	5754.65	6049.45	6950.01	7565.76	6263.19	6236.67	7292.21	10331.90	3380.47	746.37	7565.76
Repenomamus giganticus	11430.73	3129.74	3787.27	2863.78	11192.52	11924.16	13157.08		12838.03		14712.41		6558.03	859.46	22697.11
Gobiconodon ostromi	2283.48	1287.50	1454.37	1235.57				2883.42	2653.52		3148.59	4041.34	1300.47	234.69	3148.59

Supplementary Table 6. References of the regressions for body mass estimations.

Reference	Regression	Source
F1, formula 1	Log BM = 2.902*log(m1l)+1.434	Millien & Bovy (2010)[83]
F2, formula 2	Log BM = 3.177*log(m1w)+1.351	
F3, formula 3	Ln BM = 1.941+3.319*ln(m1l)	Gordon (2003)[84]
F4, formula 4	Ln BM = 3.871+3.09*ln(m1w)	
F5, formula 5	Ln BM = 2.933+1.605*ln(m1area)	
F6, formula 6	Ln BM = 1.796+3.3*ln(M1l)	
F7, formula 7	Ln BM = 1.223+3.62*ln(M1w)	
F8, formula 8	Ln BM = 1.496+1.74*ln(M1area)	
F9, formula 9	Ln BM = 1.843+3.429*ln(m1l)	
F10, formula 10	Ln BM = 3.957+3.07*ln(m1w)	
F11, formula 11	Ln BM = 2.953+1.623*ln(m1area)	
F12, formula 12	Ln BM = 1.775+3.309*ln(M1l)	
F13, formula 13	Ln BM = 1.108+3.662*ln(M1w)	
F14, formula 14	Ln BM = 1.438+1.747*ln(M1area)	
F15, formula 15	Ln BM = 2.131+3.095*ln(m1l)	
F16, formula 16	Ln BM = 3.732+3.144*ln(m1w)	
F17, formula 17	Ln BM = 2.924+1.56*ln(m1area)	
F18, formula 18	Ln BM = 1.83+3.284*ln(M1l)	
F19, formula 19	Ln BM = 1.373+3.572*ln(M1w)	
F20, formula 20	Ln BM = 1.571+1.733*ln(M1area)	
F21, formula 21	Ln BM = 1.714*ln(M1area)+0.886	Bloch et al. (1998)[85]
F22, formula 22	Ln BM = 1.628*ln(m1area)+1.726	
F23, formula 23	Ln BM = 1.6206*ln(M1area)+2.7256	Gingerich et al. (1982)[86]
F24, formula 24	Ln BM = 1.4885*ln(m1area)+3.5747	
F25, formula 25	Ln BM = 1.827*ln(m1area)+1.81	Legendre (1986)[87]
F26, formula 26	Ln BM = 1.538*ln(m1area)+3.115	
F27, formula 27	Ln BM = 1.693*ln(m1area)+2.694	Lyson et al. (2019)[88]
F28, formula 28	Ln BM = 1.564*ln(m1area)+3.267	
F29, formula 29	Log BM = 3.488*log(SL)-3.332	Millien & Bovy (2010)[83]
F30, formula 30	Log BM = 3.68*log(SL)-3.83	Thewissen & Gingerich (1989)[89]
F31, formula 31	Log BM = 3.13*log(SL)-5.59	Van Valkenburgh (1990)[90]
F32, formula 32	Ln BM = 2.9677*ln(ML)-5.6712	Foster (2009)[91]
F33, formula 33	Log BM = (logMLDia-log5.2)/0.36	Hu et al. (2005)[92]
F34, formula 34	Log BM = (logFL-log63)/0.36	
F35, formula 35	Log BM = 2.993*log(FL)-2.341	Campione & Evans (2012)[93]
F36, formula 36	Log BM = 2.6288*log(FL)-1.7421	
F37, formula 37	Log BM = 2.8977*log(Fc)-0.5615	
F38, formula 38	Log BM = 2.9559*log(Fc)-0.6266	
F39, formula 39	Log BM = 2.78*log(Fc)-3.304	Christiansen & Harris (2005)[94]
F40, formula 40	Log BM = 2.783*log(APDia)-1.892	
F41, formula 41	Log BM = 2.719*log(MLDia)-1.874	
F42, formula 42	Log BM = 2.457*log(MLD)-2.407	
F43, formula 43	Log BM = 2.575*log(APH)-2.012	Figueirido et al. (2011)[95]
F44, formula 44	Log BM = 2.723*log(MLP)-3.072	
F45, formula 45	Log BM = 2.538*Log(MLH)-1.963	
F46, formula 46	Log BM = 2.678*log(MLD)-2.788	
F47, formula 47	Log BM = 2.659*log(MLDia)-1.749	
F48, formula 48	Log BM = 2.708*log(APDia)-1.682	
F49, formula 49	Log BM = 3.108*log(APD)-3.457	

In addition, comparisons were made and measurements of present-day medium-sized mammals were taken for comparison with measurements taken in *Patagomaia chainko* (see Supplementary Table 7). The measurements of *Patagomaia chainko* are similar to those of some medium-sized mammals with relatively robust bodies and relatively short limbs, such as *Leopardus pardalis, Hystrix cristata, Lycalopex culpaeus*, among others. These species have been documented with body mass ranges of 6.6-18.6 kgs[85], 13-27 kgs[86] and 4-12 kgs[87,88], respectively. This is in agreement with the masses estimated above. Furthermore, when the *Patagomaia* remains were compared directly with these mammals, the size and morphology showed similarities with the aforementioned mammals (see SF10). The acetabulum of *Patagomaia chainko*, for example, is much larger in size than that of *Vincelestes neuquenianus* and *Lycalopex gymnocercus* (see SF15), a small to medium-sized mammal (2-6.8 kgs)[89,90].

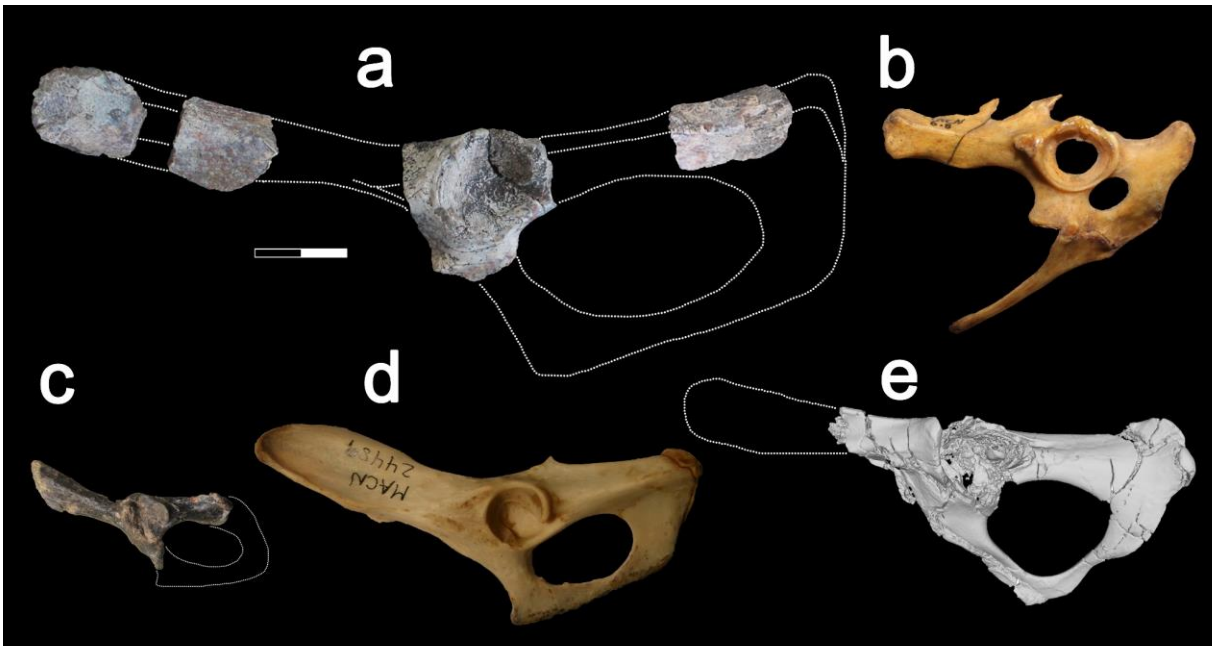

Supplementary Figure 16. Absolute size comparisons of *Patagomaia chainko* (a), *Tachyglossus aculeatus* (b), *Vincelestes neuquenianus* (c), *Lycalopex gymnocercus* (d), and *Adalatherium hui* (e). Scale bar: 20mm.

Supplementary Table 7. Postcranial measurements of selected mammals. Taxa with measurements close to those of *Patagomaia* are indicated in green. Abbreviations: APH, anteroposterior length of femoral head; MLP, mediolateral width of the proximal end; MLH, mediolateral width of the femoral head; MLD, mediolateral width of the distal end; MLDia, mediolateral width of the diaphysis; APDia, anteroposterior length of the diaphysis; APD, anteroposterior length of the distal end; Fc, femoral circunference; FL, femoral total length.

		**Acetabulum**		**Femur**									**Tibia**		Weight	Source
		**AL**	**AH**	**MLP**	**MLH**	**APH**	**PDH**	**MLD**	**APD**	**MLDia**	**APDia**	**FC**	**MLP**	**APP**		
MPM-PV 23365	*Patagomaia chainko*	16.54	16.79	31.4	16.8	15.5	14.4	23.92	17.97	15.5	11.6	42	19.15	16.32		
MACN-Ma 30.231	*Chrysocyon brachyurus*	26.32	24.21	48.23	21.96	23.83	21.91	39	43	18.49	17.08	49	43.84	43.78	20-30	96
MACN-Ma 43.67	*Arctictis binturong*	18.78	21.04	35.7	18.4	17.51	17.82	31.72	26.7	13.84	13.27	46	29.66	23.23	16.4-23	97
MACN-Ma 5.36	*Meles meles*	14.95	14.56	31.03	13.04	13.78	13.28	23.72	21.76	12.41	8.83	38	24.44	22.56	6.6-16.7	98
MACN-Ma 47.218	*Lontra longicaudis*	12.48	11.25	20.01	8.5	9.58	9.52	18.53	17.27	7.3	7.56	28	19.01	14.35	5.0-15.0	99
MACN-Ma 27.964	*Nasua nasua*	13.59	13.24	28.21	12.53	12.89	12.23	22.46	19.5	11.1	9.59	30	24.44	21.22	1.5-6.5	100
MACN-Ma 29.285	*Procyon cancrivorus*	17.29	17.53	33.61	16.16	15.93	15.37	29.61	22.1	15.66	10.42	39	29.9	21.43	4.0-10.1	101,102
MACN-Ma 25.102	*Proteles cristatus*	14.65	16.72	28.86						11.55	10.14	34	25.52	25.99	8.0-14.0	103
MACN-Ma 26.078	*Leopardus pardalis*	15.59	15.47	29.22	13.68	14.92	14.45	28	25.03	14.48	13.68	41	28.78	25.37		
MACN-Ma 27.129	*Leopardus pardalis*	16.26	16.91	33.91	14.76	15.92	15.46	28.49	25.82			41	30.57	26.55		
MACN-Ma 27.087	*Leopardus pardalis*	14.62	15.08	28.32	12.39	13.57	12.49	25.38	24.14	12.89	11.1	37	27.43	22.04	6.6-18.6	104
MACN-Ma 30.365	*Leopardus pardalis*	12.14	11.89	25.73	10.52	11.62	11.38	21.15	20.12	11.47	9.69	32	22.25	19.41		
MACN-Ma 27.888	*Leopardus pardalis*	16.4	16.67	33.64	13.56	15.48	15.07	27.73	25.76	27.77	26.41	40	31.24	22.61		
MACN-Ma 20.821	*Lontra provocax*	15.64	13.03	27.29	14.91	13.74	13.86	23.95	21.57	12.5	8.97	35	24.33	18.7	5.0-10.0	105
MACN-Ma 23.573	*Procyon lotor*	14.67	14.47	28.57	15.11	15.15	14.43	26.09	17.83	14.57	10.88	36	25.27	20.14	2.2-12	106
MACN-Ma 22.5	*Cuniculus paca*	12.3	10.93	25.7	9.91	11.66	11.44	23.85		12.01	9.93	37	23.5	20.05	5.0-13.0	107
MACN-Ma 5.51	*Hystrix cristata*	16.3	15.98	34.86	11.26	15.26	15.37	29.55		15.64	12.27	42	27.67	22.4	13-27	108
MACN-Ma 21.983	*Lagostomus maximus*	11.86	11.45	26.8	10.77	12.1	11.87	24.33	21.42	13.65	9.6	39	25.26	23.55	3.5-8.8	109
MACN-Ma 30.703	*Cerdocyon thous*	12.07	11.85	24.96	12.38	11.77	11.13	21.25	19.58	9.53	8.39	30	22.52	22.63		
MACN-Ma 28.175	*Cerdocyon thous*	11.76	10.93	23	11.37	11.56	10.88	20.69	19.05	10.62	9.71	35	22.04	20.81	5.0-7.0	110
MACN-Ma 30.696	*Cerdocyon thous*	12.92	12.28	25.82	12.22	12.23	11.66	21.94	22.9	11.22	10.45	34	23.06	23.66		
MACN-Ma 47.219	*Pudu puda*	14.45	14.49	28.37	15.92	14.48	13.37	26.15	27.29	10.92	12.21	39	28.08	23.68	5.8-12	111
MACN-Ma 27.592	*Pudu puda*	15.74	15.22	29.33	13.63	15.14	13.62	28.76	29.68	11.45	12.11	38	30.89	27.58		
MACN-Ma 27.164	*Lycalopex culpaeus*	15.66	14.45	30.32	14.05	14.63	14.07	26.53	26.87	13.88	11.39	38	28	26.16		
MACN-Ma 33.69	*Lycalopex culpaeus*	15.2	13.57	30.05	11.54	13.11	12.58	26.49	25.74	12.07	10.81	35	28.49	26.93	4.0-12.0	112,113
MACN-Ma 34.317	*Lycalopex culpaeus*	13.54	13.07	29.01	12.36	12.59	12	24.01	22.72	11.62	9.42	34	26.3	23.89		
MACN-Ma 25.191	*Macaca fuscata*	18.71	18.45	33.75	13.7	15.74	14.46	28.25	23.28	14.04	13.26	45	28.79	22.85	7.6-18.4	114
MACN-Ma 30.367	*Erythrocebus patas*	15.8	13.91	30.72	14.64	14.33	12.72	25.37	21.16	13.63	12.37	36	27.88	21.39	4.0-13.0	115
MACN-Ma 4.125	*Bradypus sp.*	16.57	13.91	23.46	15.22	15.86	13.91	20.52	13.24	14.43	7.53	34	21.06	16.79	2.25-10.1	116
MACN-Ma 10.8	*Tamandua tetradactyla*	17.3	14.68	24.99	16.29	16.45	14.81	22.3	18.98	17.29	9.33	37	22.9	17.19		
MACN-Ma 33.255	*Tamandua tetradactyla*	17.12	15.44	27.67	16.77	16.58	14.92	22.56	18.49	17.51	10.09	41	24.71	16.15	3.8-7	117
MACN-Ma 25.911	*Tamandua tetradactyla*			28.84	17.15	17.39	16.6	24.72	21.8	20.69	8.63	43	26.83	19.64		
LACEV S/N	*Myocastor coypus*	11.1	10.7	25.5	10.6	12	11.9	23	21.4	8.8	8.2	34.9	22.1	18.8	1.9-6.2	118

SUPPLEMENTARY INFORMATION 5. PHYLOGENETIC ANALYSIS

With the aim to test the phylogenetic position of *Patagomaia chainko* among mammals, we conducted three different phylogenetic analyses on the most comprehensive data sources published up to the date, including an analysis focused on allotherians and multituberculates[34], another focused on docodonts, dryolestoids and meridiolestidans[47], and the last one that concentrated on therians[110].

The data were analyzed under equally weighted parsimony using TNT 1.5[111,112]. In both cases, a new technology search of 50,000 replicates of Wagner trees followed by TBR branch-swapping algorithm (holding 100 trees per replication) was performed. The best trees obtained at the end of the replicates were subjected to a final round of TBR branch swapping. Branches with a maximum possible length of zero among any of the recovered most parsimonious trees were collapsed (rule 3)[113,114]. As a measure of branch support, decay indices (=Bremer support) were calculated[115,116], and as a measure of branch stability, a bootstrap resampling analysis[117] was conducted. Both absolute and GC (i.e., difference between the frequency whereby the original group and the most frequent contradictory group are recovered in the pseudoreplications[118]) bootstrap frequencies are reported.

Phylogenetic results from Krause et al.[34] data matrix

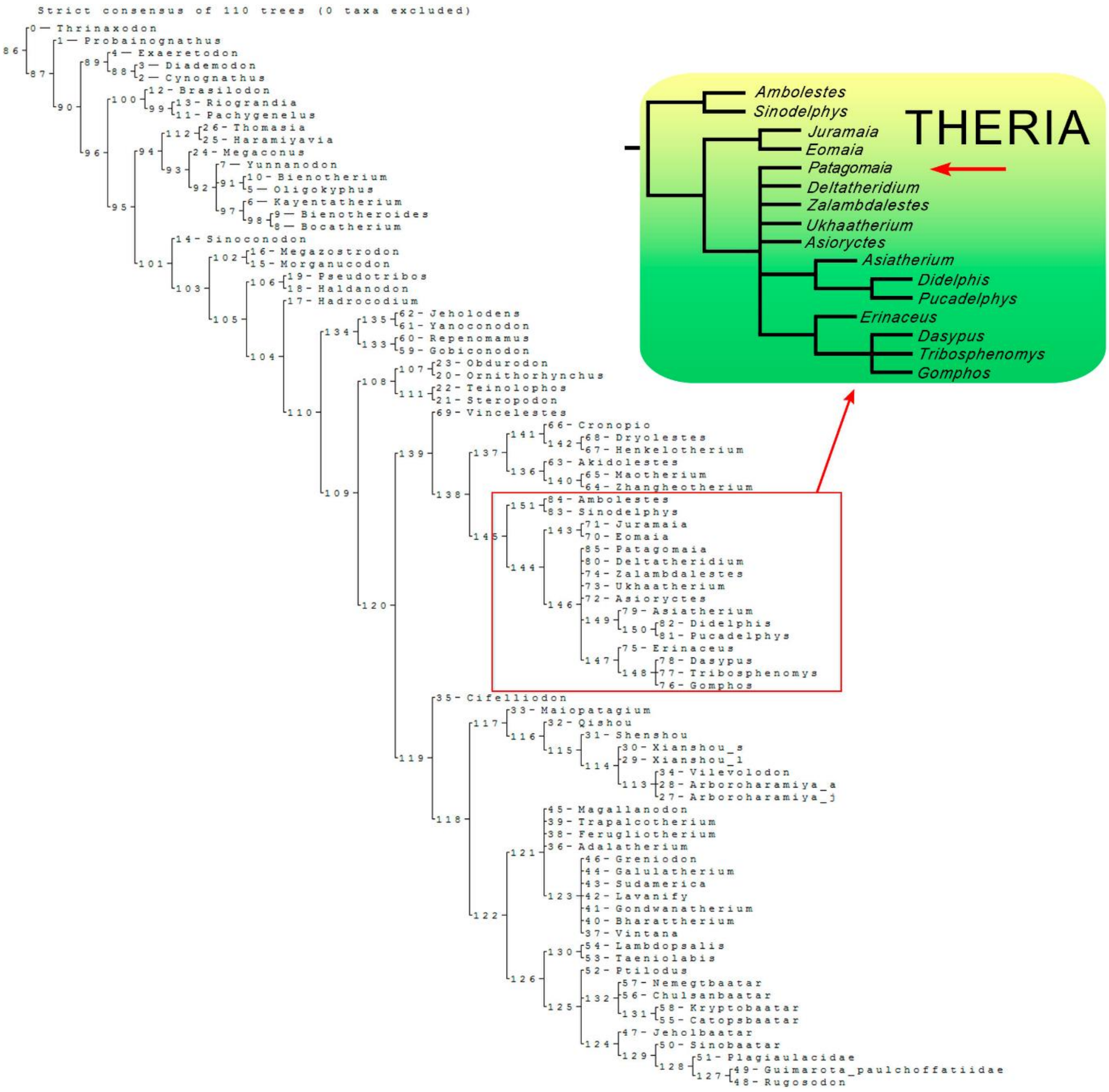

Supplementary Figure 17. Strict consensus tree including *Patagomaia*. Present analysis resulted in 950 Most Parsimonious Trees (MPTs) of a length of 2308 steps, and Ci 0.302 and Ri 0.702. *Patagomaia* were included in the node 146 (Theria).

Codifications of *Patagomaia chainko* in Krause et al.[34] data matrix.

??1101??1111111?0000-
1??1???
???
???
???
???
???

Phylogenetic results from Martinelli et al.[47] data matrix

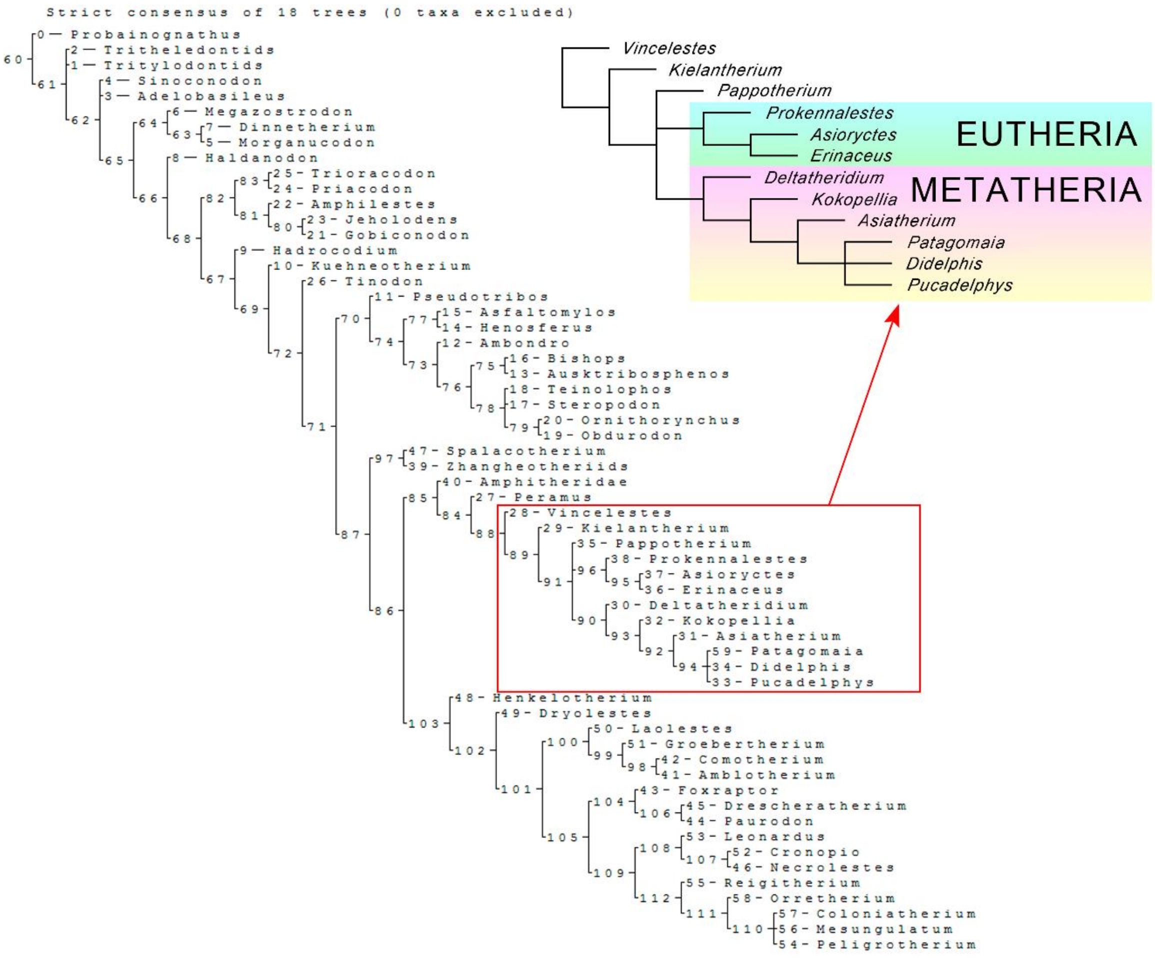

Supplementary Figure 18. Strict consensus tree including *Patagomaia*. Present analysis resulted in 260 Most Parsimonious Trees (MPTs) of a length of 1282 steps, and Ci 0.347 and Ri 0.729. *Patagomaia* were included in the node 94 (Crown Metatheria).

Codifications of *Patagomaia chainko* in Martinelli et al.[47] data matrix.

??
??
??????????????????????????????????1101111111?1????????????????????????????????
??
??????

Phylogenetic results from Zhou et al.[110] data matrix

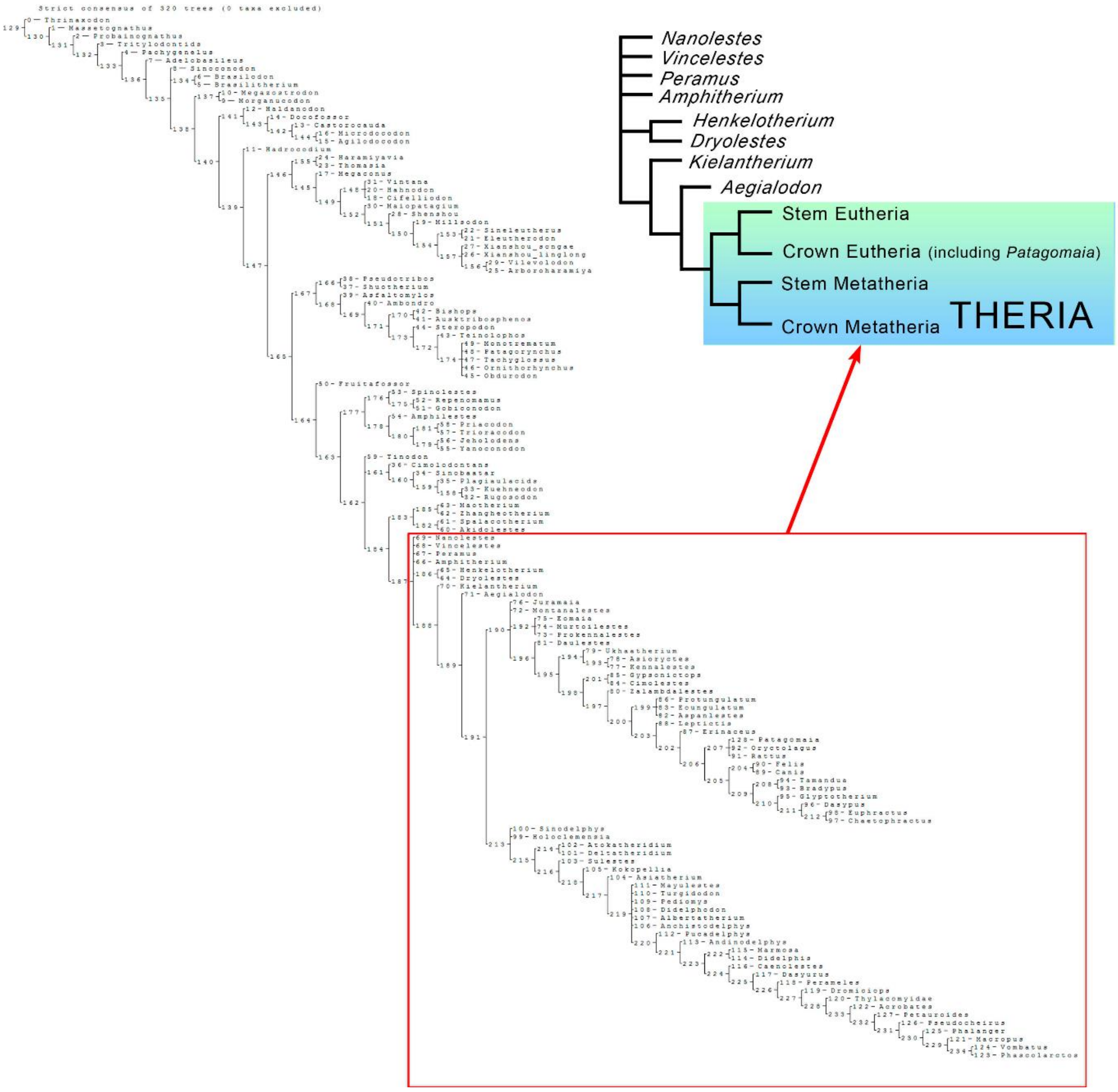

Supplementary Figure 19. Strict consensus tree including *Patagomaia*. Present analysis resulted in 572 Most Parsimonious Trees (MPTs) of a length of 2795 steps, and Ci 0.382 and Ri 0.724. *Patagomaia* were included in the node 196 (Theria).

To this data set, the modifications by Harper et al.[48], Martinelli et al.[47] and Chimento et al.[55] were incorporated. Character 21 was changed from 2 to 1 in *Henkelotherium*, *Dryolestes* and *Laolestes*.

Codifications of *Patagomaia chainko* in Zhou et al.[110] data matrix:

??
??
??
??112?????001111011?11??

???
???
???
???????????????????????????

Character list of the Krause et al.[34] data matrix. The characters scored in *Patagomaia* are indicate in yellow.

1. Cervical vertebrae, Atlas, neural arch, fusion to vertebral "body" (intercentrum) unfused (0); fused (1).
2. Cervical vertebrae, Atlas, neural hemiarches fused unfused (0); fused (1).
3. Cervical vertebrae, Atlas, ribs, presence present (0); absent (1).
4. Cervical vertebrae, Axis, odontoid process (dens) fusion with body of axis unfused (0); fused (1).
5. Cervical vertebrae, Axis, ribs, presence free ribs present (0); ribs fuse to become transverse process (1).
6. Cervical vertebrae, Postaxial vertebrae, ribs, presence free ribs present (0); free ribs absent (1).
7. Dorsal vertebrae, Rib-bearing vertebrae, number 13 or less (0); 14 or more (1).
8. Dorsal vertebrae, Anticlinal vertebra, presence absent (0); present (1).
9. Dorsal vertebrae, Anticlinal vertebra, position anteriorly positioned (in anterior 2/3 of total dorsal vertebrae) (0); posteriorly positioned (in posterior 1/3 of total dorsal vertebrae) (1).
10. Dorsal vertebrae, Lumbar or posterior thoracic ribs, overlapping present (0); absent (1).
11. Dorsal vertebrae, Lumbar ribs, fusion unfused to vertebrae (0); synostosed to vertebrae to form transverse processes (1).
12. Dorsal vertebrae, Lumbar ribs (or transverse processes), orientation posterolaterally or laterally (0); anterolaterally (1).
13. Interclavicle, Presence in adults present (0); absent (1).
14. Interclavicle, Manubrium contact in adults interclavicle abuts manubrium (end-to-end) (0); interclavicle narrowly underlies manubrium (1).
15. Interclavicle, Anterior (= median) process, presence absent (0); present (1).
16. Interclavicle, Posterior process, presence present (0); absent (1).
17. Interclavicle, Lateral process, presence absent (0); present (1).
18. Sternum, Manubrium size relative to succeeding sternebrae large (0); small (1).
19. Clavicle, Acromioclavicular joint, type extensive articulation (0); limited articulation (1).
20. Clavicle, Curvature boomerang-shaped (0); slightly curved (1).
21. Procoracoid, presence in adults present (0); absent or fused (1).
22. Procoracoid foramen, presence present (0); absent (1).
23. Coracoid bone or coracoid process, size large (longer than the maximum dimension of scapular glenoid) (0); small (less than maximum dimension of scapular glenoid) (1).
24. Scapula, Acromion process, presence present (0); absent (1).
25. Scapula, Acromion process, ventral extension not extended anteroventrally beyond the level of glenoid fossa (0); extending anteroventrally beyond the level of glenoid fossa (1).
26. Scapula, Acromion process, shape styliform (0); bulbous (1); plate-like (2).
27. Scapula, Infraspinous fossa, surface contour trough-like (0); flat (1).
28. Scapula, Supraspinous fossa, presence absent (0); present (1).
29. Scapula, Supraspinous fossa, position weakly developed, along anterior part of scapula (0); fully developed, in size to infraspinous fossa (1).
30. Scapula, fossa or process for teres major muscle, presence absent (0); present (1).
31. Scapula, Glenoid fossa, orientation relative to plane or axis of scapular blade nearly parallel to long axis and facing posterolaterally (0); oblique to long axis and facing more posteriorly (1); perpendicular to long axis (2).

32. Scapula, Glenoid fossa, shape and curvature saddle-shaped, oval and elongated (0); uniformly concave and more rounded in outline (1).
33. Scapula, Subscapular (vertebral) surface, contour convex (0); flat (1).
34. Humerus, Head, shape subspherical (0); spherical (1).
35. Humerus, Intertubercular groove (separating deltopectoral crest from lesser tubercle), morphology shallow and broad (0); narrow and deep (1).
36. Humerus, Lesser tubercle, size wider than the greater tubercle (0); narrower than the greater tubercle (1).
37. Humerus, Humeral torsion between proximal and distal ends strong (>30) (0); moderate (30-15) (1); weak (<15) (2).
38. Humerus, Deltopectoral crest, ventral extension or position of deltoid tuberosity not extending beyond midpoint of humeral shaft (0); extending ventrally (distally) past midpoint of shaft (1).
39. Humerus, Ulnar articulation, shape spiral ulnar condyle (0); incomplete trochlea with vestigial ulnar condyle in anterior view (1); trochlea has extending to the anteroventral side (2).
40. Humerus, Radial articulation, shape distinct and rounded condyle separated from the ulnar articulation in the anteroventral view of the humerus (0); radial articulation forms a rounded condyle anteriorly but its posterior surface is nearly cylindrical (1); capitulum, radial articulating structure forms a continuous synovial surface with the ulnar trochlea (2).
41. Humerus, Entepicondyle and ectepicondyle of humerus robust (0); weak (1).
42. Humerus, Supinator ridge, rectangular shelf extending from ectepicondyle, presence absent (0); present (1).
43. Humerus, Supratrochlear foramen, presence absent (0); present (1).
44. Humerus, Supratrochlear foramen, size small (smaller than size of olecranon fossa) (0); large (as large as olecranon fossa) (1).
45. Radius, Styloid process, presence absent (0); present (1).
46. Radius, Styloid process, size small (0); robust (1).
47. Ulna, Olecranon process, length relative to semilunar (=trochlear, sigmoid) notch olecranon shorter than notch (0); olecranon roughly equal to notch (1); olecranon longer than notch (2).
48. Ulna, Olecranon process, posterior inflection, presence absent (0); present (1).
49. Ulna, Coronoid process, presence absent (0); present (1).
50. Ulna, Styloid process, presence absent (0); present (1).
51. Scaphoid, enlargement (including distomedial projection), presence absent (scaphoid < 150% of lunate) (0); present (scaphoid twice size of lunate) (1).
52. Triquetrum, size nearly twice size of lunate (0); subequal to or smaller than lunate (1).
53. Trapezium, morphology elongate to cuboidal (0); bean-shaped or fusiform (1).
54. Trapezium, size larger than or equal to trapezoid (0); smaller than trapezoid (1).
55. Hamate (unciform), shape anteroposteriorly compressed (wider than longer in dorsal view) (0); mediolaterally compressed (longer than wide in dorsal view) (1).
56. Hamate (unciform), size smaller or equal in size to triquetrum (0); much larger than triquetrum (1).
57. Pelvis, Acetabulum, dorsal margin emarginated (0); with complete rim (1).
    Patagomaia: 1, with complete rim.
58. Pelvis, Acetablum, internal sutures of ilium, ischium, and pubis in adults: unfused (0); fused (1).
    Patagomaia: 1, fused.
59. Pelvis, Ischiatic tuberosity, size: small or absent (0); hypertrophied (1).
    Patagomaia: 0, absent.
60. Pelvis, Ischial dorsal margin, shape: concave (0); straight (1).
    Patagomaia: 1, straight.

61. Pelvis, Obturator foramen, size: small, smaller or equal in size to acetabulum (0); intermediate, larger than acetabulum but less than twice size (1); large, more than twice size of acetabulum (2).
62. Pelvis, Epipubic bone, presence: present (0); absent (1).
63. Femur, Proximal end, morphology: neck absent, head oriented dorsally (0); neck present, head inflected medially (1).
    Patagomaia: 1, neck present, head inflected medially.
64. Femur, Neck, degree of development: incipient and short (0); distinct and long (1).
    Patagomaia: 1, distinct
65. Femur, Fovea for acetabular ligament on femoral head, presence absent (0); present (1).
    Patagomaia: 1, present.
66. Femur, Greater trochanter, orientation: directed dorsolaterally (0); directed dorsally (1).
    Patagomaia: 1, directed dorsally.
67. Femur, Greater trochanter, proximal extent: longer than femoral head (0); equal to femoral head (1); shorter than femoral head (2).
    Patagomaia: 1, equal to femoral head.
68. Femur, Lesser trochanter, position: on medial side of shaft (0); on ventromedial or ventral side of shaft (1).
    Patagomaia: 1, ventromedial.
69. Femur, Lesser trochanter, size large, prominent protuberance (0); small, slightly elevated eminence (1).
    Patagomaia: 1, small.
70. Femur, Third trochanter, presence absent (0); present (1).
71. Femur, Subtrochanteric fossa, presence: absent (0); present (1).
    Patagomaia: 0, absent.
72. Femur, Subtrochanteric tubercle, presence: absent (0); present (1).
    Patagomaia: 0, absent.
73. Femur, Posttrochanteric fossa, presence: absent (0); present (1).
    Patagomaia: 0, absent.
74. Femur, Patellar groove, presence: absent (0); present (1).
    Patagomaia: 0, absent.
75. Femur, Patellar groove, mediolateral contour: flat (0); concave (1).
    Patagomaia: -, non-applicable.
76. Tibia, Proximolateral tubercle or tuberosity, size: large and hook-like (0); indistinct (1).
    Patagomaia: 1, indistinct.
77. Tibia, Medial malleolus, presence: weak (0); distinct (1).
78. Tibia, Distal tibial articulation, lateral and medial tibioastragalar facet differentiated: absent (0); present (1).
79. Fibula, Contact with femur, presence: present (0); absent (1).
    Patagomaia: 1, absent.
80. Fibula, lateral malleolus on distal end, presence: weak or absent (0); distinct (1).
81. Fibula, Contact with calcaneus, extent: extensive contact (0); reduced (1); absent (2).
82. Parafibular process or bone, presence: absent (0); present (1).
83. Parafibula, fusion with fibula: unfused, separate bone (0); fused with proximal fibula into parafibular process (1).
84. Astragalus, superposition over calcaneus, extent slight or absent (0); astragalus partially superposed over calcaneus (1); astragalus completely superposed dorsally over calcaneus (2).
85. Astragalus, Proximal trochlea, presence absent (0); present (1).
86. Astragalus, Medial tibial crest (= ridge between medial and lateral tibioastragalar facet), presence absent (0); present (1).

87. Astragalus, Neck, development absent (0); weakly developed (1); well developed (2).
88. Astragalus, Astragalonavicular facet, shape flat or concave (0); convex (1); trochlea (2); crest-in-groove (3).
89. Astragalus, Astragalonavicular facet, medial extent restricted (0); extend onto medial side of astragalar head (1).
90. Astragalus, Astragalonavicular facet, lateral extent restricted (0); extends onto lateral side of astragalar head (1).
91. Calcaneus, Tuber calcanei, size short (less than 40% of calcaneal length) (0); long (greater than 40% of calcaneal length) (1).
92. Calcaneus, Tuber calcanei, terminal swelling, presence absent (0); present (1).
93. Calcaneus, Tuber calcanei, ventral curvature present (0); absent (1).
94. Calcaneus, Peroneal process, presence present (0); reduced or absent (lateral edge of calcaneus straight) (1).
95. Calcaneus, Peroneal process, shape greatly expanded peroneal shelf (extending from cuboidal facet to tuber) (0); small peroneal shelf(demarcated from the cuboidal facet of the calcaneus) (1); knob-like peroneal process (2).
96. Calcaneus, Peroneal groove, presence absent (0); present (1).
97. Calcaneus, Peroneal groove, size weak (0); deep and distinct (1).
98. Calcaneus, Cuboid articular facet, orientation ventrally (0); distally (=anteriorly) (1); ventromedially (2).
99. Calcaneus, Sustentacular facet, orientation relative to horizontal plane nearly vertical (0); oblique (<70) to nearly horizontal (1).
100. Calcaneus, Sustentaculum tali, outline in ventral view indistinct (0); medially directed shelf, with rounded outline (1); protruding triangle, posteromedially directed (2).
101. Calcaneus, Calcaneo-astragal facet, position anterior to calcaneo-fibular facet (0); medial to calcaneo-fibular facet (1).
102. Navicular, transverse width compared to cuboid narrower or subequal to cuboid (0); wider than cuboid (1).
103. Entocuneiform, Distal facet (for metatarsal I), saddle-shaped absent (0); present (1).
104. Metatarsal III, alignment with calcaneus metatarsal III aligned with (or parallel to) long axis of calcaneus (0); metatarsal III oriented obliquely to long axis of calcaneus (1).
105. Metatarsals III, relative length compared proximal pedal phalanx (PPP) III PPP III shorter or equal in length to Mt III (0); PPP III longer than Mt III (1).
106. Metatarsal V, alignment with cuboid metatarsal V offset from cuboid (0); metatarsal V aligned with cuboid (1).
107. Metatarsal V, contact with peroneal process, presence absent (0); present (1).
108. Metatarsal V, Ventrolateral tubercle, presence absent (0); present (1).
109. Sesamoid bones in pedal flexor tendons, presence absent (0); present (1).
110. Sesamoid bones in pedal flexor tendons, pairing unpaired (0); paired (1).
111. Os calcaris, presence absent (0); present (1).
112. Snout, shape in dorsal view incurved anterior to zygomatic arches (0); not incurved anterior to zygomatic arches, trapezoid shaped (1).
113. Snout, length (boundary at anterior orbital margin) <50% of total skull length (0); greater than or equal to 50% of skull length (1).
114. Cranial size, width:length ratio less than 0.8 (0); greater than or equal to 0.8 (1).
115. Cranium, Angle between floor of nasal cavity and nasopharyngeal canal, degree greater than or equal to 165" (0); less than 165" (1).
116. Premaxilla, Internarial process complete bar (connected to nasal) (0); incomplete bar (ventral process present but not connected to nasal) (1); absent (2).
117. Premaxilla, Facial process, contact with nasal, presence absent (0); present (1).
118. Premaxilla, Facial process, contact with lacrimal, presence absent (0); present (1).

119. Septomaxilla, presence present (0); absent (1).
120. Septomaxilla, Intranarial process, presence present (0); absent (1).
121. Septomaxilla, Septomaxillary canal, presence present (0); absent (1).
122. Septomaxilla, Septomaxillary foramen, presence present (0); absent (1).
123. Septomaxilla, Facial process, contact with maxilla, presence present (0); absent (1).
124. Nasal, Nasal overhang (nasal extending more anteriorly than lateral margin of external nasal aperture), presence absent (0); present (1).
125. Nasal, Posterior width broader than width at mid-length (0); narrower or equal to width at mid-length (1).
126. Nasal, Suture with frontal, morphology midline contact anterior to lateral contact (0); midline contact posterior to lateral contact (1); midline contact at same approximate anteroposterior level as lateral contact (2).
127. Nasal, Nasal-lacrimal contact vs. maxilla-frontal contact naso-lacrimal contact (0); maxilla-frontal contact (1).
128. Nasal, Foramina on dorsal surface, presence absent (0); present (1).
129. Nasal, Foramina on dorsal surface, number of pairs one (0); two (1); more than two (2).
130. Nasal, Foramina on dorsal surface, size all small (typical of nutrient foramina) (0); most large (larger than typical nutrient foramina) (1).
131. Nasal, Internasal vacuity, presence absent (0); present (1).
132. Nasal Cavity, Ossified cribriform plate of ethmoid, presence absent (0); present (1).
133. Nasal Cavity, Posterior excavation of nasal cavity into bony sphenoid complex, presence absent (0); present (1).
134. Nasal Cavity, Posterior excavation of nasal cavity into bony sphenoid complex, connection to nasal cavity confluent with nasal cavity (0); partitioned from nasal cavity (1).
135. Nasal Cavity, Maxilloturbinal supporting ridge, presence absent (0); present (1).
136. Maxilla, Contribution to ventral wall of orbit (subtemporal margin), best seen in ventral view absent, jugal forming subtemporal margin (0); present (1).
137. Infraorbital foramina, number numerous small foramina of similar size (0); two or more (1); one (2).
138. Infraorbital foramina, Opening of primary infraorbital foramen visible in ventral view (Fi I sensu Hahn, 1985; not applicable to taxa with numerous small foramina) opens anteriorly, not visible in ventral view (0); opens ventrally, visible in ventral view (1).
139. Infraorbital foramina, Position of primary infraorbital foramen (Fi 1 sensu Hahn, 1985; not applicable to taxa with numerous small foramina) dorsal to premolar region (0); dorsal to molar region (1).
140. Infraorbital foramina, foramen usually between lacrimal, maxilla and/or jugal (Fi3 sensu Hahn, 1985) presencepresent (0); absent (1).
141. Infraorbital canal, Composition of posterior opening (maxillary foramen) maxilla (0); maxilla, lacrimal (1); maxilla, lacrimal, palatine (2).
142. Lacrimal, Facial process, outline large, a quarter to half of snout length (0); small, on orbital rim (1); excluded from face (2).
143. Lacrimal, Facial process, contact with septomaxilla absent (0); present (1).
144. Lacrimal foramen, position on edge of orbit or on face (0); within orbit (1).
145. Lacrimal foramen, number two (0); one (1).
146. Foramen for ramus lateralis of ethmoid branch of V1 (marked by * in Zeller, 1989), presence absent (0); present (1).
147. Palate, Incisive foramen, shape small, round to oval (0); intermediate, elongate (1); very large within palatal fossae (2).
148. Palate, Incisive foramen, posterior edge formed by maxilla (0); formed by premaxilla (1).

149. Palate, Premaxilla, contact with palatine, presence absent, maxillae contact in midline (0); present, maxillae do not contact in midline (1).
150. Palate, Premaxilla, palatal process, thickenings absent (0); present (1).
151. Palate, Premaxilla, ridge between palate and lateral walls absent (0); present (1).
152. Palate, Bony secondary palate, length relative to tooth row terminating anterior to the posterior end of the tooth row (0); terminating level with the posterior end of the tooth row (1); extending posterior to the end of the tooth row (2).
153. Palatine, length of palatal process compared to total length of bony palate (measured from choana to incisive foramen): 50% of total palate length or less (0); greater50% of total palate length (1).
154. Palate, Palatal vacuities, presence: absent (0); present (1).
155. Palate, Major palatine foramina, presence: present (0); absent (1).
156. Palate, Minor palatine foramen, presence: absent (0); present (1).
157. Palate, Minor palatine foramen, ventral opening composition encircled by the pterygoid (and ectopterygoid if present) in addition to the palatine (0); encircled by the palatine and maxilla (1); encircled by palatine (2).
158. Palate, postpalatine torus, presence absent (0); present (1).
159. Palate, Alisphenoid, medial extent onto alveolar process, presence: absent (0); present (1).
160. Zygomatic arch, Jugal, contribution jugal well-developed, forming most of zygomatic arch (0); jugal reduced or absent (1).
161. Zygomatic arch, Jugal, position (only applicable in reduced jugals) not restricted, forming middle part of zygomatic arch (0); restricted to medial side of zygomatic arch (1); restricted to dorsal side of zygomatic arch (2).
162. Zygomatic arch, Jugal, anterior extent extends onto facial part of maxilla, forms part of anterior orbital rim (0); does not reach facial part of maxilla, excluded from anterior orbital rim (1).
163. Zygomatic arch, Jugal, posterior extent contributes to squamosal glenoid (0); borders on but does not contribute to squamosal glenoid (1); terminates anterior to squamosal glenoid (2).
164. Zygomatic arch, Maximum vertical depth relative to length of skull 10-20% (0); <10% (1); >20% (2).
165. Zygomatic arch, Anterior zygomatic root, location (as marked by its posterior edge) anterior to ultimate premolar (0); at ultimate premolar or at premolar/molar junction (1); at molar level (2); posterior to tooth row (3).
166. Zygomatic arch, Anterior Zygomatic root, direction posterolaterally directed (0); transversely directed (1).
167. Orbit, Palatine, exposure absent (0); present (1).
168. Orbit, Palatine, orbital process, contact with frontal absent (0); present (1).
169. Orbit, Frontal, orbital process, contact with maxilla absent (0); present (1).
170. Orbit, Frontal, contact with alisphenoid, presence present (0); absent (1).
171. Orbit, Frontal, contact with alisphenoid, extent anterodorsal corner of alisphenoid contacts posteroventral corner of frontal (frontal-parietal suture) (0); approximately one-half of dorsal edge of alisphenoid contacts frontal (1).
172. Orbit, Prefrontal bone, presence present (0); absent (1).
173. Orbit, Postorbital bone, presence present (0); absent (1).
174. Orbit, Postorbital process, size absent or very small (0); distinct process (1); large, forming postorbital bar (2).
175. Orbit, Postorbital process, position on frontal (0); on parietal (1); on postorbital (2).
176. Orbit, Supraorbital crest, presence absent (0); present (1).
177. Orbit, Orbital vacuity, development large orbital vacuity present (0); small orbital vacuity present (1); orbital vacuity closed (2).
178. Braincase, Anterior lamina, exposure on the lateral braincase wall, presence present (0); absent (1).

179. Braincase, Anterior lamina, exposure on the lateral braincase wall, dorsoventral size as tall as alisphenoid (0); taller than alisphenoid (1); shorter than alisphenoid (2).
180. Braincase, Foramina for mandibular division of trigeminal nerve, composition between anterior lamina and alisphenoid (0); within anterior lamina (1); largely within alisphenoid (2).
181. Braincase, Foramina for mandibular division of trigeminal nerve, number one (0); two or more (1).
182. Braincase, Orbitotemporal groove/canal (for ramus superior of stapedial artery), position orbitotemporal groove present on lateral side of braincase (0); intramural or endocranial, with anterior opening of orbitotemporal canal present (1); absent (2).
183. Braincase, Ventral ascending canal (for ramus superior of stapedial artery) in temporal region, development open groove (0); intramural (1); endocranial (2); absent (3).
184. Braincase, Ventral ascending canal (for ramus superior of stapedial artery) , orientation vertical (0); oblique (1).
185. Braincase, Posttemporal canal (for arteria and vena diploëtica magna), posterior opening, presence present (0); absent (1).
186. Braincase, Posttemporal canal (for arteria and vena diploëtica magna), posterior opening, position at the junction of the petrosal, squamosal and the tabular (0); between petrosal and squamosal (1); within petrosal (2); between petrosal and tabular (3); within tabular (4).
187. Squamosal, Cranial moiety, external size narrow (0); broad (1).
188. Squamosal, Neck between glenoid fossa and cranial moiety of squamosal, presence absent (0); present (1).
189. Squamosal, Craniomandibular joint (center of articular surface), position posterior or lateral to level of fenestra vestibuli (0); anterior to level of fenestra vestibuli (1).
190. Squamosal, Glenoid fossa for dentary condyle, position principally on braincase (0); principally on zygoma (1).
191. Squamosal, Glenoid fossa for dentary condyle, outline subcircular (0); oval, long axis anteroposterior (1); oval, long axis mediolateral (2).
192. Squamosal, Glenoid fossa for dentary condyle, contour distinctly concave (0); essentially flat (1).
193. Squamosal, Glenoid fossa or dentary condyle, medial margin, compsoition formed entirely by the squamosal (0); with a contribution from the alisphenoid (1).
194. Squamosal, Postglenoid process, presence absent (0); present as a distinctive process (1).
195. Squamosal, Postglenoid region extending posteriorly into postglenoid shelf absent (0); present (1).
196. Squamosal, Entoglenoid process, presence absent or vestigial (0); present (1).
197. Squamosal, Postglenoid foramen, presence: absent (0); present (1).
198. Squamosal, Medial process, directed toward foramen ovale, presence: absent (0); present (1).
199. Entopterygoid crest (= medial pterygoid plate), composition palatine, pterygoid (0); palatine, alisphenoid (1); maxilla, pterygoid (2).
200. Entopterygoid crest (= medial pterygoid plate), Transverse process, presence: present, massive (0); present as hamulus (1); greatly reduced or absent (2).
201. Ectopterygoid process (= lateral pterygoid plate) of alisphenoid, presence: absent (0); present (1).
202. Pterygoid, Pterygopalatine ridge, presence: present (0); absent (1).
203. Pterygoid, Left/right pterygoid contact at midline, presence: present (0); absent (1).
204. Pterygoid, Interpterygoid vacuity, presence: present (0); absent (1).
205. Ectopterygoid bone, presence: present (0); absent (1).

206. Mesocranium, width anterior to basisphenoid width at choanae narrower than width of basisphenoid (0); width at choanae subequal to or broader than width of basisphenoid (1).
207. Mesocranium, Basipharyngeal passage near pterygoid-basisphenoid junction, shape roof of pharynx V-shaped in transverse section, narrowing toward basisphenoid (0); roof of pharynx U-shaped in transverse section (1).
208. Alisphenoid, Quadrate ramus, presence present, forming rod overlapping anterior part of lateral flange (0); present, mostly laminar process in vicinity of foramen ovale (1); absent (2).
209. Alisphenoid, Tympanic process, presence absent or vestigal (0); present (1).
210. Basicranium, Basisphenoid wing on ventral aspect of skull present, overlapping part of or whole cochlear housing (0); absent (1).
211. Basicranium, Prootic and opisthotic fusion absent (0); present (1).
212. Petrosal, Pars cochlearis, relationship to lateral lappet (muscular tubercle) of basioccipital pars cochlearis is entirely covered by basioccipital (0); pars cochlearis partially covered by basioccipital (1); pars cochlearis fully exposed as promontorium (2).
213. Petrosal, Pars cochlearis, promontorium, ventromedial surface, contour flat (0); inflated, convex (1).
214. Petrosal, Pars cochlearis, promontorium, ventral outline and morphology triangular (0); elongate, cylindrical (1); oval-shaped, bulbous (2).
215. Petrosal, Pars cochlearis, cochlear canal, coiling short, uncoiled (0); elongate canal, straight or slightly curved (1); elongate canal, partly coiled >270" (2); coiled at least 360" (3).
216. Petrosal, Internal acoustic meatus, cribriform plate, presence absent (0); present (1).
217. Petrosal, Internal acoustic meatus, depth shallow, depth less than maximum width (0); deep, depth greater than maximum width (1).
218. Petrosal, Pars cochlearis, primary bony lamina, presence absent (0); present (1).
219. Petrosal, Pars cochlearis, primary bony lamina, double lamina, presence (only applicable to taxa with primary bony lamina): absent (0); present (1).
220. Petrosal, Pars cochlearis, secondary bony lamina, presence absent (0); present (1).
221. Petrosal, Pars cochlearis, secondary bony canal (parallel to cochlear ganglion canal), presence: absent (0); present (1).
222. Petrosal, Pars cochlearis, trans-cochlear sinuses (of Panciroli et al., 2018), presence present (0); absent (1).
223. Petrosal, Pars cochlearis, circum-promontorial plexus, presence present (0); absent (1).
224. Petrosal, Pars cochlearis, circum-promontorial plexus openings along abneural aspect of cochlear canal, presence present (0); absent (1).
225. Petrosal, Pars cochlearis, crista interfenestralis, orientation horizontal, extending to base of paroccipital process (0); vertical, delimiting back of promontorium (1).
226. Petrosal, Pars cochlearis, fenestra vestibuli, thickened rim, presence present (0); absent (1).
227. Petrosal, Pars cochlearis, stapedial artery sulcus absent (0); present (1).
228. Petrosal, Pars cochlearis, transpromontorial sulcus for internal carotid artery absent (0); present (1).
229. Petrosal, Pars cochlearis, channel of perilymphatic duct, closure open channel and sulcus (0); channel partially or fully enclosed (1); no indication (2).
230. Petrosal, Pars cochlearis, fenestra cochleae/perilymphatic foramen, separation from jugular foramen within same depression (0); separate (1).
231. Petrosal, Jugular fossa, size small, shallow (0); large, deep (1).
232. Petrosal, Jugular foramen size relative to fenestra cochleae/perilymphatic foramen (applicable only to those taxa with jugular foramen fully separated from fenestra cochleae/perilymphatic foramen): jugular foramen subequal to fenestra

cochleae/perilymphatic foramen (0); jugular foramen larger than fenestra cochleae/perilymphatic foramen (1).

233. Petrosal, Bony floor anterior to tympanic aperture of prootic canal and/or primary facial foramen absent (0); present, extending length of pars cochlearis (1); present, extending half length of pars cochlearis (2).
234. Petrosal, Cavum epiptericum, anteroventral opening large opening (approximately same length as pars cochlearis) (0); small opening (less than length of pars cochlearis) (1); absent (2).
235. Petrosal, Cavum supracochleare for geniculate ganglion, bony flooring, presence absent (0); present (1).
236. Petrosal, Cavum supracochleare for geniculate ganglion confluent with cavum epiptericum (0); separated by at least partial bony wall (1).
237. Petrosal, Prootic canal, tympanic opening, presence present (0); absent (1).
238. Petrosal, Prootic canal, endocranial opening, presence absent (0); present (1).
239. Petrosal, Prootic canal confluent with pterygoparoccipital foramen (foramen for ramus superior; tympanic opening of ventral ascending canal) absent (0); present (1).
240. Petrosal, Pterygoparoccipital foramen (foramen for ramus superior of stapedial artery; tympanic opening of ventral ascending canal), presence present (0); absent (1).
241. Petrosal, Pterygoparoccipital foramen, morphology laterally open notch (0); foramen enclosed by petrosal or squamosal or both (1); absent (2).
242. Petrosal, Pterygoparoccipital foramen, position relative to fenestra vestibuli posterior or lateral to level of fenestra vestibuli (0); anterior to level of fenestra vestibuli (1).
243. Petrosal, Lateral flange, anterior part, presence present (0); absent or vestigial (1).
244. Petrosal, Lateral flange, anterior part, orientation horizontal shelf (0); ventrally directed (1); medially directed, contacting promontorium (2).
245. Petrosal, Lateral flange, posterior part, vascular foramen anterior to pterygoparoccipital foramen, presence present (0); absent (1).
246. Petrosal, Lateral flange, relationship to anterior paroccipital process (crista parotica) widely separated (0); narrowly separated (1); continuous bone formed by petrosal (2).
247. Petrosal, Paroccipital process, presence no ventral projection below level of surrounding structures (0); projecting ventrally below level of surrounding structures (1).
248. Petrosal, Anterior paroccipital region, morphological differentiation anterior paroccipital region indistinct from surrounding structures (0); anterior paroccipital region bulbous, distinctive from surrounding structures (1); anterior paroccipital region with distinct crista parotica (2).
249. Petrosal, Paroccipital process, relationship with squamosal squamosal covers entire paroccipital region (0); no squamosal cover of anterior paroccipital region (1); squamosal covering part of paroccipital region, but not crista parotica (squamosal wall and crista parotica separated by epitympanic recess) (2).
250. Petrosal, Caudal tympanic process, presence absent (0); present (1).
251. Petrosal, Caudal tympanic process of petrosal, shape continuous crest (0); notched (1).
252. Petrosal, Rostral tympanic process of petrosal, presence absent (0); present (1).
253. Petrosal, Epitympanic recess, presence absent (0); present (1).
254. Petrosal, Fossa incudis continuous with epitympanic recess (0); separate from epitympanic recess (1).
255. Petrosal, Epitympanic recess, size subequal to fossa incudis (0); larger than fossa incudis (1).
256. Petrosal, Post-promontorial tympanic recess, presence absent (0); present (1).

257. Petrosal, Tensor tympani fossa, size indistinct or very shallow (0); deep recess on lateral trough/tegmen tympani (1).
258. Petrosal, Fossa for stapedius muscle, presence absent (0); present (1).
259. Petrosal, Hiatus Fallopii on tympanic surface of petrosal (0); at anterior edge of petrosal (1); endocranial (2).
260. Petrosal, Tympanohyal, relationship with pars cochlearis not contacting (0); contacting (1).
261. Petrosal, Tympanohyal, fusion with pars cochlearis (only applicable for taxa with contact) not fused to petrosal (0); fused to petrosal (1).
262. Carotid foramen present (0); absent (1).
263. Carotid foramen, position within basisphenoid (0); at junction of basisphenoid and petrosal (1); through opening of cavum epiptericum. (2).
264. Incus (quadrate), facet for malleus (articular), shape trochlear surface (0); trough or saddle-shaped surface (1); flat surface (2); convex surface (3).
265. Incus (quadrate) neck, separation of dorsal plate and trochlea of quadrate(representing differentiation between crus breve and body of incus) absent (0); present (1).
266. Incus (quadrate), Crus longum (stapedial process), presence absent (0); present (1).
267. Incus (quadrate), Crus breve (dorsal plate), presence broad plate (0); pointed triangle (1); reduced (2).
268. Incus (quadrate), angle of crus breve (dorsal plate) to crus longum (stapedial process): aligned or obtuse (0); perpendicular (1); acute(2).
269. Incus (quadrate), primary suspension on basicranium by squamosal and quadratojugal (0); by squamosal only (1); by petrosal (either by preserved direct contact of incus or by inference from presence of fossa incudis) (2).
270. Quadratojugal notch in squamosal, presence present (0); absent (1).
271. Stapes, shape quadrangular bicrurate (0); columelliform (1); triangular bicrurate (2).
272. Stapes, Stapedial foramen, presence present (0); absent (1).
273. Stapes, Stapedial foramen, size macroperforate, foramen larger than crura (0); microperforate, foramen smaller than crura (1).
274. Stapes, process for insertion of the stapedius muscle (see Meng et al., 2016, 2019), presence absent (0); present (1).
275. Stapes, process for insertion of the stapedius muscle (see Meng et al., 2016, 2019), size large (approaching width of stapes) (0); small (much smaller than width of stapes) (1).
276. Malleus (articular), fusion with gonial (prearticular) not fused (0); at least partially fused (1).
277. Malleus (articular), Neck, presence absent (0); present (1).
278. Malleus (articular), Manubrium, length shorter than combined width of surangular and prearticular anterior to incudo-malleolar joint (0); longer than combined width of surangular and prearticular (1).
279. Malleus (articular), Manubrium, thickness robust (0); gracile (1).
280. Ectotympanic (angular), size/shape plate-like (0); curved, rod-like (1); ring-shaped (2); slightly expanded (fusiform) (3); expanded (4); tube-like (5).
281. Ectotympanic (angular), angle to skull base primarly vertical (0); primarly oblique (1); primarly horizontal (2).
282. Ectotympanic (angular), Anterior process, presence present (0); absent (1).
283. Ectotympanic (angular), Incisura tympanica, position/orientation posteroventral (0); posterior (1); posterodorsal (2); dorsal (3).
284. Frontal, anterior extension posterior to anterior border of orbit (0); anterior to orbit but posterior to anterior tip of lacrimal (1); anterior to lacrimal (2).
285. Frontal-parietal suture, shape in dorsal view V-shaped, apex directed posteriorly (0); U-shaped, convex posteriorly (1); U-shaped, convex anteriorly (2); roughly transverse (3).

286. Parietal, foramen, presence present (0); absent (1).
287. Parietal, contact with nasals, presence absent (0); present (1).
288. Parietal, Sagittal crest, presence/size prominently developed (0); weakly developed (1); absent (2).
289. Parietal, Sagittal crest, pachyostal (thickened on sagittal midline), presence absent (0); present (1).
290. Occiput, Nuchal (=lambdoid) crest, shape crest overhanging the concave or straight supraocciput (0); weak crest with convex dorsal part of the occipital plate (1).
291. Occiput, Postparietal bone, presence in adult present (0); absent (1).
292. Occiput, Tabular bone, presence in adult present (0); absent (1).
293. Occiput, Suture separating basioccipital and exoccipital, presence in adult present (0); absent (1).
294. Occiput, Suture separating exoccipital and supraoccipital, presence in adult present (0); absent (1).
295. Occiput, Occipital bone, dorsal extent less or equal to one-half height of occiput(0); greater than one-half height of occiput (1).
296. Occiput slope occiput slopes posterodorsally, or vertically from occipital condyles (0); occiput slopes anterodorsally from occipital condyles (1).
297. Occipital, Hypoglossal foramen confluent with jugular foramen or sharing depression with jugular foramen (0); separate from jugular foramen (1).
298. Occipital, Hypoglossal foramina, number one (0); two (1).
299. Braincase, External bulging of braincase in parietal region absent (0); expanded, parietal part of cranial vault wider than frontal part, but expansion does not extend to lambdoidal region (1); greatly expanded, expansion of cranial vault extends to lambdoidal region (2).
300. Endocranium, Endocast flexure, measured as acute angle between two lines, one through olfactory bulb and circular fissure, and other through foramen magnum and pituitary gland greater 25 degree (0); equal or less than 25 degree (1).
301. Endocranium, Olfactory bulb casts, size in percent of total endocranial cast >12% (0); 6-12% (1); <6% (2).
302. Endocast, Circular fissure (separating olfactory bulbs from cerebral hemispheres) on endocasts, presence absent (0); present (1).
303. Endocranium, Cerebral hemisphere cast, lateral extent most lateral point of cerebral cast is medial to or even with the parafloccular cast (0); cerebral cast clearly extends laterally beyond parafloccular cast (1).
304. Endocranium, Ossified tentorium septum, presence present (0); absent (1).
305. Endocranium, Vermis, size small (0); enlarged (1).
306. Endocranium, Subarcuate fossa, presence present (0); absent (1).
307. Endocranium, Subarcuate fossa, depth deep (0); shallow (1).
308. Endocranium, Subarcuate fossa position relative to internal acoustic meatus posterodorsal (0); dorsal (1).
309. Endocranium, Cava epipterica, size in percent of total endocast >0.5% (0); 0.5% or less (1).
310. Endocranium, Pila antotica, presence present (0); absent (in adult) (1).
311. Endocranium, Pila antotica, extent of ossification present as complete bar (0); only ventromedial part ossified (1).
312. Endocranium, Sigmoid sinus sulcus, presence present (0); absent (1).
313. Endocranium, Sigmoid sinus sulcus, extent extends to jugular foramen (0); extends to foramen magnum (1).
314. Endocranium, Inferior petrosal sinus intrapetrosal (0); intramural (1); endocranial (2); no indication (3).
315. Mandible, Horizontal ramus of dentary, shape long and shallow (length/depth ratio greater than or equal 4) (0); intermediate (length/depth ratio > 2 and < 4) (1); short and deep (length/depth ratio lesser than or equal to 2) (2).

316. Mandible, Dentary symphysis fused (0); unfused (1).
317. Mandible, Dentary symphysis, orientation vertical (0); oblique or nearly horizontal (1).
318. Mandible, Mental foramina, morphology multiple small perforations (0); well-developed foramen or foramina (1).
319. Mandible, Mental foramina, number (only applicable in taxa with well-developed foramina one (0); two (1); three (2); four (3); five or more (4).
320. Mandible, Mental foramina, posterior-most mental foramen, vertical position ventral to midline of mandible (0); at approximate midline of mandible (1); dorsal to midline of mandible (2).
321. Mandible, fully developed postdentary trough (behind tooth row), presence present (0); absent (1).
322. Mandible, well-developed Meckelian groove in adults (extending anterior to mandibular foramen), presence present (0); absent (1).
323. Mandible, Meckelian groove in adult, curvature under tooth row parallel to ventral border of mandible (0); convergent to ventral border of mandible (1).
324. Mandible, Groove for replacement dental lamina, presence present (0); absent (1).
325. Mandible, Angular process, presence absent (0); present (1).
326. Mandible, Angular process, orientation posteroventrally directed (can be slightly inflected) (0); posteriorly directed, straight (1); transversely flaring (this is different from character state [3] in having a lateral expansion of the angle)(2); strongly medially inflected (3).
327. Mandible, Angular process, anteroposterior position relative to dentary condyle anterior position, angular process below main body of coronoid process (0); posterior position, angular process placed at same level as posterior border of coronoid process (1).
328. Mandible, Angular process, vertical position low, at or near level of ventral border of mandibular horizontal ramus (0); high, at or near level of molar alveolar line (1).
329. Mandible, Excavated fossa for reflected lamina of angular bone on dentary, presence present (0); absent (1).
330. Mandible, Coronoid bone, or its attachment scar in adults, presence present (0); absent (1).
331. Mandible, Medial pterygoid ridge or shelf along ventral border of body of mandible, presence absent (0); present (1).
332. Mandible, Medial pterygoid ridge or shelf, depth forming distinc ridge (0); medially extended forming deep pterygoid fossa (1).
333. Mandible, Medial pterygoid ridge or shelf, posterior direction confined ventrally (0); reaching dentary condyle (1).
334. Mandible, Mandibular foramen, position below or near base of anterior border of coronoid process (0); posterior to anterior border of coronoid process (1).
335. Mandible, Masseteric fossa, anteroventral margin crest, presence: absent (0); present (1).
336. Mandible, Masseteric fossa, anteroventral margin crest, development low crest (0); well-defined crest (1).
337. Mandible, Masseteric fossa, anterodorsal margin crest, presence: absent or weakly developed (0); present as distinct anterior border (1).
338. Mandible, Masseteric fossa, anterior extension onto body of mandible (below tooth row), presence absent (0); present, extending anteriorly to below ultimate postcanine (1); present, extending anteriorly beyond ultimate postcanine (2).
339. Mandible, Masseteric foramen (=labial mandibular foramen), presence: absent (0); present (1).
340. Mandible, Coronoid process, tilting (measured as angle between anterior border of coronoid process and horizontal alveolar line of all molars) coronoid process strongly reclined, forming obtuse angle greater than 145" (0); coronoid process less

reclined, 135-145" (1); coronoid process less than vertical, 110-135" (2); coronoid process near vertical, 95-110" (3).

341. Mandible, Coronoid process, alignment of ultimate molar to anterior margin of coronoid process ultimate molar medial to coronoid process (observation of ultimate molar completely obstructed by coronoid process in lateral view) (0); ultimate molar in alignment with, or mesial to, anterior margin of coronoid process (some portion or all of ultimate molar visible anterior to coronoid process in lateral view) (1).
342. Mandible, mylohyoid process at level of anterior border of coronoid process, presence absent (0); present (1).
343. Mandible, Dentary peduncle (narrowing between dentary condyle and rest of dentary), presence present (0); absent (1).
344. Mandible, Dentary condyle, direction posteriorly directed, forms angle of equal or smaller than 35" (0); vertically directed, forms angle of >35" (1).
345. Mandible, Dentary articulation, condyle shape dorsoventrally compressed (0); bulbous, equally as mediolaterally wide as long in dorsal view (1); mediolaterally narrow, markedly longer than mediolaterally wide in dorsal view forming vertically deep arc(2); transversely broad, markedly mediolaterally wider than long in dorsal view (3).
346. Mandible, Dentary condyle, position relative to vertical level of postcanine alveoli below or about same level as postcanine alveoli (0); above level of postcanine alveoli (1).
347. Mandible, Craniomandibular joint, participation of surangular-articular-prearticular in articulation with cranium, presence present, participating in craniomandibular joint (0); absent, excluded from craniomandibular joint (1).
348. Tooth implantation subthecodont (0); thecodont (1).
349. Postcanines, replacement of at least some distal teeth, presence present (0); absent (1).
350. Postcanines, distal migration (loss of mesial and addition of distal postcanines), presence present (0); absent (1).
351. Diastema between I2 and I3 (applicable only if I3 present), presence absent (0); present (1).
352. Diastema distal to ultimate upper incisor, presence absent (0); present (1).
353. Diastema distal to upper canine (only applicable if canine present), presence: absent (0); present (1).
354. Diastema separating any upper premolars (distance equal or larger than half of the P1), presence absent (0); present (1).
355. Diastema distal to ultimate lower incisor, presence absent (0); present (1).
356. Diastema distal to lower canine (only applicable if canine present), presence absent (0); present (1).
357. Lower incisors, number in each quadrant five (0); four (1); three (2); two (3); one (4); none (5).
358. Upper incisors, number in each quadrant five (0); four (1); three (2); two (3); one (4); none (5).
359. Upper incisor, I1 gliriform, presence absent (0); present (1).
360. Upper incisor, I2 gliriform, presence absent (0); present (1).
361. Upper incisor or incisors, position all implanted at or near margin of palate (0); distal incisors implanted medial to margin of palate (1).
362. Upper incisor, I1, enamel covering of crown covers whole crown (0); restricted to buccal surface (1).
363. Upper incisor, I2, enamel covering of crown covers whole crown (0); restricted to buccal surface (1).
364. Upper incisor, I2, cusp, number multiple cusps (0); single cusp (1).
365. Upper incisor, I3, cusp, number multiple cusps (0); single cusp (1).

366. Lower incisors, staggered (only applicable to taxa with more than one incisor) absent (0); present (1).
367. Lower incisor, i1, gliriform, presence absent (0); present (1).
368. Lower incisor, i1, enamel covering of crown covers whole crown (0); restricted to buccal surface (1); lacks enamel (2).
369. Lower incisor, i1, position of distal end of root relative to postcanines mesial to level of premolars (0); opposite level of premolars (1); opposite level of molars (2).
370. Upper canine, presence present (0); absent (1).
371. Upper canine, size larger than neighboring teeth (0); similar to or smaller than neighboring teeth (1).
372. Upper canine, roots, number single (0); double (1).
373. Upper canine, cusp, number single (peg-like) (0); two or more cusps (1).
374. Lower canine, presence present (0); absent (1).
375. Lower canine, size larger than neighboring teeth (0); similar to or smaller than neighboring teeth (1).
376. Lower canine, roots, number single (0); double (1).
377. Lower postcanine teeth, number in each quadrant ten or more (0); nine (1); eight (2); seven (3); six (4); five (5); four (6); three or fewer (7).
378. Upper postcanine teeth, number in each quadrant more than eight (0); eight (1); seven (2); six (3); five (4); four (5); three or fewer (6).
379. Lower postcanine tooth row, orientation in dorsal view parallel or nearly parallel to major axis of dentary (0); markedly oblique to major axis of dentary (1).
380. Lower premolars, number in each quadrant five or more (0); four (1); three (2); two (3); one (4); none (5).
381. Upper premolars, number in each quadrant five or more (0); four (1); three (2); two (3); one (4); none (5).
382. Upper ultimate premolar (P5) protocone (not applicable to taxa with multi-rowed cheek teeth) absent (0); present (1).
383. Upper ultimate premolar (P5), metacone (not applicable to taxa with multi-rowed cheek teeth absent (0); present (1).
384. Upper ultimate premolar (P5)/first upper molar (M1) length ratio >1.5 (0); 1.5-0.8 (1); <0.8 (2).
385. Upper ultimate premolar (P5)/first upper molar (M1) width ratio >0.9 (0); 0.9-0.6 (1); 0.59-0.45 (2); <0.45 (3).
386. Upper ultimate premolar (P5), roots three or more (0); two (1); single (2).
387. Upper ultimate premolar (P5), number of tooth rows one (0); two or more (1).
388. Upper ultimate premolar (P5), basined (with main cusps located peripherally surrounding a shallow and broad central basin) (only applicable to taxa with multi-rowed cheek teeth) absent (0); present (1).
389. Upper ultimate premolar (P5), mesiobuccal bulge, presence (only applicable to taxa with multi-rowed cheek teeth) absent (0); present (1).
390. Upper ultimate premolar (P5), cusps, total number (only applicable to taxa with multi-rowed postcanines) 3 or fewer (0); 4-6 (1); 7 or more (2).
391. Upper ultimate premolar (P5), enamel ridges or crenulations, presence absent (0); present (1).
392. Lower penultimate premolar (p4) size small and subequal to other premolars (0); substantially larger than more mesial premolars but substantially smaller than ultimate lower premolar (p5 or dp5) (1); largest (longer and/or taller) tooth in premolar series (2).
393. Lower penultimate premolar (p4), paraconid (cusp b), presence absent or indistinct (0); present, distinct or well developed as important cusp (1).
394. Lower penultimate premolar (p4), roots, number three or more (0); two (1); one (2).
395. Lower penultimate premolar (p4), buccal basal cuspules (applicable to bladed lower premolars), presence present (0); absent (1).

396. Lower premolars, contact of penultimate (p4) and ultimate (p5) non-overlapping (0); overlapping or staged, p5 overhanging p4 (1).
397. Lower ultimate premolar (p5), size small, with area less than that of m1 (0); large, with area greater than, or subequal to, that of m1 (1); hypertrophied, with area 1.5 times greater than that of m1 (2).
398. Lower ultimate premolar (p5), cusp alignment principal cusps mesiodistally aligned (0); principal cusps arranged in triangle (1); principal cusps arranged in quadrangle (2).
399. Lower ultimate premolar (p5), outline buccolingually compressed, crown outline longer than wide (0); buccolingually broad, crown outline subequal or wider than long (1); buccolingually very compressed, blade-like (2).
400. Lower ultimate premolar (p5), symmetry of protoconid (cusp a) asymmetrical, mesial edge of cusp a more convex in outline than distal edge (0); symmetrical, mesial and distal cutting edges equal or subequal in length (1).
401. Lower ultimate premolar (p5), paraconid (cusp b) absent or indistinct (0); present as distinct or well-developed cusp (1).
402. Lower ultimate premolar (p5), protoconid (cusp a), relative height to metaconid (cusp c); measured as height ratio of a and c from bottom of valley between two adjacent cusps metaconid absent or very small (0); metaconid distinctive but <30% of protoconid (1); metaconid and protoconid equal or subequal in height (40-100% of protoconid) (2).
403. Lower ultimate premolar (p5), hypoconid (cingulid cusp d) absent (0); present (1).
404. Lower ultimate premolar (p5), distal cingulid, presence absent (0); present (1).
405. Lower ultimate premolar (p5), buccal cingulid, presence absent or vestigial (0); present (1).
406. Lower ultimate premolar (p5), buccal cingulid, extent extends one-half length of crown or less (0); extends more than one-half length of crown (1).
407. Lower ultimate premolar (p5), buccal cingulid cuspules, presence absent (0); present (1).
408. Lower ultimate premolar (p5), lingual cingulid, presence absent or vestigial (0); present (1).
409. Lower ultimate premolar (p5), roots, number three or more (0); two (1); single (2).
410. Lower ultimate premolar (p5), organization of multiple cusps and rows (only applicable to taxa with multi-rowed postcanines): two rows (0); one row (1).
411. Lower ultimate premolar (p5), serration count, only applicable to blade-like premolar: 7 or less (0); 8-10 (1); 11 or more (2).
412. Lower ultimate premolar (p5), mesiobuccal exodaenodont lobe, presence (only applicable to taxa with multi-rowed postcanines) absent (0); present (1).
413. Lower molars, number in each quadrant six or more (0); five (1); four (2); three (3); two or fewer (4).
414. Upper molars, number in each quadrant six or more (0); five (1); four (2); three (3); two or fewer (4).
415. Molars (or distal postcanines), transverse widening of upper relative to lower upper teeth not wider (0); upper teeth wider, up to 1/3 wider than lowers (1); upper teeth much wider than the lowers (> 1/3) (2).
416. Molars (or distal postcanines), hypsodonty, presence absent (0); present (1).
417. Molars (or distal postcanines), islets/infundibula, presence absent (0); present (1).
418. Molars (or distal postcanines), infundibula, penetrate deeply into crowns, presence absent (0); present (1).
419. Molars (or distal postcanines), synclines/furrows, presence on at least one side of crown, presence absent (0); present (1).
420. Molars (or distal postcanines), furrows, extend to base of crown and onto root, presence absent (0); present (1).
421. Molars (or distal postcanines), transverse lophs across the interior of crown (not along mesial or distal end), presence absent (0); present (1).

422. Molars (or distal postcanines), transverse lophs/lobes across the interior of crown (not along mesial or distal end), number one (0); two or more (1).
423. Molars (or distal postcanines), enamel distribution present on all sides of tooth crown (0); absent on at least one side of tooth crown on at least one distal postcanine (1).
424. Molars (or distal postcanines), multiple rows of cusps, presence absent (0); present (1).
425. m1-2 (or distal postcanines), interlocking, presence absent (0); present (1).
426. m1-2 (or distal postcanines) interlocking, type distal-most part of preceding molar fits in between mesial cingulid cuspules e and f of succeeding molar (0); distal-most part of preceding molar fits between mesial cingulid cuspule e and paraconid (cusp b) of succeeding molars (1); distal-most part of preceding molar fits into embayment or vertical groove of mesial aspect of paraconid (cusp b) of succeeding molars (2); mesial-most part of succeeding lower molars overlapping distal-most part of preceding lower molars (3).
427. m1-2, paraconid (cusp b), presence (not applicable to taxa with multi-row cheek teeth) present (0); absent (1).
428. m1-2, paraconid (cusp b), mesiolingual surface, shape rounded (0); forms keel (1).
429. m1-2, paraconid (cusp b), proximity to metaconid (cusp c) bases widely separated (0); bases approaching each other becoming confluent (1); single cusp (amphyconid) (2).
430. m2 (if possible), paraconid (cusp b), relative height to metaconid (cusp c) c taller than b (0); b and c subequal in height (1); b taller than c (2).
431. m1-2, paracristid, orientation relative to longitudinal axis of molars longitudinal orientation (0); oblique (1); nearly transverse (2).
432. m1-2, protoconid (cusp a), buccal curvature at base level relative to curvature of paraconid (cusp b) and metaconid (cusp c) cusps have same degree of labial bulging (0); protoconid far more bulging than paraconid and metaconid (1).
433. m1-2, metacristid (protocristid) crest between protoconid (cusp a) and metaconid (cusp c), orientation of relative to long axis of lower molars parallel to lower jaw axis (0); oblique (1); transverse (2).
434. m1-2, distal metacristid, presence present (0); absent (1).
435. m1, main cusps of trigonid, alignment (not applicable to taxa with multi-row cheek teeth) single longitudinal row (0); acute angle (1); obtuse angle (2).
436. m1-2, primary functional cusps, labiolingual compression (at level of cusp base but above cingulid) absent (0); present (1).
437. m1-2, hypoconid (cusp d = hypoconid in Luo, Chen et al., 2007; Zheng et al., 2013:char. 86; Zhou et al., 2013:char. 97; = hypoconulid in Rougier et al., 2011), development small cusp at cingulid level (0); well-developed as important cusp of talonid (1).
438. m1-2, cristid oblique, presence absent (0); present (1).
439. m1-2, cristid obliqua, orientation to metaconid (distal aspect of metaconid)(0); to metaconid-protoconid notch (center of to slightly lingual to notch) (1); to protoconid (pointed mesially between metaconid-protoconid notch and protoconid) (2).
440. m1-2, hypoconulid, presence absent (0); present (1).
441. m1-2, hypoconulid, orientation cusp tip erect or procumbent (0); cusp tip recumbent (reclined posteriorly) (1).
442. m1-2, hypoconulid, mediolateral position on talonid (character only applicable to taxa with multicuspidate talonid developed) at median position (0); at more lingual position (1).
443. m1-2, prehypoconulid, crest connecting metaconid with hypoconulid along lingual edge of tooth, presence absent (0); present (1).
444. m1-2, entoconid, presenceabsent (0); present (1).

445. m1-2, entoconid, height compared to other cusps of talonid lower than hypoconulid (or even vestigial) (0); subequal in height to hypoconulid, or taller (1).
446. m1-2, paraconid, metaconid, and entoconid, alignment (applicable only to taxa with triangulation of trigonid cusps and entoconid present on talonid) cusps not aligned (0); cusps aligned (1).
447. m1-2, entocristid on talonid heel, presence (can be scored without entoconid being present) talonid lacks medial and longitudinal crest (0); pre-entoconid cristid of talonid in alignment with metaconid or with
postmetacristid if latter present
pre-entoconid cristid of talonid in alignment with metaconid or with
postmetacristid if latter present (1); pre-entocristid crest offset from metaconid, lingual to base of metaconid (2).
448. m1-2, talonid basin, presence absent (0); present (1).
449. m1-2, talonid/trigonid width ratio narrow (talonid <40% of trigonid) (0); wide (talonid is 40-70% of the trigonid) (1); very wide (talonid width >70% of trigonid width) (2).
450. m1-2, talonid basin, aspect ratio in occlusal view (length versus width) at cingulid level longer than wide (0); length equals width (1); wider than long (2).
451. m1-2, talonid, elevation hypoconid/protoconid height ratio20% or less (0); hypoconid/protoconid height ratio 20-35% (1); hypoconid/protoconid height ratio 35-50% (2); hypoconid/protoconid height ratio 50% or more (3).
452. m1-2 (or distal postcanines), cingular cuspule e, presence present (0); absent (1).
453. m1-2 (or distal postcanines), cingular cuspule f, presence absent (0); present (1).
454. m1-2 (or distal postcanines), mesial cingulid, presence absent (0); present (1).
455. m1-2 (or distal postcanines), mesial cingulid, development restricted to mesial aspect of paraconid base (0); extending along most of lingual base of paraconid (1).
456. m1-2 (or distal postcanines), buccal postcingulid, presence absent (0); present (1).
457. m1-2 (or distal postcanines), postcingulid orientation oblique, connected to hypoconid (0); horizontal above gum level (1).
458. m1-2 (or distal postcanines), distal lingual cingulid, presence absent or weak (0); distinctive (1); strongly developed, crenulated with distinctive cuspules (2).
459. m1-2 (or distal postcanines), roots, number on each one (0); two (1); three or more (2).
460. m1-2 (or distal postcanines), roots, length > three times crown height (0); less than or equal to three times crown height (1).
461. m1-2 (or distal postcanines), roots, orientation straight (0); apical ends inclined or bent posteriorly (1).
462. m1 (or one of the distal lower postcanines), cusps, total number (only applicable to taxa with multi-rowed postcanines) 4 or fewer (0); 5-8 (1); 9-12 (2); 13 or more (3).
463. m1-2 (or distal postcanines), distal aspect, morphology (only applicable to taxa with multi-rowed postcanines) open (0); closed (by cuspules or ridge) (1).
464. m1-2 (or distal postcanines), cuspules or ridges of central basin, presence (only applicable to taxa with multi-rowed postcanines) absent (0); present (1).
465. m1-2 (or distal postcanines), U-shaped transverse ridge, presence (only applicable to taxa with multi-rowed postcanines):absent (0); present (1).
466. m1-2 (or distal postcanines), U-shaped transverse ridge, position (only applicable to taxa with multi-rowed postcanines):at second anterior cusp (0); at anterior rim (1).
467. m1-2 (or distal postcanines), cusps, relative height (only applicable to taxa with multi-rowed postcanines) at essentially same level (0); at distinctly different levels (1).
468. m2 (or ultimate postcanine), middle valley between lingual and buccal cusp row, presence (only applicable to taxa with multi-rowed cheek teeth) absent (0); present (1).

469. m2 (or ultimate postcanine), buccal row, length (only applicable to taxa with multi-rowed cheek teeth) subequal to lingual row (0); shorter than lingual row anteriorly (by at least half cusp length) (1); longer than lingual row posteriorly (by at least half cusp length) (2).
470. M1-2 (or distal postcanines), tongue-in-groove interlock, presence absent (0); present (1).
471. M1-2, functional protocone (that grinds against basin on lowers), presence absent (0); present (1).
472. M1-2, protocone, transverse width narrow (distance from protocone apex to paracone apex less or equal to 0.60 of total tooth width) (0); strongly transverse (distance from protocone apex >0.60 of total tooth width) (1).
473. M1-2, lingual region, mesiodistal development (in taxa with M1-2 protocones) narrow (anteroposterior distance medial to the paracone and metacone less than 0.30 of total tooth length) (0); moderate development (distance between position of conules equals 0.31-0.50 of total tooth length) (1); long (distance between conules greater than 0.51 of total tooth length) (2).
474. M1-2, preprotocrista, length terminates midway between protocone and paracone apices (0); terminates mesial to paracone apex (1); terminates buccal to paracone apex (2).
475. M1-2, postprotocrista, development (applicable only to molars with reversed triangulation of molar cusps) postprotocrista is short and does not extend labially beyond metacone (0); postprotocrista is long and extends labially beyond metacone (1).
476. M1-2, paraconules, presence absent or very small (0); present (1).
477. M1-2, metaconules, presence absent or very small (0); present (1).
478. M1-2, paracone (cusp A), orientation erect (0); recumbent (1); procumbent (2).
479. M1-2, preparacrista, (crest emerging from mesial face of paracone or cusp A), presence absent (0); present (1).
480. M1-2, preparacrista, orientation preparacrista oriented mesially, <30" (0); preparacrista oriented mesiobuccally, 30-59" (1); preparacrista oriented buccally, >60" (2).
481. M1-2, metacone (cusp C), presence present (0); absent (1).
482. M1-2, metacone (cusp C), position distal to paracone (0); distobuccal to paracone (1); distolingual to paracone (2).
483. M1-2, paracone (cusp A), base merged with base of metacone (cusp C) merged (0); separated (1).
484. M1-2, paracone (cusp A), relative height and size to metacone (cusp C) paracone higher and larger than metacone (0); metacone higher and larger than paracone (1); subequal (2).
485. M1-2, postparacrista and premetacrista (continuous crest between paracone and metacone), presence absent (0); present (1).
486. M1-2, postparacrista and premetacrista, orientation straight (0); V-shaped, with labially directed postparacrista and premetacrista (1).
487. M1-2, stylar shelf, presence absent (0); present (1).
488. M1-2, stylar shelf, width broad (0); narrow (1).
489. M1-2, stylocone (stylar cusp B), presence absent (0); present (1).
490. M1-2, stylocone (stylar cusp B), size small stylar cusp (0); prominent cusp subequal or larger than paracone (1).
491. M1-2, stylocone (stylar cusp B), position along buccal edge (0); separated (1).
492. M1-2, stylocone (stylar cusp B), relationship stylocone connected to paracrista or mesial to its end (0); stylocone distal to labial end of paracrista (1); stylocone detached from preparacrista occupying central position on crown (2).
493. M1-2, mesostyle (stylar cusp C, near the ectoflexus), presence absent (0); present (1).

494. M1-2, stylar cusp D (opposite metacone following Reig et al., 1987), presence absent (0); present (1).
495. M1-2, metastyle (stylar cusp E following Reig et al., 1987), presence absent (0); present (1).
496. M1-2, metastyle (stylar cusp E following Reig et al., 1987), "twinning" with accessory cusp, presence absent (0); present (1).
497. M1-2, parastylar hook, presence absent or poorly developed (0); present (1).
498. M1, ectoflexus, presence strongly reduced or absent (0); present (1).
499. M2, ectoflexus, presence strongly reduced or absent (0); present (1).
500. M1-2 (or distal postcanines), buccal cingulum, presence absent or weak (0); present (1).
501. M1-2, (or distal postcanines) lingual cingulum, presence present (0); vestigial or absent (1).
502. M1-2, (or distal postcanines) lingual cingulum, development continuous cingulum (0); discontinuous cingulum (1).
503. M1-2, precingulum, size narrow and closely appressed to crown or absent (0); distinct, broad (1).
504. M1-2, postcingulum, size narrow and closely appressed to crown or absent (0); distinct, broad (1).
505. M1 and M2 (or distal postcanines), roots, number on each one (0); two (1); three (2); more than three (3).
506. M1-2, lingual root, presence absent (0); present (1).
507. M1-2, lingual root, position under paracone (0); under protocone or trigon (1).
508. M1 (or one of the distal upper postcanines), cusps, total number8 or fewer (0); 9-16 (1); 17 or more (2).
509. M1 (or one of the distal upper postcanines), rows of cusps, number (only applicable to taxa with multi-rowed postcanines) two (0); three (includes distolingual wing) (1).
510. M1 (or one of the distal upper postcanines), third lingual row (distolingual wing), length compared to total length of tooth (only applicable to taxa with three rows present) >0.9 (equal in length) (0); 0.5-0.9 (greater than half length) (1); <0.5 (less than half length) (2).
511. M1-2 (or distal postcanines), mesial U-ridge, presence (only applicable to taxa with multi-rowed cheek teeth) absent (0); present, closed by ridge (1); present, closed by cuspules (2).
512. M1-2 (or distal postcanines), cuspules and/or transverse fluting of the central basin, presence (only applicable to taxa with multi-rowed cheek teeth) absent (0); present (1).
513. M1 (or penultimate postcanine), cusps, relative height (only applicable to taxa with multi-rowed postcanines) at essentially same level (0); at distinctly different levels (1).
514. M2 (or distal postcanines), alignment with M1 (only applicable to taxa with multi-rowed cheek teeth) aligned, M2 not lingually offset to M1 (0); not aligned, M2 lingually offset to M1 (1).
515. Enamel, microstructure, type synapsid columnar enamel (includes "transitional enamel" of Mao et al. [2017]) (0); prismatic enamel (includes both "plesiomorphic prismatic enamel" of Wood and Rougier [2005] and "true prismatic enamel" of Mao et al. [2017]) (1).
516. Enamel, prism sheath, shape (only applicable to taxa with enamel prisms) open, arc-shaped, does not fully enclose prism head ("key hole pattern" of Koenigswald and Sander, 1997) (0); closed, circular, fully encloses prism head (1).
517. Enamel, interprismatic matrix radial enamel, interprismatic matrix anastomoses around prisms and does not form interrow sheets (0); modified radial enamel, distinct interrow sheets of interprismatic matrix (1).

518. Enamel, prism, size (only applicable to taxa with enamel prisms) small (normal): average diameter <7.0 um (0); large (gigantoprismatic): average diameter 0.7 um or greater (1).
519. Discrete wear facets formed between occluding cusps of opposing upper and lower postcanine teeth, presence absent (0); present (1).
520. Relationships between cusps of opposing upper and lower molar teeth protoconid (cusp a) occludes in groove between paracone (cusp A) and stylocone (cusp B) (0); protoconid (cusp a) occludes in front of stylocone (cusp B) and into embrasure between opposing and preceding upper teeth (1); part of talonid occludes with lingual face (or any part) of upper molar (2); lower multicuspate rows alternately occlude between upper multicuspate rows (3); columnar tooth without cusps and with beveled wear across entire crown contact surface (4); primary cusp of lower tooth contacts distolingual ridge on upper tooth, then slides through mesiolingual notch of succeeding tooth and into its basin (5).
521. Direction of dentary movement during power stroke of chewing cycle essentially dorsal movement (orthal) (0); essentially horizontal movement with pronounced anteromedial translation (1); essentially horizontal movement with anterior translation (proal) (2); dorsoposterior (3); essentially horizontal movement with posterior translation (palinal) (4); essentially horizontal movement with posterolateral translation (5).
522. Molar, Prevallum/postvallid shearing, presence absent (0); present (1).
523. Lower molars, wear facets 1 and 2, presence absent (0); present (1).
524. Molars, wear facets 3 and 4, presence absent (0); facet 3 present (1); facets 3 and 4 present (2).
525. Molars, wear facet 4, orientation facet oriented obliquely with respect to long axis of tooth (0); facet oriented transversely with respect to long axis of tooth (1).
526. Molars, wear facets within talonid basin, presence absent (0); present (1).
527. Upper molar, worn occlusal surface with wide concave area labially and narrow flat area lingually (only applicable to taxa with flat occlusioal surface) absent (0); present (1).
528. Lower molars, presence of multiple ridges within talonid basin smooth surface on the talonid (or on cusp d) (0); multiple ridge within talonid basin (1).
529. M1-2 (or distal postcanines), fusiform ("spindle-shaped") shearing valley, presence (only applicable to taxa with multi-rowed postcanines) absent (0); present (1).
530. m1-2 (or distal postcanines), fusiform ("-'spindle-shaped"--) shearing valley, presence (only applicable to taxa with multi-rowed postcanines) absent (0); present (1).

Character list of the Martinelli et al.[47] data matrix. The characters scored in

***Patagomaia* are indicate in yellow**.

1 - Symphysis Height: 0. Robust, verticalized; 1. Slender, oblique, or approaching horizontal.
2 - Position of Posteriormost Mental Foramen: 0. Below the canine and anterior premolariform region; 1. Below the penultimate premolar; 2. Below the ultimate premolar; 3. Between the ultimate premolar and the first molar.
3 - Postdentary Trough. Behind tooth row: 0. Present; 1. Absent.
4 - Mandibular Alveolar Border: 0. Subequal in height to the lingual edge; 1. Labial border much lower than lingual.
5 - Degree of Development of Meckelian Groove in Adults: 0. Well developed; 1. Weakly developed; 2. Vestigial or absent.

6 - Curvature of Meckelian Groove in Adults. Under the tooth row: 0. Parallel to the ventral border of mandible; 1. Convengent to the ventral border of mandible.
7 - Groove for Replacement Dental Lamina: 0. Present; 1. Absent.
8 - Angular Process Presence: 0. Absent; 1. Present.
9 - Angular Process Direction: 0. Small knobby process, not projected; 1. Straight process, posteriorly directed; 2. Transversely flaring; 3. Inflected; 4. Downturned.
10 - Antero-Posterior Position of Angular Process Relative to Dentary Condyle: 0. Anterior position, the angular process is below the main body of the coronoid process; 1. Posterior position, the angular process is placed at the level of the posterior end of the coronoid process.
11 - Vertical Position of Angular Process: 0. Low, at or near the level of the ventral border of the mandibular horizontal ramus; 1. High, at or near the level of the molar alveolar line.
12 - Coronoid, or Its Attachment Scar, in Adults: 0. Present; 1. Absent.
13 - Medial Fossa on Dentary Angular Process: 0. Present; 1. Absent.
14 - Pterygoid Fossa: 0. Absent; 1. Present.
15 - Medial Pterygoid Ridge or Shelf Direction: 0. Directed to angular process; 1. Reaching the dentary condyle via a low crest.
16 - Pterygoid Shelf: 0. Absent; 1. Present.
17 - Ventral Border of Masseteric Fossa: 0. Absent; 1. Present as a low and broad crest; 2. Present as a well-defined and thin crest.
18 - Position of Mandibular Foramen: 0. Below or near to the base of the anterior border of the coronoid process; 1. Posterior to the anterior edge of the coronoid process.
19 - Masseteric Foramen: 0. Absent; 1. Present.
20 - Crest of Masseteric Fossa Along Anterior Border of Coronoid Process: 0. Absent or weakly developed; 1. Present as a distinct anterior border.
21 - Mylohyoid Process at Level of Anterior Border of Coronoid Process: 0. Absent; 1. Present.
22 - Orientation of Dentary Peduncle and Condyle: 0. Dentary peduncle is posteriorly directed, forms an angle of 40° or less to the alveolar margin; 1. Vertically directed dentary peduncle, above 40°; 2. Dentary condyle is continuous with the semicircular posterior margin of the dentary.
23 - Lower Mandibular Margin/Condylar Peduncle: 0. Not continuous, interrupted by an angular process or a sharp angle; 1. Continuous as a single line in lateral view.
24 - Shape and Relative Size of Dentary Articulation: 0. Small and dorsoventrally compressed; 1. Condyle is massive and bulbous, transversely broad in its dorsal aspect; 2. Condyle mediolaterally narrow and vertically deep, forming a broad arc in lateral outline, either ovoid or triangular in posterior view.
25 - Ventral Border of Dentary Peduncle: 0. Posteriorly tapering without a condyle; 1. Columnar or ridge-like; 2. Ventrally flaring; 3. Robust and short.
26 - Position of Dentary Condyle Relative to Vertical Level of Postcanine Alveoli: 0. Below or about the same level as the postcanine alveoli; 1. Above the level of the postcanine alveoli.
27 - Tilting of Coronoid Process of Dentary. Measured as the angle between the imaginary line of the anterior border of the coronoid process and the horizontal alveolar line of all molars: 0. Coronoid process is strongly reclined forming an obtuse angle; 1. Coronoid process less reclined, 135–145°; 2. Coronoid process less than vertical, 115–125°; 3. Coronoid process is near vertical and the angle is small, 95–105°.
28 - Retromolar Space. At least half the length of the last molar: 0. Absent; 1. Present.
29 - Alignment of Ultimate Molar to Anterior Margin of Dentary Coronoid Process: 0. Ultimate functional molar is medial to the coronoid process; 1. Ultimate functional molar is in alignment with the anterior margin of coronoid process.
30 - Shape of Ventral Edge of Jaw in Area of Angular Process: 0. Straight; 1. Convex; 2. Concave.

31 - Incisor Replacement: 0. Alternating and multiple replacement; 1. Diphyodont replacement or none.
32 - Number of Lower Incisors: 0. Three or more; 1. Two or fewer.
33 - Maxillary Incisor: 0. Present; 1. Absent.
34 - Staggered Incisor: 0. Absent; 1. Present.
35 - Canine Replacement: 0. Multiple replacements; 1. Diphyodont.
36 - Canines: 0. Present and large; 1. Present and small; 2. Absent.
37 - Upper Canine Height: 0. Long, at least twice the height of tallest postcanine; 1. Short, less than twice the height of the tallest postcanine.
38 - Long Upper Canine Height: 0. Relatively short, less than three times the height of the tallest postcanine crown; 1. Tall, more than three times the height of tallest postcanine crown.
39 - Replacement of Premolariforms: 0. Multiple replacement; 1. One replacement or none.
40 - Total Number of Premolars. Lower premolar preferred; uppers used if no lowers are available: 0. Two or fewer; 1. Three premolars; 2. Four premolars; 3. Five or more.
41 - Diastema Separating P1 From P2. Distance equal or larger than half of the P1: 0. Absent; 1. Present.
42 - Penultimate Lower Premolar Size: 0. Small and subequal to other premolars; 1. Larger than any other premolar, longer and/or taller; 2. Hypertrophied, dominant tooth in the series.
43 - Penultimate Lower Premolar—Paraconid (Cusp b): 0. Much smaller than metaconid (cusp c) of the same tooth, or absent; 1. Well developed as an important cusp of trigonid.
44 - Last Lower Premolar—Symmetry of Main Cusp a (Protoconid): 0. Asymmetrical, anterior edge of cusp a is more convex in outline than the posterior edge; 1. Symmetrical, anterior and posterior cutting edges are equal or subequal in length.
45 - Last Lower Premolar—Anterior Cusp b (Paraconid): 0. Present, at least subequal to cusp c, or posterior cingular cusp of the same tooth; 1. Small, much smaller than cusp c or posterior cingular cusp of the same tooth, or vestigial to absent.
46 - Last Lower Premolar—Arrangement of Principal Cusp a, Cusp b (if Present), and Cusp c: 0. Aligned straight or at a slight angle; 1. Distinctive triangulation.
47 - Last Lower Premolar—Distinct Distal Cingulid Cusp d: 0. Absent; 1. Posterior cingular cusp present; 2. Present as part of a continuous distal cingulid.
48 - Last Lower Premolar Outline: 0. Laterally compressed, crown outline longer than wide; 1. Transversely wide, crown outline subequal or wider than long.
49 - Last Lower Premolar Size: 0. Small and subequal to other premolars; 1. Large tooth, taller than or subequal to first molar; 2. Hypertrophied, much larger than any molar.
50 - Labial Cingulid of Last Lower Premolar: 0. Absent or vestigial; 1. Present along more than half of the crown length.
51 - Lower Premolars Lingual Cingulid: 0. Absent or vestigial; 1. Present.
52 - Relative Height of Primary Cusp a to c of Last Lower Premolar. Measured as the height ratio of a and c from the bottom of the valley between the two adjacent cusps: 0. Posterior cusp c is absent or very small; 1. Posterior cusp c is distinctive but less than 30% of primary cusp a; 2. Posterior cusp c and primary cusp a are equal or subequal in height (c is 40–100% of a).
53 - Last Upper Premolar—Functional Protocone: 0. Absent; 1. Present.
54 - Last Upper Premolar—Parastylar Hook: 0. Present; 1. Absent or very small.
55 - Number of Molars or Molariform Postcanines. Lowers preferred, uppers used if no lowers are known: 0. Two molars; 1. Three molars; 2. Four to five molars; 3. Six or more.

56 - Alignment of Main Cusps of Posterior Lower Molars. m3 or more posterior teeth considered if present: 0. Simple longitudinal row; 1. Obtuse angle (>95); 2. Acute angle (<90).
57 - Alignment of Main Cusps of Anterior Lower Molar (m1): 0. Single longitudinal row; 1. Acute angle; 2. Obtuse angle.
58 - Development of Postprotocrista on Upper Molars for Double Rank Postvallum Shear. For molars with reversed triangulation of molarcusps: 0. Postprotocrista is short and does not extend labially beyond metacone; 1. Postprotocrista is long and extends labially beyond metacone.
59 - Precise Opposition of Upper and Lower Molars. Either one-to-one, or occluding at the opposing embrasure or talonid: 0. Absence of precise opposition of upper and lower molars; 1. Present (either one-to-one or occluding at the opposing embrasure or talonid).
60 - Relationships Between Cusps of Opposing Upper and Lower Molars: 0. Absent; 1. Present, lower primary cusp a occludes in the groove between upper cusp a and b; 2. Present, lower main cusp a occludes in front of upper cusp b and into the embrasure between the opposing and preceding upper teeth; 3. Present, part of the talonid occluding with the lingual face (or any part) of the upper molar; 4. Lower multicuspate rows alternately occlude between the upper multicuspate rows.
61 - Relative Height of Primary Cusp a (Protoconid) to Cusp c (Metaconid) of Anterior Lower Molars. Measured as the height ratio of a and c from the bottom of the valley between the two adjacent cusp, on m1: 0. Posterior cusp c is less than 40% of the primary cusp a (protoconid); 1. Posterior cusp c is more than 40% of cusp a.
62 - Relative Size/Height of Cusp b (Paraconid) to Cusp c (Metaconid). Based on m2 when possible: 0. c taller than b; 1. b and c subequal in height; 2. b taller than c.
63 - Relative Elevation of Bases of Paraconid (Cusp b) and Metaconid (Cusp c): 0. Almost or at the same level; 1. Base of the paraconid higher than base of the metaconid; 2. Base of metaconid higher than base of the paraconid.
64 - Cristid Obliqua: 0. Absent; 1. Present.
65 - Cristid Obliqua Orientation: 0. Oriented to or lingual to the metaconid-protoconid notch; 1. Hypertrophied and directed to posterior part of the metaconid; 2. Short and pointed anteriorly between the metaconid-protoconid notch and the protoconid.
66 - Lower Molar—Pre-Entocristid on Talonid Heel: 0. Talonid lacks medial and longitudinal crest; 1. Pre-entoconid cristid of talonid in alignment with the metaconid or with the postmetacristid if the latter is present; 2. Pre-entocristid crest is offset from the metaconid and it is lingual to the base of the metaconid.
67 - Labial Curvature of Primary Cusp a of Lower Molars (at Base Level) Relative to Curvature of Cusps b and c: 0. Cusps a, b, and c have the same degree of bulging; 1. Cusp a is far more bulging than cusps b and c.
68 - Labial Curvature of Main Cusps a, b, and c at Level of Cusp Valley of Penultimate and Ultimate Upper Molars: 0. Cusp a, b, and c have about the same degree of curvature; 1. Cusp a is slightly concave (or far less convex than either cusp b or c).
69 - Labiolingual Compression of Primary Functional Cusps of Lower Molars. At the level of the cusp base but above the cingulid: 0. Absent; 1. Present.
70 - Posterior Lingual Cingulid of Lower Molars: 0. Absent or weak; 1. Distinctive; 2. Strongly developed, crenulated with distinctive cuspules.
71 - Cingular Cuspule e on Lower Molars: 0. Present; 1. Absent.
72 - Cingular Cuspule f on Lower Molars: 0. Absent; 1. Present.
73 - Mesial Transverse Cingulid. 0. Absent; 1. Present as a continuous shelf below the trigonid without occlusal function; 2. Present, a continuous shelf below, having occlusal contact with the upper molar.
74 - Cingulid Shelf Wrapping Around Anterointernal Corner of Lower Molar to Extend to Lingual Side of Trigonid Below Paraconid: 0. Absent; 1. Present, weakly developed restricted to the mesial aspect of the paraconid base; 2. Present, strongly developed, running along most of the lingual base of the paraconid.

75 - Postcingulid: 0. Absent; 1. Present, oblique, and connected to hypoconulid; 2. Present, continue and horizontal above the gum level; 3. Present as a distolabial cingulum, above the gum level.
76 - Interlocking Mechanism Between Two Adjacent Lower Molars: 0. Absent; 1. Present, posterior cingular cuspule d of the preceding molar fits in between cingular cuspules e and f of the succeeding molar or flat surfaces of mesial cingula or cusp b; 2. Present, posterior cingular cuspule d fits between cingular cuspule e and cusp b of the succeeding molar; 3. Present, posterior cingular cuspule d of the preceding molar fits into and embayment or vertical groove of the anterior aspect of cusp b the succeeding molar.
77 - Size Ratio of Posterior Molars. Lower molar preferred when available: 0. Last three postcanines forming a series of posteriorly decreasing size; 1. Penultimate molar is the largest of molars; 2. Ultimate molar is the largest of molars.
78 - Orientation of Paracristid Relative to Longitudinal Axis of Molars: 0. Longitudinal orientation; 1. Oblique; 2. Nearly transverse.
79 - Paraconid Presence on Lower Molars: 0. Present; 1. Absent.
80 - Mesiolingual Surface of Paraconid on Lower Molars: 0. Rounded; 1. Forming a keel.
81 - Procumbent Paraconid on Lower Molars: 0. Absent; 1. Present, projected as a conical cusp beyond crown base.
82 - Proximity Between Paraconid and Metaconid: 0. Bases widely separated; 1. Bases approaching each other becoming confluent; 2. Single cusp (amphyconid).
83 - Molar Trigonid/Talonid Width Ratio: 0. Narrow or absent (talonid <40% of trigonid); 1. Wide (talonid is 40–70% of the trigonid); 2. Talonid is equal or wider than trigonid (above 70% of the width of the trigonid).
84 - Lower Molar Hypoflexid: 0. Absent or shallow; 1. Deep (but less than 50% of the talonid width); 2. Very deep (>60% of the talonid width).
85 - Talonid Basin: 0. Absent; 1. Present.
86 - Morphology of Rear Portion of Molariform: 0. Single cusp (d), cingulum or absent; 1. Present as an incipient heel; 2. Present as a heel (with at least one functional cusp); 3. Present as a transverse V-shaped basin with two major cusps; 4. Rimmed with three major cusps.
87 - Hypoconulid (=Cusp d): 0. Hypoconulid at the cingulid level; 1. Hypoconulid elevated above the cingulid level.
88 - Hypoconid: 0. Absent; 1. Present.
89 - Hypoconulid Orientation: 0. Cusp tip erect or procumbent; 1. Cusp tip recumbent (reclined posteriorly).
90 - Entoconid: 0. Absent; 1. Present but far from hypoconulid, at least equal to one cusp length; 2. Present and twinned with hypoconulid.
91 - Height of Entoconid Compared With Other Talonid Cusps: 0. Lower than the hypoconulid (or even vestigial); 1. Subequal height to the hypoconulid.
92 - Alignment of Paraconid, Metaconid, and Entoconid: 0. Cusps not aligned; 1. Cusps aligned.
93 - Aspect Radio in Occlusal View (Length vs. Width) of Functional Talonid Basin at Cingulid Level: 0. Longer than wide; 1. Length equals width; 2. Wider than long.
94 - Elevation of Talonid: 0. Hypoconulid/protoconid height ratio less than 20% (hypoconulid = cusp d); 1. Hypoconulid/protoconid height ratio between 25% and 35%; 2. Hypoconulid/protoconid height ratio between 40% and 50%; 3. Hypoconulid/protoconid height ratio: 50% or higher.
95 - Width of Upper Molar Stylar Shelf: 0. Present and broad; 1. Present and narrow; 2. Absent.
96 - Labial Cingulum of Upper Molars: 0. Absent or weak; 1. Distinctive cingulum, straight; 2. Distinctive cingulum with strong ectoflexus (but without hypertrophied stylar cusps); 3. Wide cingulum with distinctive ectoflexus, plus individualized and

hypertrophied stylar cusps; 4. Cingulum with distinctive and even-sized multiple cuspules.

97 - Upper Molars With Functional Lingual Protocone That Grinds Against Basin on Lowers: 0. Absent; 1. Present.

98 - Trigon Basin. Protocone must be present: 0. Absent; 1. Present, the labial slope of the protocone determines a basin against the lingual slope of the paracone/metacone.

99 - Transverse Width of Protocone on Upper Molars. Protocone must be present: 0. Narrow (distance from the protocone apex to paracone apex; <0.60 of total tooth width); 1. Strongly transverse (distance from the protocone apex >0.60 of total width).

100 - Anteroposterior Development of Lingual Region on Upper Molars. Protocone must be present: 0. Narrow (anteroposterior distance medial to the paracone; and metacone <0.30 of total tooth length); 1. Moderate development (distance between position of conules = 0.31–0.50 of total tooth length); 2. Long (distance between conules >0.51 of total tooth length).

101 - Conules on Upper Molars: 0. Absent; 1. Present but weak and without cristae; 2. Conules distinctive, with cristae.

102 - Relative Height and Size of Paracone (Cusp b) and Metacone of Upper Molars: 0. Paracone higher and larger than metacone; 1. Metacone is higher and larger than paracone.

103 - Centrocrista Between Paracone and Metacone of Upper Molars. Consider only for tribosphenic forms: 0. Straight; 1. V-shaped, with labially directed postparacista and premetacrista.

104 - Upper Molars Cuspule e: 0. Present; 1. Absent.

105 - Upper Molar Interlock: 0. Absent; 1. Tongue-in-groove interlock.

106 - Central Crest (Medianergrat) in Triangular Upper Molariforms: 0. Absent; 1. Present.

107 - Outline of m1: 0. Oval-shaped; 1. Laterally compressed; 2. Oblong with slight labial bulge; 3. Oblong with strong labial bulge; 4. Triangular or tear-drop shaped; 5. Rectangular or slightly rhomboidal.

108 - Crown Length/Width Ratio Among Lower Molariforms: 0. Crown longer than wide; 1. Crown length/width subequal; 2. Crown wider than long.

109 - Shape of Lingual Margin in Lower Molars: 0. Notched; 1. Flat.

110 - Aspect Ratio of M1: 0. Laterally compressed; 1. Oval-shaped or spindle-shaped; 2. Triangular outline; 3. Dumbbell-shaped; 4. Rectangular or nearly so.

111 - Crown Height Difference Between Buccal and Lingual Sides in Lower Molariforms: 0. Buccal and lingual sides of similar height; 1. Buccal side much taller than lingual side.

112 - Functional Development of Occlusal Facets on Individual Molar Cusps: 0. Absent for lifetime; 1. Absent at eruption but development later by extensive wearing of the crown; 2. Wear facet develops on a morphology approximately present upon eruption.

113 - Topographic Relationships of Wear Facets to Main Cusps: 0. Lower cusps a and c support two different wear facet (1 and 4) that contact the upper main cusp a; 1. Lower cusps a and c support a single wear facet (4) that contacts the upper primary cusp b.

114 - Development and Orientation of Prevallum/Postvallid Shearing: 0. Absent; 1. Present and obtuse; 2. Present, hypertrophied, and transverse.

115 - Upper Molar—Development of Facet 1 and Preprotocrista (or Paracrista) on Upper Molars: 0. Facet 1 (prevallum crest) is short, and does not extend to the stylocone area; 1. Wear facet 1 extends beyond into the hook-like area near the stylocone; 2. Long preprotocrista (below the paracone-stylocone crest) is added to the prevallum shear and extends labially beyond paracone.

116 - Differentiation of Wear Facet 3 and Wear Facet 4: 0. Absent; 1. Present; 2. Facet hypertrophied on the flanks of the strongly V-shaped talonid.
117 - Orientation of Wear Facet 4: 0. Present and oblique to long axis of the tooth; 1. Present and forming a more transverse angle to long axis of the toot.
118 - Wear Pattern on Talonid: 0. Absent; 1. Present.
119 - Direction of Jaw Movement During Occlusion: 0. Dorsomedial movement; 1. Dorsomedial movement with a significant medial component; 2. Dorsoposterior movement.
120 - Metacristid (Protocristid) Orientation on Posterior Molariforms: 0. Parallel to lower jaw axis; 1. Oblique; 2. Transverse.
121 - Bifid Metaconid: 0. Absent; 1. Present.
122 - Bifid Metastyle: 0. Absent; 1. Present.
123 - Distal Metacristid: 0. Present; 1. Absent.
124 - Superficial Features on Occluding Surface of Wear Facets 5 and 6 in Talonid for Basined Talonid: 0. Smooth surface on the talonid (or on cusp d); 1. Multiple ridges within talonid basin.
125 - Position of Stylocone in Posterior Molariforms: 0. Along buccal edge; 1. Separated
126 - Stylocone Relationship in Triangular Teeth: 0. Stylocone connected to paracrista or mesial to its end; 1. Stylocone distal to labial ending of paracrista; 2. Stylocone detached of preparacrista occupying central position on crown.
127 - Stylocone Size in Triangular Teeth: 0. Absent; 1. Small stylar cusp; 2. Prominent cusp subequal or larger than paracone.
128 - Parastylar Hook in Upper Molars: 0. Absent or poorly developed; 1. Present.
129 - Paracone Orientation: 0. Erect; 1. Recumbent; 2. Procumbent.
130 - Metacone: 0. Present; 1. Absent.
131 - Paracone-Metacone Labiolingually Aligned. Consider only in subtriangular upper teeth: 0. Absent, the metacone is labial to paracone; 1. Present, the metacone is aproximately aligned mesiodistally with the paracone.
132 - Accessory Cusps on Bucal Side of Upper Molars: 0. Absent; 1. Present.
133 - Deep Ectoflexus on Upper Molars: 0. Present only on penultimate/ultimate molar; 1. On penultimate and preceding molar; 2. Strongly reduced to absent.
134 - Lower Molariform Roots: 0. Incipient or incomplete separation; 1. Root divided.
135 - Number of Lower Molariform Roots: 0. One; 1. Two; 2. Three or more.
136 - Size of Lower Molar Roots: 0. Subequal; 1. Posterior molar root much smaller; 2. Single root (posterior root absent); 3. Anterior root smaller.
137 - Lower Molar Root Cross Section. m2 considered when available: 0. Circular/subcircular; 1. Anteroposteriorly compressed.
138 - Replacement of at Least Some Functional Molariforms: 0. Present; 1. Absent.
139 - Procumbent and Enlargement of Anteriormost Lower Incisor: 0. Absent; 1. Present, both procumbent and enlarged more than 50% the second.
140 - Trigon Major Axis Orientation. As indicated by the distal wall of the trigonid: 0. Labially; 1. Mesially; 2. Sharply distal.
141 - Lingual Cingulum/Trigon on Paracone. Trigon and/or protocone are considered elaborations of the cingulum: 0. Absent; 1. Present.
142 - Precingulum upper molar: 0. Narrow and closely attached to crown, to absent; 1. Developed forming a lingual cusp.
143 - Postcingulum: 0. Narrow and closely oppresed to crown, to absent; 1. Developed forming a lingual cusp.
144 - Cingula Pre- and Postcingula/Cingulid Height: 0. Absent or little differentiated; 1. Close to crown base; 2. Elevated reaching occlusal surface.
145 - Prehypoconulid Crest. A crest connecting the metaconid with the hypoconulid along the lingual edge of the tooth: 0. Absent; 1. Present.
146 - Number of Upper Molariform Roots: 0. One; 1. Two; 2. Three; 3. More than three.

147 - Position of Lingual Upper Root: 0. Under paracone; 1. Under protocone or trigon.
148 - Supernumerary Roots on Penultimate Upper Premolar: 0. Absent; 1. Present.
149 - Supernumerary Roots on Penultimate Lower Premolar: 0. Absent; 1. Present
150 - Supernumerary Roots on Ultimate Lower Premolar: 0. Absent; 1. Present.
151 - Supernumerary Roots on Ultimate Upper Premolar: 0. Absent; 1. Present
152 - Penultimate Lower Premolar Distal Root: 0. Subequal to mesial root; 1. Large elongated root, more than 50% of crown length.
153 - Lower Molar Contact: 0. Lower molars contact each other somewhere along the mesial and distal edges of the crown; 1. Lower molars do not contact each other being separated by interdental spaces.
154 - Upper Molar Contact: 0. Upper molars extensively contact each other; 1. Upper molars do not contact each other, or barely do so.
155 - Enamel Prism Shape: 0. Prisms absent; 1. Arc; 2. Enclosed.
156 - Enamel Prism Seams: 0. Present; 1. Absent.
157 - Enamel Prism Packing: 0. Hexagonal; 1. Erratic; 2. In rows.
158 - Fusion of Atlas Neural Arch and Intercentrum in Adults: 0. Unfused; 1. Fused.
159 - Atlas Ribs in Adults: 0. Present; 1. Absent.
160 - Fusion of Dens to Axis: 0. Unfused; 1. Fused.
161 - Rib of Axis in Adults: 0. Free ribs present; 1. Ribs fuse to become transverse process.
162 - Postaxial Cervical Ribs in Adult: 0. Free ribs present; 1. Free ribs absent.
163 - Thoracic Vertebrae: 0. 13 thoracic vertebrae; 1. 15 or more thoracic vertebrae.
164 - Lumbar Ribs: 0. Unfused to vertebrae; 1. Synostosed to vertebrae to form transverse processes.
165 - Interclavicle in Adults: 0. Present; 1. Absent.
166 - Contact Relationships in Adults Between Interclavicle and Sternal Manubrium: 0. Posterior end of interclavicle abuts anterior border of manubrium; 1. Interclavicle broadly overlaps the ventral side of the manubrium; 2. Complete fusion of the embryonic membranous and endochondral elements.
167 - Cranial Margin of Interclavicle: 0. Anterior border is emarginated or flat; 1. With a median process (assuming interclavicle is fused to the sternal manubrium in living therians).
168 - Claviculo-Sternal Apparatus Joint: 0. Immobile; 1. Mobile.
169 - Acromioclavicular Joint: 0. Extensive articulation; 1. Limited articulation.
170 - Curvature of Clavicle: 0. Boomerang-shaped; 1. Slightly curved.
171 - Scapula—Supraspinous Fossa: 0. Absent; 1. Weakly developed, present only along a part of the scapula; 2. Fully developed and present along the entire dorsal border of scapula.
172 - Scapula—Acromion Process: 0. Short, (even with or behind the glenoid); 1. Hook-like and extending below the glenoid.
173 - Scapula—Fossa or Process for Teres Major Muscle: 0. Absent; 1. Present.
174 - Procoracoid: 0. Present as a free element; 1. Fused to the sternal apparatus in adult.
175 - Procoracoid Foramen: 0. Present; 1. Absent.
176 - Coracoid: 0. Large, with posterior process; 1. Small, without posterior process.
177 - Manubrium Size Relative to Succeeding Sternebrae: 0. Large; 1. Small.
178 - Orientation of Glenoid Relative to Plane or Axis of Scapula: 0. Nearly parallel to the long axis and facing posterolaterally; 1. Oblique and facing more posteriorly; 2. Perpendicular to the main plane of the scapular plate.
179 - Shape and Curvature of Glenoid: 0. Saddle-shaped, oval, and elongated; 1. Uniformly concave and more rounded in outline.
180 - Medial Surface of Scapula: 0. Concave; 1. Flat.
181 - Humeral Head: 0. Subspherical, weakly inflected; 1. Spherical and strongly inflected.

182 - Intertubercular Groove Separating Deltopectoral Crest From Lesser Tubercle: 0. Shallow and broad; 1. Narrow and deep.
183 - Size of Lesser Tubercle of Humerus: 0. Wider than the greater tubercle; 1. Subequal to narrower than the greater tubercle.
184 - Torsion Between Proximal and Distal Ends of Humerus: 0. Strong (>30); 1. Moderate (30–15); 2. Weak (<15).
185 - Ventral Extension of Deltopectoral Crest or Position of Deltoid Tuberosity: 0. Not extending beyond the midpoint of the humeral shaft; 1. Extending ventrally (distally) past the midpoint of the shaft.
186 - Ulnar Articulation on Distal Humerus: 0. Bulbous ulnar condyle; 1. Incomplete trochlea with vestigial ulnar condyle in anterior view; 2. Trochlea has extending to the anteroventral side.
187 - Radial Articulation on Distal Humerus: 0. Distinct and rounded condyle separated from the ulnar articulation in the anteroventral view of the humerus; 1. Radial articulation forms a rounded condyle anteriorly but its posterior surface is nearly cylindrical; 2. Capitulum, radial articulating structure forms a continuous synovial surface with the ulnar trochlea.
188 - Entepicondyle and Ectepicondyle of Humerus: 0. Robust; 1. Weak.
189 - Rectangular Shelf for Supinator Ridge Extended from Ectepicondyle: 0. Absent; 1. Present.
190 - Styloid Process of Radius: 0. Weak; 1. Strong.
191 - Enlargement of Scaphoid with a Distomedial Projection: 0. Absent; 1. Present.
192 - Size and Shape of Hamate (Unciform) in Wrist: 0. Anteroposteriorly compressed (wider than longer in dorsal view); 1. Mediolaterally compressed (longer than wide).
193 - Acetabular Dorsal Emargination: 0. Emarginated; 1. With a complete rim.
Patagomaia: 1, with a complete rim.
194 - Sutures of Ilium, Ischium, and Pubis Within Acetabulum in Adults: 0. Unfused; 1. Fused.
Patagomaia: 1, fused.
195 - Ischiatic Tuberosity: 0. Dorsal margin with a small or absent ischiatic tuberosity; 1. Dorsal margin concave and ischiatic tubercle hypertrophied.
Patagomaia: 0, absent.
196 - Head of Femur Is Inflected and Set off From Shaft by a Neck: 0. Neck absent, head oriented dorsally; 1. Neck present and head inflected medially.
Patagomaia: 1, neck present and head inflected medially.
197 - Fovea for Acetabular Ligament on Femoral Head: 0. Absent; 1. Present.
Patagomaia: 1, present.
198 - Greater Trochanter: 0. Directed dorsolaterally; 1. Directed dorsally.
Patagomaia: 1, directed dorsally.
199 - Orientation of Lesser Trochanter: 0. On the medial side of the shaft; 1. On the ventromedial or ventral side of the shaft.
Patagomaia: 1, ventromedial.
200 - Size of Lesser Trochanter: 0. Large; 1. Small.
Patagomaia: 1, small.
201 - Patellar Groove of Femur: 0. Absent; 1. Shallow and weakly developed; 2. Well developed.
Patagomaia: 1, shallow and weakly developed.
202 - Proximolateral Tubercle or Tuberosity of Tibia: 0. Large and hook-like; 1. Indistinct.
Patagomaia: 1, indistinct.
203 - Distal Tibial Malleolus: 0. Weak; 1. Distinct.
204 - Fibula Contacting Distal End of Femur: 0. Present; 1. Absent.
Patagomaia: 1, absent.
205 - Distal Fibular Styloid Process: 0. Weak or absent; 1. Distinct.

206 - Fibula Contacting the Calcaneus: 0. Extensive contact; 1. Reduced; 2. Mortise and tenon contact of fibula to the ankle.
207 - Superposition of Astragalus Over Calcaneus: 0. Little or absent; 1. Weakly developed; 2. Present.
208 - Orientation of Sustentacular Facet of Calcaneus with Regard to Horizontal Plane of Astragalus: 0. Nearly vertical; 1. Oblique (<70) to nearly horizontal.
209 - Astragalar Neck: 0. Absent; 1. Weakly developed; 2. Present.
210 - Astragalar Trochlea: 0. Absent; 1. Present.
211 - Calcaneal Tubercle: 0. Short, without terminal swelling; 1. Elongated with distal swelling.
212 - Peroneal Process and Groove of Calcaneus: 0. Forming laterally directed shelf, and without a distinct process; 1. Weakly developed with shallow groove on the lateral side of process; 2. With a distinct peroneal process.
213 - Contact of the Cuboid on Calcaneus: 0. On the anterior end of the calcaneus, the cuboid is aligned with the long axis of the calcaneous; 1. On the anteromedial aspect of the calcaneous, the cuboid is skewed to the medial side of the long axis of the calcaneous.
214 - Relationships of Proximal End of Metatarsal V to Cuboid: 0. Metatarsal V is offset from the cuboid; 1. Metatarsal V is far offset from the cuboid, so that it contacts the calcaneous; 2. Metatarsal V is aligned with the cuboid.
215 - Angle of Metatarsal III to Calcaneus: 0. Metatarsal III is aligned with (or parallel to) an imaginary line through the long axis of the calcaneous; 1. Metatarsal III is oriented oblique to an imaginary line through the long axis of the calcaneous.
216 - Sesamoid Bones in Flexor Tendons: 0. Absent; 1. Present and unpaired; 2. Present and paired.
217 - Tarsal Spur: 0. Absent; 1. Present.
218 - Sharp Constriction of Rostrum in Front of Molariform-Premolar Boundary: 0. Present; 1. Absent.
219 - External Size of Cranial Moiety of Squamosal: 0. Narrow; 1. Broad.
220 - Participation of Cranial Moiety of Squamosal in Braincase: 0. Does not participate in the endocranial wall of the braincase; 1. Participates in the endocranial wall of the braincase.
221 - Neck Between Glenoid and Cranial Moiety of Squamosal: 0. Absent; 1. Present.
222 - External Auditory Meatus: 0. Present as the postcraniomandibular joint sulcus; 1. Absent; 2. Present as a groove on the squamosal, or a notch.
223 - Position of Craniomandibular Joint: 0. Posterior or lateral to the level to the fenestra vestibuli; 1. Anterior to the level of the fenestra vestibuli.
224 - Orientation of Glenoid Fossa: 0. On the inner side of the zygoma and facing ventromedially; 1. On the platform of the zygoma and facing ventrally.
225 - Postglenoid Process: 0. Absent; 1. Present as a distinctive process.
226 - Postglenoid Foramen Within Squamosal Bone: 0. Absent; 1. Present.
227 - Basisphenoid Wing on Ventral Aspect of Skull: 0. Present, overlapping part of or the whole cochlear housing; 1. Absent.
228 - Relationship of Pars Cochlearis to Lateral Lappet of Basioccipital: 0. Pars cochlearis is entirely covered by basioccipital; 1. Pars cochlearis partially covered by basioccipital; 2. Pars cochlearis fully exposed as promontorium.
229 - Medial Flat Facet of Promontorium of Pars Cochlearis: 0. Flat; 1. Inflated and convex.
230 - Ventral Outline and Morphology of Promontorium: 0. Triangular, with steep and slightly concave lateral wall; 1. Elongated and cylindrical petrosal cochlear housing; 2. Bulbous and oval-shaped promontorium.
231 - Cochlea: 0. Short and uncoiled; 1. Elongated and partly coiled; 2. Elongate and coiled to about 360° or more.

232 - Morphology of Internal Acoustic Meatus: 0. The floor is ossified and the meatus is a deep tube; 1. Present as a shallow depression; 2. Present and the floor is developed as the cribriform foramina for auditory nerve.
233 - Primary Bony Lamina Within Cochlear Canal: 0. Absent; 1. Present.
234 - Secondary Bony Lamina for Basilar Membrane Within Cochlear Canal: 0. Absent; 1. Present.
235 - Crista Interfenestralis: 0. Horizontal and extending to base of the paroccipital process; 1. Vertical, delimiting the back of the promontorium.
236 - Postpromontorial Tympanic Recess: 0. Absent; 1. Present.
237 - Caudal Tympanic Process of Petrosal: 0. Absent; 1. Present as a continuous crest; 2. Caudal tympanic process notched.
238 - Prootic Canal: 0. Present; 1. Absent.
239 - Prootic Canal Confluence With Pterygoparoccipital Foramen: 0. Prootic canal present, and its tympanic aperture is a distinct; separate foramen 1. Prootic canal present, and its tympanic aperture is confluent; with the pterygoparoccipital foramen
240 - Lateral Trough Floor Anterior to Tympanic Aperture of Prootic Canal and/or Primary Facial Foramen: 0. Open lateral trough but no bony floor; 1. Present as a bony shelf; 2. Lateral trough absent.
241 - Enclosure of Geniculate Ganglion by Bony Floor of Petrosal: 0. Absent; 1. Present.
242 - Anteroventral Opening of Cavum Epiptericum: 0. Fully open ventrally; 1. Partially enclosed by petrosal or lateral flange; 2. Enclosed by both the alisphenoid and the petrosal
243 - Anterior Lamina of Petrosal and Ascending Process of Alisphenoid and Their Relationships to Exit of Mandibular Branch (V3) of Trigeminal Nerve: 0. V3 foramen placed at the suture of the alisphenoid ascending process and the anterior lamina of petrosal; 1. V3 placed within the enlarged anterior lamina of the petrosal; 2. Double trigeminal foramina within the anterior lamina in addition to the trigeminal foramen at the anterior lamina border with alisphenoid; 3. V3 within the ascending process of the alisphenoid.
244 - Quadrate Ramus of Alisphenoid: 0. Forming a rod overlapping with the anterior part of the lateral flange; 1. Present but not extending back too far, mostly laminar process in the vicinity of the oval foramen; 2. Absent.
245 - Orientation of Anterior Part of Lateral Flange: 0. Horizontal shelf; 1. Ventrally directed; 2. Vestigial or absent.
246 - Vascular Foramen in Posterior Part of Lateral Flange Anterior to Pterygoparoccipital Foramen: 0. Present; 1. Absent.
247 - Relationship of Petrosal Lateral Flange to Crista Parotica: 0. Widely separated; 1. Narrowly separated; 2. Continuous bone formed by petrosal.
248 - Morphology of Pterygoparoccipital Foramen. That is, ramus superior foramen: 0. Laterally open notch; 1. Foramen enclosed by the petrosal or squamosal or both.
249 - Position of Pterygoparoccipital Foramen Relative to Fenestra Vestibuli: 0. Foramen posterior or lateral to the level of the fenestra vestibuli; 1. Foramen anterior to the level of the fenestra vestibuli.
250 - Bifurcation of Paroccipital Process of Petrosal: 0. Absent; 1. Present.
251 - Posterior Paroccipital Process of Petrosal: 0. No ventral projection below the level of its surrounding structures; 1. Projecting below the surrounding structures.
252 - Morphological Differentiation of Anterior Paroccipital Region: 0. Anterior paroccipital region is indistinct from surrounding structures; 1. Anterior paroccipital region is bulbous and distinctive from the surrounding structures; 2. Anterior paroccipital region has a distinct crista parotica.
253 - Epitympanic Recess Lateral to Crista Parotica: 0. Absent; 1. Present.
254 - Relationship of Squamosal on Paraoccipital Process of Petrosal: 0. Squamosal covers the entire paroccipital region; 1. No squamosal cover on anterior

paroccipital region; 2. Squamosal covering a part of the paroccipital region, but not on the crista parotica (the squamosal wall and the cristaparotica are separated by the epitympanic recess).

255 - Medial Process of Squamosal Reaching Toward Foramen Ovale: 0. Absent; 1. Present.

256 - Stapedial Artery Sulcus on Pars Cochlearis of Petrosal: 0. Absent; 1. Present.

257 - Transpomontorial Sulcus for Internal Carotid Artery on Pars Cochlearis: 0. Absent; 1. Present.

258 - Bullar Process of Alisphenoid: 0. Absent; 1. Present.

259 - Hypotympanic Recess in Junction of Alisphenoid, Squamosal, and Petrosal: 0. Absent; 1. Present.

260 - Separation of Fenestra Cochleae from Jugular Foramen: 0. Fenestra cochleae and jugular foramen within the same depression; 1. Separate.

261 - Channel of Perilymphatic Duct: 0. Open channel and sulcus; 1. Channel partially or fully enclosed.

262 - Tensor Tympani Fossa: 0. Indistinct or very shallow; 1. Deep recess on lateral trough anterior to hiatus Fallopii.

263 - Stapedial Muscle Fossa: 0. Absent; 1. Present and in alignment with the crista interfenestralis; 2. Present and lateral to the crista interfenestralis.

264 - Hypoglossal Foramen: 0. Indistinct, either confluent with the jugular foramen or sharing a depression with the jugular foramen; 1. Separated from the jugular foramen.

265 - Shape of Incudo-Mallear Contact: 0. Trochlear surface on the incus; 1. Trough or saddle-shaped contact on the incus; 2. Flat surface.

266 - Incus (Quadrate) Neck: 0. Absent; 1. Present.

267 - Stapedial Process of Incus (Quadrate): 0. Absent; 1. Present.

268 - Dorsal Plate (=Crus Breve) of Incus (Quadrate): 0. Broad plate; 1. Pointed triangle; 2. Reduced.

269 - Incus—Angle of the Crus Breve to Crus Longum: 0. Alignment or obtuse angle between stapedial process (crus longum) and the dorsal plate (crus breve); 1. Perpendicular; 2. Acute angle between the crus breve and crus longum.

270 - Primary Suspension of Incus (Quadrate) on Basicranium: 0. By squamosal and quadratojugal; 1. By squamosal only; 2. By petrosal (either by the preserved direct contact of incus, or by the inference from the presence of a well-defined crista parotica).

271 - Quadratojugal Notch in Squamosal: 0. Present as an independent element in adult; 1. Absent.

272 - Morphology of Stapes: 0. Columelliform-macroperforate; 1. Columelliform-imperforate (or microperforate); 2. Bicrurate-perforate.

273 - Bony Secondary Palate: 0. Ending anterior to the posterior end of the tooth row; 1. Level with the posterior end of the tooth row; 2. Extending posterior to the tooth row.

274 - Relationship of Maxilla to Subtemporal Margin of Orbit: 0. Participating in the rounded subtemporal margin of the orbit; 1. Forming a well-defined edge along the subtemporal margin.

275 - Pterygopalatine Ridge: 0. Present; 1. Absent.

276 - Transverse Process of pterygoid: 0. Present and massive; 1. Present as the hamulus; 2. Greatly reduced or absent.

277 - Palatal Width Anterior to Basisphenoid: 0. Very narrow anterior to the basisphenoid; 1. Intermediate width anterior to the basisphenoid; 2. Palatal width is as broad at the basisphenoid as the internal choanae.

278 - Vault of Nasopharyngeal Passage Near Pterygoid-Basisphenoid Junction: 0. Roof of the pharynx is V-shaped in transverse section, narrowing toward the basisphenoid; 1. Roof of the pharynx is U-shaped in transverse section.

279 - Complete Ossification of Orbital Floor: 0. Absent; 1. Present.

280 - Pattern of Orbital Mosaic as Exposed Externally: 0. Alisphenoid contacts the frontal and parietal; 1. Petrosal anterior lamina contacts the orbitosphenoid thereby separating the alisphenoid from the front and the parietal.
281 - Outline of Facial Part of Lacrimal: 0. Large, triangular, and pointed anteriorly; 1. Small and rectangular or crescentic; 2. Excluded from the facial (and preorbital) part of the skull.
282 - Pila Antotica: 0. Present; 1. Absent (in adult).
283 - Fronto-Parietal Suture on Alisphenoid: 0. Dorsal plate of alisphenoid contacting the frontal by the anterior corner; 1. Dorsal plate of alisphenoid has more extensive contact to the frontal (~50% of its dorsal border).
284 - Jugal on Zygoma: 0. Anterior part of the jugal extends on the facial part of the maxilla and forming a part of the anterior orbit; 1. Anterior part of jugal does not reach the facial part of the maxilla and is excluded from the anterior part of the orbit.
285 - Maximum Vertical Depth of Zygomatic Arch Relative to Length of Skull: 0. Between 10% and 20%; 1. Between 5% and 7%.
286 - Posterior Opening of Post-temporal Canal: 0. At the junction of the petrosal, squamosal, and tabular; 1. Between the petrosal and the squamosal.
287 - Anterior Ascending Vascular Channel for Arteria Diploëtica Magnain Temporal Region: 0. Open groove; 1. Partially enclosed in a canal; 2. Completely enclosed in a canal or endocranial.
288 - Nuchal (Lambdoidal) Crest: 0. Crest overhanging the concave or straight dorsal part of the occipital plate; 1. Weak crest with convex dorsal part of the occipital plate.
289 - Sagittal Crest: 0. Prominently developed; 1. Weakly developed; 2. Absent.
290 - Tabular Bone: 0. Present; 1. Absent.
291 - Shape of Occipital Condyle: 0. Bulbous; 1. Ovoid; 2. Subcylindrical.
292 - Occiput Slope: 0. Occiput slopes posterodorsally, or vertically from the occipital condyles; 1. Occiput slopes anterodorsally from the occipital condyles.
293 - Foramina on Dorsal Surface of Nasal: 0. Absent; 1. Present.
294 - Septomaxilla: 0. Present and with a ventromedial shelf; 1. Present and without the septomaxillary shelf; 2. Absent.
295 - Premaxillary Internarial Process: 0. Present; 1. Absent.
296 - Facial Part of Premaxilla Borders on Nasal: 0. Absent; 1. Present.
297 - Ossified Ethmoidal Cribriform Plate of Nasal Cavity: 0. Absent; 1. Present.
298 - Posterior Excavation of Nasal Cavity Into Bony Sphenoid Complex: 0. Absent; 1. Present, confluent with the nasal cavity; 2. Present and partitioned from the nasal cavity.
299 - External Bulging of Braincase in Parietal Region: 0. Absent; 1. Expanded, the parietal part of the cranial vault is wider than the frontal part, but expansion does not extend to the lambdoidal region; 2. Greatly expanded, expansion of cranial vault extends to lambdoidal region.
300 - Interparietal: 0. Present as a separate element in adult; 1. Absent.
301 - Bony Tentorium Septum: 0. Present; 1. Absent.
302 - Overall Size of Vermis: 0. Small; 1. Enlarged.
303 - Lateral Cerebellar Hemisphere (Excluding Paraflocculus): 0. Absent; 1. Present.
304 - Lateral Extension of Paraflocculus: 0. Less than 30% of total cerebellar width; 1. More than 30% of the cerebellar width.
305 - External Division on Endocast Between Olfactory Lobe and Cerebral Hemisphere (Circular Sulcus): 0. Absent; 1. Present.
306 - Anterior Expansion of Cerebral Hemisphere: 0. Absent; 1. Developed.
307 - Expansion of Posterior Cerebral Hemisphere: 0. Absent; 1. Present.
308 - Interprismatic Matrix: 0. On all sides, widely separated prisms; 1. Distinct inter-row sheets; 2. Prisms "shoulder to shoulder," little interprismatic matrix.
309 - Outer Aprismatic Zone: 0. Present; 1. Absent

310 - Lacrimal Foramen Number: 0. One; 1. Two; 2. None.
311 - Lacrimal Foramen Position: 0. Within orbit; 1. On face.
312 - Preglenoid Process: 0. Absent; 1. Present.
313 - Anterior Lamina of Petrosal: 0. Absent; 1. Present.
314 - Anterior Lamina of Petrosal Contribution to Braincase Wall: 0. Large (present); 1. Small (absent).
315 - Curved Ridge Connecting Caudal Tympanic Process and Crista Interfenestralis: 0. Absent; 1. Present.
316 - "Tympanic Process" of Kielan-Jaworowska: 0. Absent; 1. Present.
317 - Fenestra Vestibuli: 0. Round (stapedial ratio <1.6); 1. Oval (stapedial ratio >1.6).
318 - Presence of labial cuspulids on lower molars: 0. Absent; 1. Present.
319 - Primary trigon and talonid crests: 0. Sharp and continuous; 1. Low and intermittent.
320 - Capacity for embrasure shearing during mastication in molar dentition: 0. Present; 1. None or vestigial.
321 - Metastyle located on discrete projecting lobe in molars: 0. Present; 1. Metastyle not on discrete lobe.

Character list of the Zhou et al.[47] data matrix. The characters scored in *Patagomaia* are indicate in yellow

Mandible

1- Postdentary trough (behind the tooth row): (0) Full presence of the postdentary trough; (1) Reduced postdentary trough in angular region and below dentary peduncle; (2) Absence of the postdentary trough
2- Separate scars for the surangular/prearticular in the mandible: (0) Present; (1) Absent.
3- Overhanging medial ridge above the postdentary trough (behind the tooth row): (0) Present; (1) Absent.
4- Degree of development of Meckel's sulcus: (0) Well developed; (1) Short, and limited to below the mandibular foramen; (2) Vestigial or absent.
5- Curvature of Meckel's sulcus (under the tooth row): (0) Parallel to the ventral border of the mandible; (1) Convergent on the ventral border of the mandible.
6- Groove for Replacement Dental Lamina: 0. Present; 1. Absent
7- Angular process of the dentary: (0) Weakly developed to absent; (1) Present, distinctive but not inflected; (2) Present and transversely flaring (This is different from character state {4} in having a lateral expansion of the angle and in lacking the anterior shelf); (3) Present and slightly medially inflected; (4) Present, strongly inflected, and continuing anteriorly as the mandibular shelf.
8- Position of the angular process of the dentary relative to the dentary condyle: (0) Anterior position (the angular process is below the main body of the coronoid process, separated widely from the dentary condyle); (1) Posterior position (the angular process is positioned at the level of the posterior end of the coronoid process, either close to, or directly under the dentary condyle).
9- Vertical elevation of the angular process of the dentary relative to the molar alveoli: (0) Angular process low, at or near the level of the ventral border of the mandibular horizontal ramus; (1) Angular process high, at or near the level of the molar alveolar line (and far above the ventral border of the mandibular horizontal ramus).
10- Flat ventral surface of the mandibular angle: (0) Absent; (1) Present.
11- Exoflection of the angular process of mandible: (0) Absent; (1) Present.
12- Coronoid bone (or its attachment scar): (0) Present and significant; (1) Vestigial; (2) Absent.

13- Location of the mandibular foramen (posterior opening of the mandibular canal): (0) Within the postdentary trough or in the posterior part of Meckel's sulcus; (1) In the pterygoid fossa and offset from Meckel's sulcus (the intersection of Meckel's sulcus at the pterygoid margin is ventral and posterior to the foramen); (2) In the pterygoid fossa and in alignment with the posterior end of Meckel's sulcus; (3) In the pterygoid fossa but not associated with Meckel's sulcus; (4) Not associated with any of the above structures.
14- Vertical position of the mandibular foramen: (0) Below the alveolar plane; (1) At or above the alveolar plane.
15- Concavity (fossa) for the reflected lamina of the angular bone on the dentary: (0) Present the medial side; (1) Present on the posterior aspect; (2) Absent.
16- Splenial bone as a separate element (as indicated by its scar on the dentary): (0) Present; (1) Absent.
17- Relationship of the "postdentary" complex (surangular-articular-prearticular) to the craniomandibular joint (CMJ) [CMJ is made of several bones in the stem groups of mammals or mammaliaforms, whereas the temporomandibular joint (TMJ) is the medical and veterinary anatomical term applicable to living mammals in which the jaw hinge is made only of the temporal (squamosal) bone and the dentary. CMJ and TMJ are used interchangeably here as appropriate to the circumstances]: (0) Participating in CMJ; (1) Excluded from CMJ.
18- Contact of the surangular bone (or associated postdentary element) with the squamosal: (0) Absent; (1) Present.
19- Pterygoid muscle fossa on the medial side of the ramus of the mandible: (0) Absent; (1) Present.
20- Medial pterygoid ridge (shelf) along the ventral border of the body of the mandible: (0) Absent; (1) Present; (2) Pterygoid shelf present and reaching the dentary condyle via a low crest.
21- Ventral border of the masseteric fossa: (0) Absent; (1) Present as a low and broad crest; (2) Present as a well-defined and thin crest.
22- Crest of the masseteric fossa along the anterior border of the coronoid process: (0) Absent or weakly developed; (1) Present and distinctive; (2) Hypertrophied and laterally flaring.
23- Anteroventral extension of the masseteric fossa: (0) Absent; (1) Extending anteriorly onto the body of the mandible; (2) Further anterior extension below the ultimate premolar/first molar.
24- Labial mandibular foramen inside the masseteric fossa: (0) Absent; (1) Present.
25- Posterior vertical shelf of the masseteric fossa connected to the dentary condyle: (0) Absent; (1) Present, either a thin crest along the curved angular margin of mandible, or a vertically oriented thicker crest, connected to dentary condyle in both configuration.
26- Posterior-most mental foramen: (0) In the canine and anterior premolar (premolariform) region (in the saddle behind the canine eminence of the mandible or behind incisor if canine is absent); (1) Below the penultimate premolar (under the anterior end of the functional postcanine row); (2) Below the ultimate premolar; (3) At the ultimate premolar and the first molar junction; (4) Under the first molar.
27- Articulation of the dentary and the squamosal: (0) Absent; (1) Present, but without condyle/glenoid; (2) Present, with condyle/glenoid.
28- Shape and relative size of the dentary articulation: (0) Condyle small or absent; (1) Condyle massive, bulbous, and transversely broad in its dorsal aspect; (2) Condyle mediolaterally narrow and vertically deep, forming a broad arc in lateral outline, either ovoid or triangular in posterior view.
29- Orientation of the dentary peduncle (condylar process) and condyle: (0) Dentary peduncle more posteriorly directed; (1) Dentary condyle continuous with the semicircular posterior margin of the dentary; the condyle is facing up due to the up-turning of the posterior- most part of the dentary; (2) Dentary articulation extending

vertically for the entire depth of the posterior mandibular ramus; it is confluent with the ramus and without a peduncle; the dentary articulation is posteriorly directed; (3) More vertically directed dentary peduncle.

30- Ventral (inferior) border of the dentary peduncle: (0) Posteriorly tapering; (1) Columnar and with a lateral ridge; (2) Ventrally flaring; (3) Robust and short; (4) Ventral part of the peduncle and condyle continuous with the ventral border of the mandible.
31- Gracile and elongate dentary peduncle: (0) Absent; (1) Present.
32- Position of the dentary condyle relative to the level of the postcanine alveoli: (0) Below or about the same level; (1) Above.
33- Tilting of the coronoid process of the dentary (measured as the angle between the anterior border of the coronoid process and the horizontal alveolar line of all molars): (0) Coronoid process strongly reclined and the coronoid angle obtuse (≥150 degrees); (1) Coronoid process less reclined (135-145 degrees); (2) Coronoid process less than vertical (110-125 degrees); (3) Coronoid process near vertical (95-105 degrees).
34- Gracile base of the coronoid process: (0) Absent; (1) Present.
35- Height of the coronoid process of the dentary: (0) Not reduced; (1) Reduced.
36- Alignment of the ultimate lower molar (or posterior-most postcanine) to the anterior margin of the dentary coronoid process (and near the coronoid scar if present): (0) Ultimate lower molar medial to the coronoid process; (1) Ultimate lower molar aligned with the coronoid process.
37- Direction of lower jaw movement during occlusion (as inferred from teeth): (0) Dorsal movement; (1) Dorsomedial movement with a significant medial component; (2) Dorsoposterior or dorsal-posterior movement.
38- Dentary symphysis: (0) Fused; (1) Unfused.
39- Rostral mandibular spout: (0) Absent; (1) Present.
40- Relative dentary depth in relation to the length: (0) Shallow; (1) Deep.

Premolars

41- Ultimate upper premolar with two rows of multiple cusps: (0) Absent; (1) Present.
42- Upper ultimate and penultimate premolars basined (with main cusps located peripherally surrounding a shallow and broad central basin): (0) absent; (1) present.
43- Upper ultimate and penultimate premolars central valley: (0) the mesial end open; (1) the mesial end closed (trenched when deeply worn).
44- Ultimate upper premolar width relative to the first upper molar: (0) Ultimate upper premolar transversely narrower than, or subequal to, the first upper molar; (1) Ultimate upper premolar transversely wider than the first upper molar; (2) Ultimate upper premolar with a protruding lingual lobe (wider than M1 by about 50% or more).
45- Enamel ridges or flutings on cusps of upper premolars: (0) absent; (1) present.
46- Ultimate upper premolar with multi-rows of cusps - Labial row of cuspules: (0) Absent; (1) Present.
47- Ultimate upper premolar - metastylar lobe: (0) Reduced or absent; (1) Enlarged and wing-like.
48- Ultimate upper premolar - metacone or metaconal swelling: (0) Absent; (1) Present.
49- Ultimate upper premolar - protocone or protoconal swelling: (0) Little or no lingual swelling; (1) Present.
50- Penultimate upper premolar - protocone or protoconal swelling: (0) Little or no lingual swelling; (1) Protoconal swelling; (2) Distinctive and functional protocone.
51- Position the upper premolar with the tallest cusp within the premolar series: (0) No premolar standing out; (1) In ultimate premolar position; (2) In penultimate premolar position.
52- Diastema posterior to the first upper premolar (applicable to taxa with premolar-molar differentiation): (0) Absent; (1) Present.

53- Penultimate upper premolar with multiple cusp-rows: tallest cusp position within longitudinal cusp row: (0) Central; (1) Tallest cusp anterior with posterior cusps (if existing) with decreasing heights; (2) Tallest cusp on buccal row; (3) Cusps of even height.
54- Hypertrophic mesial cusp on ultimate lower premolar: (0) Absent; (1) Present.
55- Ultimate lower premolar - symmetry of the main (middle) cusp a (= protoconid): (0) Asymmetrical (anterior edge of cusp a is more convex in outline than the posterior edge); (1) Symmetrical (anterior and posterior cutting edges are equal or subequal in length; neither edge is more convex or concave than the other in lateral profile).
56- Ultimate lower premolar - anterior cusp b (= paraconid): (0) Absent or indistinctive; (1) Present and distinctive; (2) Enlarged.
57- Ultimate lower premolar - arrangement of principal cusp a, cusp b (if present), and cusp c (assuming the cusp to be c if there is only one cusp behind the main cusp a): (0) Aligned in a single straight line or at a slight angle; (1) Distinctive triangulation; (2) Premolar multicuspate in longitudinal row(s).
58- Ultimate lower premolar – posterior-most (distal) cingulid or cingular cuspule (in addition to cusp c or the metaconid if the latter cusp is present on a triangulated trigonid): (0) Absent or indistinctive; (1) Present; (2) Present, in addition to cusp c or the c swelling; (3) Presence of the continuous posterior (distal) cingulid at the base of the crown.
59- Ultimate lower premolar - outline: (0) Laterally compressed (or slightly angled); (1) Transversely wide (by trigonid); (2) Transversely wide (by talonid); (3) Transversely wide (by inflated anterior cusp and/or distal basined heel).
60- Posterior upper premolar – single enlarged anterior (mesial) sectorial cusp (scored on anterior postcanine in taxa without differentiation of premolars from molars): (0) Absent; (1) Present.
61- Penultimate or ultimate lower premolar with carnassial shearing notch in the middle of the tooth (score on anterior postcanines in taxa where premolars undifferentiated from molars): (0) Absent (single cusp shearing); (1) Present:
62- Lower premolars – basined heel (score on anterior postcanines in taxa in which premolars are not differentiated from molars): (0) Absent; (1) Weakly developed; (2) Full molarization of posterior premolars.
63- Lower premolar: presence of a distinctive distal cingulid with cuspules or crenulated cingulid, and their topographic relation to the main cusp row: (0) Absence of crenulation or cuspules on cingulid row; (1) Present and labially positioned; (2) Present and lingually positioned.
64- Ultimate lower premolar - labial cingulid: (0) Absent or vestigial; (1) Present (at least along the length of more than half of the crown); (2) cuspate distal cingulid.
65- Ultimate lower premolar - lingual cingulid: (0) Absent or vestigial; (1) Present.
66- Ultimate lower premolar - relative height of primary cusp a to cusp c (measured as the height ratio of a and c from the bottom of the valley between the two adjacent cusps): (0) Indistinctive; (1) Posterior cusp c distinctive but less than 30% of the primary cusp a; (2) Posterior cusp c and primary cusp a equal or subequal in height (c is 40%-100% of a).
67- Penultimate lower premolar - paraconid (=cusp b): (0) Absent; (1) Present but not distinctive; (2) Distinctive and slightly enlarged.
68- Penultimate lower premolar - arrangement of principal cusp a, cusp b (if present), and cusp c (we assume the cusp to be c if there is only one cusp behind the main cusp a): (0) Individual cusps in straight alignment (for a tooth with a single cusp, the anterior and posterior crests from the main cusp are in alignment): (1) Cusps in reversed triangulation; (2) With multicusps or multi-serrations in a single longitudinal row; (3) With multicusps or multi-serrations rows.
69- Penultimate lower premolar – labial cingulid: (0) Absent; (1) Present.

70- Gradation of elongation of posterior penultimate premolars: (0) Absent; (1) Present.

Molar Morphology

71- The mesial U-ridge of upper molars with multi-rows of cusps: (0) Absent; (1) Present; (2) Closed by the cuspules.
72- Cuspules and/or transverse fluting of the central basin on upper molars: (0) Absent; (1) Present.
73- Position of cusp A1 on upper molars: (0) A1 is at the same level as B1; (1) A1 is distal to B1.
74- M1 cusp formula (A row relative to B row): (0) 4:4 or lower; (1)5:4; (2) 6:4 or higher.
75- Alignment of the main cusps of the anterior lower molar(s) (justification for separating this feature from the next character on the list): Several taxa of "obtuse-angled symmetrodonts" and eutriconodont amphilestids show a gradient of variation in cusp triangulation along the molar series; the degree of triangulation may be different between the anterior and posterior molars): (0) Single longitudinal row; (1) Reversed triangle–acute (≤90o); (2) Two or more longitudinal multicuspate rows.
76- Triangulation of cusps in the posterior lower/upper molars: (0) Absent; (1) Multi-row and multi-cuspate; (2) Posterior molars slightly triangulated; (3) Posterior molars fully triangulated.
77- B1 cusp on the upper molar (applicable to molars with triangulation): (0) Absent; (1) Present.
78- Postvallum/prevallid shearing (angle of the main trigonid shear facets, based on the second lower molar): (0) Absent; (1) Present, weakly developed, slightly oblique; (2) Present, strongly developed and more transverse; (3) Present, strongly developed, short and slightly oblique.
79- Rank of postvallum shear (on the upper second molar; applicable to molars with reversed triangulation of cusps) (increasing the ranks of postvallum shear and can be ordered):(0) Present but only by the first rank: postmetacrista; (1) Present, with the addition of a second rank (postprotocrista below postmetacrista) but the second rank does not reach labially below the base of the metacone; (2) Metacingulum/metaconule present, in addition to postprotocrista, but the metacingulum crest does not extend beyond the base of the metacone; (3) Metacingulum extended beyond metacone; (4) Metacingulum extended to the metastylar lobe; (5) Second rank postvallum shear forming a broad shelf (as in selenodonty).
80- Postcingulum: (0) Absent or weak; (1) Present; (2) Present and reaching past the metaconule; (3) Formed by the hypoconal shelf raised to near the level of the protocone.
81- Precise opposition of the upper and lower molars: (0) Absent; (1) Present (either one- to-one, or occluding at the opposite embrasure or talonid); (2) Present (one lower molar contacts sequentially more than one upper molar).
82- Relationships between the cusps of the opposing upper and lower molars: (0) Absent; (1) Present, lower primary cusp a occludes in the groove between upper cusps A, B; (2) Present, lower main cusp a occludes in front of the upper cusp B and into the embrasure between the opposite upper tooth and the preceding upper tooth; (3) Present, parts of the talonid occluding with the lingual face (or any part) of the upper molar; (4) Lower multicuspate rows alternately occluding between the upper multicuspate rows; (5) Columnar tooth without cusps and with beveled wear across the entire crown contact surface.
83- Lower m1 with multicuspate rows- lingual row occlude into the basin of upper molar: (0) Absent; (1) Present.

84- Lower m2 with multicuspate rows – the lingual cusp row occlude into the basin of upper molar: (0) Absent; (1) Present.
85- The distal end of lower molars with multi-rows of cusps: (0) Absent; (1) Closed by the ridge;(2) Closed by the cuspules.
86- The cuspules or ridges of the central basin on lower molars: (0) Absent; (1) Present.
87- Fusiform ("spindle-shaped") shearing valley between lingual cusp row and labial cusp row on lower molar: (0) Absent; (1) Present.
88- m1 main lingual row cusp count (distribution revised): (0) 4 or fewer; (1) 5; (2) 6 or more.
89- Lower molars with pyramidal cusps (in contrast to conical cusps): (0) absent; (1) present.
90- Protoconid (cusp a) and metaconid (cusp c) height ratio (on the lower second molar): (0) Protoconid distinctively higher; (1) Protoconid and metaconid nearly equal in height.
91- Relative height and size of the base of the paraconid (cusp b) and metaconid (cusp c) (on the lower second molar): (0) Paraconid distinctively higher than the metaconid; (1) Paraconid and metaconid nearly equal in height; (2) Paraconid lower than metaconid; (3) Paraconid reduced or absent.
92- Elevation of the cingulid base of the paraconid (cusp b) relative to the cingulid base of the metaconid (cusp c) on the lower molars: (0) Absent; (1) Present.
93- Cristid obliqua (or "oblique cristid"): defined as the oblique crest anterior to, and connected with, the labial-most cusp on the talonid heel, the leading edge of facet 3): presence vs. absence and orientation (applicable only to the molar with at least a hypoconid on the talonid or a distal cingulid cuspule): (0) Absent; (1) Present, contact closest to the middle posterior of the metaconid; (2) Present, contact closest to the lowest point of the protocristid; (3) Present, contact closest to the middle posterior of the protoconid.
94- Lower molar - medial and longitudinal crest (='pre-entocristid' or 'pre-hypoconulid') on the talonid heel (only applicable to taxa with talonid or at least a cusp d): (0) Talonid (or cusp d) has no medial and longitudinal crest; (1) Medial-most cristid ('pre-entoconid cristid') of the talonid in alignment with the metaconid or with the post-metacristid if the latter is present (the postmetacristid is defined as the posterior crest of metaconid that is parallel to the lingual border of the crown), but widely separated from the latter; (2) Medial-most cristid of the talonid ('pre-hypoconulid' cristid) is hypertrophied and in alignment with the postmetacristid and abuts the latter by a V-notch; (3) 'Pre-entocristid' crest is offset from the metaconid (and postmetacristid if present), and the 'pre-entocristid' extending anterolingually past the base of the metaconid.
95- Posterior lingual cingulid of the lower molars: (0) Absent or weak; (1) Distinctive; (2) Strongly developed, crenulated with distinctive cuspules (such as the kühneocone).
96- Anterior internal (mesio-lingual) cingular cuspule (e) on the lower molars: (0) Present as an anterior cuspule but not at the cingulid level; (1) Present, at the cingulid level; (2) Present, positioned above the cingulid level; (3) Hypertrophied cusp e = pseudo-hypoconulid; (4) Absent.
97- Anterior and labial (mesio-buccal) cingular cuspule (f): (0) Absent; (1) Present; (2) Hypertrophied to form pseudo-hypoconid.
98- Mesial cingulid features above the gum: (0) Absent; (1) Weak and discontinuous, with individualized cuspules below the trigonid (as individual cuspule e, f, or both, but e and f are not connected); (2) Present, in a continuous shelf below the trigonid (with no relations to the protoconid and paraconid), without occlusal function; (3) Present, with occlusal contact to the upper molar.
99- Crest connecting main cusp a to lingual cingulid cusp g or the cusp g position: (0) Absent; (1) Present.

100- Cingulid shelf wrapping around the anterolingual corner of the molar to extend to the lingual side of the trigonid below the paraconid: (0) Absent; (1) Present, without occlusal function to the upper molars; (2) Present, with occlusal function to the upper molars.
101- Postcingulid (distal transverse cingulid above the gum level) on the lower molars: (0) Absent; (1) Present, horizontal above the gum level.
102- Lower molars interlocking: (0) Absent; (1) Present.
103- Lower molars interlocking - types of interlocking mechanisms: (0) Posterior cingular cuspule d (or the base of the hypoconulid) of the preceding molar fits in between cingular cuspules e and f of the succeeding molar; (1) Posterior cingular cuspule d fits between cingular cuspule e and cusp b of the succeeding molar; (2) Posterior cingular cuspule d or cingulum of the preceding molar fits into an embayment or vertical groove of the anterior aspect of the succeeding molar (without any involvement of distinctive cingular cuspules in interlocking). (3) Anterior corner of succeeding lower molar overlapping posterior corner of preceding lower molar.
104- Size ratio of the last three lower postcanines: (0) Ultimate molar is smaller than the penultimate molar (m1≥m2≥m3; or m2≥m3≥m4; or m3≥m4≥m5; or m4≥m5≥m6; or p4≥m1≥m2); (1) Penultimate molar is the largest of the molars (m1≤m2≤m3≥m4; or m1≤m2>m3); (2) Ultimate molar is larger than the penultimate molar (m1≤m2≤m3); (3) Equal size.
105- Paraconid position relative to the other cusps of the trigonid on the lower molars (based on the lower second molar): (0) Paraconid in anterolingual position; (1) Paraconid lingually positioned (within lingual 1/4 of the trigonid width); (2) Paraconid lingually positioned and appressed to the metaconid; (3) Paraconid reduced in the selenodont/lophodont patterns.
106- Orientation of the paracristid (or the crest between cusps a and b) relative to the longitudinal axis of the molar (This is separated from the previous character [“lingual” vs. “labial” position of the paraconid] because of the different distribution of the a-b crest among mammals with non-triangulated molars sampled here): (0) Longitudinal orientation; (1) Oblique; (2) Nearly transverse.
107- Angle of the paracristid (b-a crest) and the protocristid (a-c crest) on the lower molar: (0) > 90 degrees; (1) 90 ~ 50 degrees; (2) < 35 degrees.
108- Mesiolingual vertical crest of the paraconid on the lower molars (applicable only to taxa with reversed triangulation of the molar cusps): (0) Rounded; (1) Forming a keel.
109- Anteroposterior shortening at the base of the trigonid relative to the talonid (applicable only to taxa with a talonid heel with a distal cusp d; measured at the lingual base of the lower second molar trigonid where possible): (0) Trigonid long (extending over 3/4 of the tooth length); (1) Swelling on the side walls of the trigonid (taxa assigned to this character state have a trigonid length ratio 45%~50%; but their morphology is different from all other states in that their side walls are convex); (2) No shortening (trigonid 50-65% of tooth length); (3) Some shortening (the base of trigonid < 50% of tooth length); (4) Anteroposterior compression of trigonid (trigonid 40~45% of the tooth length).
110- Molar (the lower second molar measured where possible) trigonid/talonid heel width ratio: (0) Narrow (talonid ≤40% of trigonid); (1) Wide (talonid is 40-70% of the trigonid in width); (2) Talonid is equal or wider than trigonid.
111- Lower molar hypoflexid (concavity anterolabial to the hypconid or cusp d): (0) Absent or shallow (all "triconodont-like" teeth are coded as "0" here as long as they have cuspule d); (1) Deep (40~50% of talonid width); (2) Very Deep (>65%).
112- Morphology of the talonid (or the posterior heel) of the molar: (0) Absent; (1) Present, as an incipient heel, a cingulid, or cingular cuspule (d); (2) Present, as a transverse ‘V- shaped’ basin with two functional cusps; (3) Present, as an obtuse ‘V-shaped’ triangle; (4) Present as a basin (rimmed with 3 functional cusps with at

leas is a functional crest to define the medial rim of the basin if the entoconid is not already present) with wear occurs only crests but absent from the bottom of the basin; (5) As a functional basin (rimmed by 3 cusps) with wear occurs inside the basin.

113- Hypoconid (we designate the distal cingulid cuspule d as the homolog to the hypoconid in the teeth with linear alignment of the main cusps; we assume the cusp to be the hypoconid if there is only a single cusp on the talonid in the teeth with reversed triangulation):(0) Present, but not elevated above the cingulid level; (1) Present (as distal cusp d), elevated above the cingulid level, labially positioned (or tilted in the lingual direction); (2) Present (larger than cusp d, with occlusal contact to the upper molar), elevated above the cingulid level, labially positioned.

114- Hypoconulid (if there are only two functional cusps on the talonid, we assume that the second and more lingual cusp on the talonid to be the hypoconulid): (0) Absent; (1) Present, and median (near the mid-point of the transverse talonid width); (2) Present, and placed within the lingual 1/3 of the talonid basin; (3) Incorporated into the crest of lophodont or selenodont conditions.

115- Anterior lower molar (preferably the first, or the second if the first is not available) - hypoconulid - anteroposterior orientation: procumbent vs. reclined (applicable to the taxa with at least two cusps on the talonid): (0) Cusp tip reclined and the posterior wall of the hypoconulid is slanted and overhanging the root; (1) Cusp tip procumbent and the posterior wall of the cusp is vertical; (2) Cusp tip procumbent and the posterior wall is gibbous.

116- Hypoconulid labial postcingulid (shelf) on the lower molars (non-homologous with the postcingulid coded elsewhere in this list because of the different relationship to the talonid cusps; applicable to taxa with identifiable hypoconid and hypoconulid only): (0) Absent; (1) Present as a crest descending mesiolabially from the apex of the hypoconulid to the base of the hypoconid.

117- Last lower molar - hypoconulid - orientation and relative size (applicable to the taxa with at least a talonid heel; scored on the third molar for *Peramus* and eutherians, the fourth molar for *Kielantherium* and metatherians; justification for separating this character from the character of the anterior molar hypoconulids is that the ultimate molar shows different morphology and distribution, especially in taxa in which there is a posteriorly decreasing size gradient, e.g. *Deltatheridium*): (0) Short and erect; (1) Tall (higher than hypoconid) and recurved.

118- Entoconid (if there are three functional cusps on the talonid, we assume that the third and lingual-most functional cusp on the talonid is the entoconid): (0) Absent; (1) Present, about equal distance to the hypoconulid as to the hypoconid; (2) Present, with slight approximation to the hypoconulid (distance between the hypoconulid and entoconid noticeably shorter than between the hypoconulid and hypoconid); (3) Present, and twinned with the hypoconulid.

119- Height ratio of the medial side of the crown (apex of the hypoconid to the base of the labial crown) vs. the most lingual cusp on the talonid to the base of the labial crown (this character can be based either on the entoconid if the entoconid is present or the hypoconulid if the entoconid cannot be scored): (0) Entoconid absent on the talonid heel; (1) Entoconid lower than the hypoconid; (2) Entoconid near the height of the hypoconid; (3) Entoconid near the height of the hypoconid and linked to the hypoconid by a transverse crest.

120- Alignment of the paraconid, metaconid, and entoconid on the lower molars (applicable only to taxa with triangulation of the trigonid cusps and the entoconid present on the talonid): (0) Cusps not aligned; (1) Cusps aligned.

121- The length vs. width ratio of the functional talonid basin of the lower molars (in occlusal view, measured at the cingulid level, and based on the second molar): (0) Longer than wide (or narrows posteriorly); (1) Length equals width; (2) Wider than long.

122- Elevation of the talonid (measured as the height of the hypoconid from the cingulid on the labial side of the crown) relative to the trigonid (measured as the height of protoconid from the cingulid) (applicable only to the teeth with reversed triangulation): (0) Hypoconid/protoconid height ratio less than 20% (hypoconid or cusp d is on the cingulid); (1) Hypoconid/protoconid height ratio between 25% and 35% (talonid cusp elevated above the cingulid level); (2) Hypoconid/protoconid height ratio between 40% and 60%; (3) Hypoconid/protoconid height ratio between >60% and 80%; (4) Equal height.
123- Size (labiolingual width) of the upper molar labial stylar shelf on the penultimate molar: (0) Absent; (1) Present and narrow; (2) Present and broad.
124- Presence vs. absence of the ectoflexus on the upper second molar (or postcanines in the middle portion of the postcanine row). Comments: justification for separating this character from the next is that only a single upper molar is known for three taxa that are otherwise crucial for assessing the timing and biogeography of the divergence of earliest-known crown therians: *Murtoilestes, Atokatheridium*, and *Kokopellia. Nanolestes* and *Shuotherium* are also only represented by isolated upper molars. Therefore, the gradient character of the ectoflexus along the tooth row is not applicable for these taxa. Presence vs. absence of the ectoflexus alone does not exhaust the systematic distribution of the ectoflexus-related characters among taxa with isolated upper molars. (0) Absent or weakly developed; (1) Present.
125- Ectoflexus gradient along the molar series (see the above for justification of separating presence/absence from the gradient of the ectoflexus on the upper molar(s)): (0) Present on penultimate molar, but weakly developed or absent on the anterior molars; (1) Present on the penultimate and preceding molars.
126- Morphological features on the labial cingulum or stylar shelf of the upper molars (excluding the parastyle and metastyle): (0) Indistinctive; (1) Distinctive cingulum, without cuspules; (2) Individualized or even hypertrophied cuspules; (3) W-pattern on stylar shelf; (4) Cingulum crenulated with distinctive and even-sized multiple cuspules.
127- Distinctive lingual cingulum on upper molariforms: (0) Absent; (1) Present.
128- Upper molar protocone: (0) Functional cusp and lingual swelling absent; (1) Functional cusp absent, but the lingual side is more swollen than the labial side at the cingular level; (2) Functional cusp present.
129- Degree of labial shift of the protocone (distance from the protocone apex to the lingual border vs. the total tooth width, in %) (applicable only to those taxa with reversed triangulation): (0) Protocone present but no labial shift (10%-20%); (1) Moderate labial shift (25%-30%); (2) Substantial labial shift (≥ 40%).
130- Morphology of the protocone (applicable only to those taxa with reversed triangulation and a lingual swelling of the upper molar): (0) Protoconal region present but no distinct protocone; (1) Protocone present, its apical portion anteroposteriorly compressed; (2) Apical portion slightly expanded; (3) Apical portion expanded; (4) Apical portion forming an obtuse triangle with the protoconal cristae.
131- Height of the protocone/pseudoprotocone relative to the paracone and metacone (whichever is higher of the latter two): (0) Protocone/pseudoprotocone markedly lower (less than 70%); (1) Protocone of intermediate height (70%~80%); (2) Protocone/pseudoprotocone near the height of paracone and metacone (within 80%).
132- Height and size of upper molar cusp B and cusp C of triconodont-type molariform (based on the upper second molar if available): (0) Paracone noticeably higher and larger at the base than metacone; (1) Paracone and metacone of equal size or paracone, or sub-equal.
133- Height and size of the paracone and metacone (applicable only to molars with cusps of triangular arrangement; based on the upper second molar if available): (0)

Paracone noticeably higher and larger at the base than metacone; (1) Paracone slightly larger than metacone; (2) Paracone and metacone of equal size or paracone lower than metacone.

134- Metacone position relative to paracone: (0) Metacone labial to par cone; (1) Metacone about the same level as paracone; (2) Metacone lingual to paracone.

135- Base of the paracone and metacone (based on the upper second molar if available, applicable only to triangulated molars): (0) Merged; (1) Separated.

136- Centrocrista between the paracone and the metacone of the upper molars (applicable only to taxa with well-developed metacone and distinctive wear facets 3 and 4): (0) Straight; (1) V-shaped, with labially directed postparacrista and premetacrista.

137- Anteroposterior width of the conular region (with or without conules) on the upper molars (applicable only to taxa with reversed triangulation and an occluding lingual portion of the upper molar; for the taxa with conules, this is measured between the paraconule and metaconule; for those taxa without conules, this is measured as the length of the tooth medial to the base of paracone; the upper second molar measured where possible): (0) Narrow (anteroposterior distance medial to the paracone and metacone less than 0.30 of total tooth length); (1) Moderate development (distance between position of conules = 0.31—0.50 of total tooth length); (2) Wide (distance between conules greater than 0.51 of total tooth length); (3) Expanded.

138- Presence of the paraconule and metaconule on the upper molars: (0) Absent; (1) Present.

139- Relative position of the paraconule and metaconule on the upper first and second molars: (0) Paraconule and metaconule closer to the protocone; (1) Both positioned near the midpoint of the protocone-metacone; (2) Paraconule and metaconule labial to the midpoint.

140- Internal conular cristae (conular wing): (0) Cristae indistinctive; (1) Cristae distinctive and wing-like.

141- Parastylar groove (on upper second molar): (0) Weak or absent; (1) Moderately to well developed.

142- Stylar cuspule “A”, the parastyle, on the upper molars (of the Bensley-Simpson system; cuspule “E” of the Crompton designation for triconodontan-like molariform): (0) Present (at least a swelling is present); (1) Absent.

143- Preparastyle on the upper first molar (applicable to molars with triangulation): (0) Absent; (1) Present.

144- Stylar cuspule “B” (opposite the paracone) (based on the upper second molar if available): (0) Vestigial to absent; (1) Small but distinctive; (2) Large, or slightly larger than the parastyle. Note: In some “symmetrodont” mammals, an extra cuspule "B-1" can be present in the upper molar. This can be potentially added to be a further character state, cuspule in addition to cusp "B."

145- Stylar cuspule "C" (near the ectoflexus) on the penultimate upper molar: (0) Absent; (1) Present.

146- Stylar cuspule "D" (opposite the metacone) on the penultimate upper molar: (0) Absent; (1) Present.

147- Absence vs. presence and size of the stylar cuspule “E” (Bensley-Simpson designation; not the Crompton cusp E): (0) Absent or poorly developed; (1) Present, less developed than or subequal to stylar cuspule “D”; (2) Present and better developed than cuspule “D”.

148- Position of the stylar cuspule “E” relative to cusp “D” or “D-position”: (0) “E” more lingual to “D” or “D-position”; (1) “E” distal to or at same level as “D” or “D-position”.

149- Size and labial extent of the metastylar lobe and parastylar lobe (based on the upper first molar if available; if not, then based on upper second): (0) Metastylar lobe smaller than the parastylar lobe; (1) Metastylar lobe of similar size and labial extent

to the parastylar lobe; (2) Metastylar lobe much larger than the parastylar lobe; (3) Metastylar lobe absent.

150- Salient postmetacrista on the upper molars (applicable to taxa with reversed triangulation): (0) Absent or weakly developed; (1) Well-developed but no longer than the metacone-protocone distance; (2) Hypertrophied and longer than the metacone-protocone distance.

151- Selenodont molar pattern: (0) Absent; (1) Present.

152- Outline of the lower first molar crown (in crown view): (0) Laterally compressed; (1) Oblong with slight labial bulge; (2) Triangular or tear-drop shaped; (3) Rectangular (or rhomboidal); (4) Oval shaped; (5) Circular.

153- Outline of the lower second molar crown (in crown view): (0) Laterally compressed; (1) Oblong with slight labial bulge; (2) Triangular or tear-drop shaped; (3) Rectangular (or rhomboidal); (4) Circular.

154- Aspect ratio and outline of the upper first molar: (0) Laterally compressed; (1) Longer than transversely wide (oval-shaped or spindle shaped); (2) Transversely wider than long (triangular outline); (3) Rectangular or nearly so; (4) Oval shaped; (5) Circular.

155- Carnassial shearing blades on posterior aspect of the ultimate upper premolar and anterior aspect of the first lower molar: (0) Absent; (1) Present.

156- Upper molar interlock: (0) Absent; (1) Present.

157- Anterior molar(s) - types of upper molar interlock: (0) Notch interlock (with cingular cusps involved or without); (1) Tongue-in-groove interlock; (2) Parastylar lobe of a succeeding molar lumbricated with the metastylar region of a preceding molar.

158- Posterior upper molar(s) - types of upper molar interlock: (0) Posterior end of preceding molar imbricating anterolabial side of ultimate upper molar; (1) Parastylar lobe of a succeeding molar imbricated with the metastylar region of a preceding molar: cladotherians; boreosphenidans; (2) Tongue-in-groove interlock.

159- Wear facets on the lingual side of lingual cusps of m1: (0) Absent; (1) Present.

160- Wear facets on buccal side of M2: (0) On all buccal cusps; (1) On buccal side of A1,but not on the buccal side of the mesiobuccal cusp.

161- Functional development of occlusal facets on individual molar cusps: (0) Absent; (1) Absent at eruption but developed later by crown wear; (2) Wear facets match upon tooth eruption (inferred from the flat contact surface upon eruption).

162- Topographic relationships of wear facets to the main cusps: (0) Wear pattern across the entire crown; (1) Lower cusps a, c support two different wear facets (facets 1 and 4) that contact the upper primary cusp A; (2) Lower cusps a, c support a single wear facet (facet 4) that contacts the upper primary cusp B (this facet extends onto cusp A as wear continues, but 1 and 4 do not develop simultaneous in these taxa); (3) Multicuspate series, each cusp may support 2 wear facets.

163- Development and orientation of prevallum/postvallid shearing (based on either upper or the lower molar structures): (0) Absent; (1) Present and obtuse; (2) Present, hypertrophied and transverse.

164- Wear facet 1 (a single facet supported by cusp a and cusp c) and facet 2 (a single facet supported by cusp a and cusp b): (0) Absent; (1) Present.

165- Upper molars - development of facet 1 and the preprotocrista (applicable to molars with reversed triangulation): (0) Facet 1 (prevallum crest) short, not extending to the stylocone area; (1) Facet 1 extending into the hook-like area near the stylocone; (2) Preprotocrista long, extending labially beyond the paracone.

166- Differentiation of wear facet 3 and facet 4 (applicable to taxa with a distal cusp d or “hypoconulid”): (0) Absent; (1) Present; (2) Facets 3 and 4 hypertrophied on the flanks of the strongly V-shaped talonid.

167- Orientation of facet 4 (on the posterior aspect of the hypoconid): (0) Present and oblique to the long axis of the tooth; (1) Present and forming a more transverse angle to the long axis of the tooth.

168- Morphology of the posterolateral aspect of the talonid (the labial face of the hypoconid or equivalent area of Crompton facet 4, applicable to taxa with fully basined talonid): (0) Gently rounded; (1) Angular.
169- Wear pattern within the talonid basin (applicable to those taxa with triangulated molars): (0) Absent; (1) Present; (2) Present apically on the crests of the talonid; (3) Apical wear on crest and lophodont.
170- Development of the distal metacristid (applicable only to taxa with reversed triangulation): (0) Present; (1) Absent.
171- Differentiation of wear facets 5 and 6 on the labial face of the entoconid: (0) Absent;(1) Present.
172- Surficial features on the occluding surfaces on the talonid (only applicable to taxa with reversed triangulation): (0) Smooth surface on the talonid heel (or on cusp d); (1) Multiple ridges within the talonid basin; (2) Talonid present, but wear occurs apically on the crests of cristid obliqua and hypoconid cristid (V-shaped talonid crests).
173- Molar wear facets pseudo-3 and pseudo-4: (0) Absent; (1) Present.
174- Molar wear facets pseudo-5 and pseudo-6: (0) Absent; (1) Present.
175- Pseudo-cusp e and f hypertrophied: (0) Absent; (1) Present.
176- Medio-lateral compression of the base of cusp a (or protoconid) (applicable only to "triconodont"-like, "symmetrodont"-like, or generalized tribophenic teeth):(0) Absent (base of cusp a width is 40% or more of tooth length, measured on m1); (1) Present (base of cusp a width≥ 30% of tooth length, measured on m1 or other anterior molariforms).

Other Dental Features

177- Number of upper incisors: (0) Five; (1) Four; (2) Three; (3) Two; (4) One; (5) No incisors.
178- I2 enlargement: (0) absent; (1) present.
179- Number of cusps on posterior upper incisors: (0) One; (1) two or more.
180- Number of lower incisors: (0) Five or more; (1) Four; (2) Three; (3) Two; (4) One; (5) No incisors.
181- Lower anterior-most incisor enamel: (0) Covers the whole incisor; (1) Restricted anteriorly.
182- Lower anterior-most incisor with open root: (0) Absent; (1) Present.
183- Upper anterior-most incisor enamel: (0) Covers the whole incisor; (1) Restricted anteriorly.
184- Upper anterior-most incisor with open root: (0) Absent; (1) Present.
185- Upper canine - presence vs. absence, and size: (0) Present and enlarged; (1) Present and small; (2) Absent.
186- Upper canine – number of cusps: (0) Peg-like with single cusp; (1) Two or more cusps.
187- Number of upper canine roots: (0) One; (1) Two.
188- Lower canine - presence vs. absence and size: (0) Present and enlarged; (1) Present and small; (2) Absent.
189- Number of lower canine roots: (0) One; (1) Two.
190- Orientation of lower canine: (0) Erect; (1) Procumbent.
191- Number of upper premolars (only applicable to taxa with premolar vs. molar differentiation): (0) Five or more; (1) Four; (2) Three; (3) Two or less.
192- Number of lower premolars: (0) Five or more; (1) Four; (2) Three; (3) Two or less.
193- Number of lower molars or molariform postcanines: (0) Six or more; (1) Five; (2) Four; (3) Three; (4) Two or less.
194- Number of upper molars or molariform postcanines (applicable only to those taxa that do not have multiple dental replacements): (0) Six or more; (1) Five; (2) Four; (3) Three; (4) Two or less.

195- Total number of upper postcanine loci: (0) More than 8 (including the loci plus the alveoli of shed anterior postcanines); (1) Eight; (2) Seven, (3) Six; (4) Five or less.
196- Total number of lower postcanine loci: (0) Eight or more; (1) Seven; (2) Six; (3) Five or fewer.
197- Procumbency and diastema of first (functional) upper premolar or postcanine in relation to the upper canine: (0) Not procumbent and without diastema; (1) Procumbent and with diastema.
198- Diastema separating the lower first and second premolars (defined as the first and second functioning premolar or premolariform postcanine): (0) Absent (gap less than one tooth root for whichever is smaller of the adjacent teeth); (1) Present, subequal to one tooth-root diameter or more; (2) Present, equal to or more than one-tooth length.
199- Ultimate lower premolar bladed or crenulated: (0) Absent; (1) Present.
200- Upper anterior-most incisor (I1): (0) Subequal to the remaining incisors, no diastema with the second incisor; (1) Anteriorly projecting, separated from the second incisor (or any following teeth if posterior incisors are absent) by a diastema; (2) Absent (as evidenced by a small median gap between the mesial-most incisors).
201- Ultimate and penultimate upper incisors- morphology: (0) Peg-like/conical; (1) Present, and spoon-shaped to rhomboid-shaped in lateral view; (2) Present, and spatulate in lateral view; (3) Ultimate and/or penultimate upper incisors bicuspate or tricuspate.
202- Staggered lower incisor: (0) Absent; (1) Present.
203- Replacement pattern of incisors and canines: (0) More than one replacement; (1) One replacement; (2) No replacement.
204- Replacement of at least some posterior functional molariform postcanines: (0) Present; (1) Absent.
205- Enlargement of the lower anterior-most incisor: (0) Absent; (1) Present (at least 50% longer than the adjacent incisor).
206- Enlarged diastema in the lower incisor-canine region (better developed in older individuals): (0) Absent; (1) Present and behind the canine; (2) Present and behind the posterior incisor.
207- U-shaped transverse ridge in the lower multi-rowed molars: (0) Absent; (1) Present, at second anterior cusp: (2) Present, at the anterior rim.
208- Fusuliform (“spindle-shaped”) shearing valley on anterior upper molars: (0) Absent; (1) Present.
209- Cusp ratio on lingual row of multi-rowed lower molar: (0) Cusps are of subequal height; (1) Mesial cusp on the lingual row the highest.
210- Inflated hook cusp (hypertrophied and recurved) at the mesiolabial end of lower molars (to fit into the fusuliform valley of upper molars). (0) Absent; (1) Present.
211- Cusp ratio on buccal row of multi-rowed lower molar: (0) All cusps are of equal height; (1) The middle cusps higher than the mesial and distal cusps.
212- Enlarged and more centrally placed second cusp of lingual row on lower m1 (applicable only to molars with multi-rows of multiple cusps): (0) Absent; (1) Present.
213- Upper premolar/molar with multi-cusped rows - cusp ratio in the labial row of multi-cusp row on ultimate upper molar: (0) Distal cusp highest, with a gradient of anteriorly decreasing height; (1) Cusps in same row of equal height; (2) Mesial cusp is slightly higher than distal cusp.
214- Antero-lingual wing (in addition to two main cusp rows) on M1: (0) Absent; (1) Present.
215- Last (ultimate) upper molar - alignment of multi-cusped rows: (0) Absence of lingual offset of ultimate molar to penultimate molar; (1) Presence of offset of ultimate molar from the penultimate molar: the lower ultimate molar lingual row

occludes with the lingual side of the upper second labial row, or the labial side of the lower ultimate molar occluding with the labial side of the upper ultimate molar.

216- Complete middle valley between lingual cusp row and labial cusp row on lower m2: (0) Absent; (1) Present.

217- Multi-rowed ultimate lower molar, row length difference: (0) Labial cusp row about equal as lingual cusp row; (1) Labial row shorter at the anterior end (by at least half-cusp length) than lingual row; (2) Labial row longer at the posterior end than lingual row (by at least half-cusp length).

218- Enamel microstructure: (0) Synapsida columnar enamel (prismless); (1) ‘Transitional’ (sheath indistinct, ‘prismatic’ crystallites inclined at less than 45o to the ‘interprismatic’ matrix); (2) Full prismatic enamel; (3) Enamel absent.

219- Hypsodonty roots of cheek teeth: (0) Absent; (1) Present.

220- Open root end of the postcanines: (0) Absent; (1) Present.

221- Degrees of postcanine root division: (0) Single root; (1) divided roots connected by dentine sheets; (2) two or three complete divided roots or more; (3) multiple roots coalesced.

222- Orientation of the crown-root of upper molariform row in transverse (coronal) section: (0) Vertical; (1) Oblique.

Vertebrae and Ribs

223- Fusion of the atlas neural arch and intercentrum: (0) Absent; (1) Present.

224- Atlas: fusion of half-neural arches at dorsal midline: (0) Absent; (1) Present.

225- Atlas rib: (0) Present; (1) Absent.

226- Fusion of dens to the axis: (0) Absent; (1) Present.

227- Axis rib: (0) Present; (1) Absent (rib fused to form the transverse process). Note: the base for the axial rib is represented by a stump.

228- Inferior lamina (“tuberculi anterior”) on the centra of posterior cervicals: (0) Absent; (1) Present.

229- Postaxial cervical ribs: (0) Unfused; (1) Fused.

230- Number of dorsal vertebrae bearing ribs: (0) 13 or less; (1) 14 or more.

231- Overlapping ventral costal plates: (0) Absent; (1) Present.

232- Overlapping lumbar or posterior thoracic ribs: (0) Present; (1) Absent.

233- Anticlinal vertebra: (0) Absent; (1) Present.

234- Anticlinal vertebra position (not applicable for vertebral column without an anticlinal vertebra): (0) Anticlinal absent; (1) More posterior position (within last 4 lumbar vertebrae); (2) Anteriorly positioned (within the anterior 13 dorsal and the thoracic vertebral region if thoraco-lumbar boundary is distinctive):

235- Mobile lumbar ribs: (0) Present; (1) Absent.

236- Orientation of lumbar ribs or transverse processes: (0) Posterolaterally directed; (1) Laterally or anterolaterally directed.

237- Xenarthrous articulation in addition to the pre- and post-zygapophyses of lumbar vertebrae: (0) Absent; (1) Present.

238- Expanded dorsal end (“flat top”) of neural spine of posterior dorsal vertebrae: (0) Absent; (1) Present.

Shoulder Girdle

239- Interclavicle: (0) Present; (1) Absent.

240- Contact relationships between the interclavicle (embryonic membranous element) and the sternal manubrium (embryonic endochondral element): (0) Two elements distinct from each other, posterior end of the interclavicle abuts with the anterior border of manubrium; (1) Two elements distinct from each other, the interclavicle broadly overlaps the ventral side of the manubrium; (2) Complete fusion of the embryonic membranous and endochondral elements resulting in a single and enlarged manubrium.

241- Inverclavicle distal expansion: (0) Absent; (1) Present.

242- Cranial margin of the interclavicle/manubrium (assuming the interclavicle is fused to the sternal manubrium in living therians): (0) Emarginated or flat; (1) With a median process.
243- Interclavicle to sternal manubrium length ratio: (0) Interclavicle twice the length of manubrium; (1) Interclavicle nearly equal to manubrium in length.
244- Sternoclavicular joint (assuming that homologous elements of the interclavicle and the manubrium are fused to each other in therians): (0) Immobile; (1) Mobile.
245- Interclavico-manubrial craniolateral process: (0) Absent; (1) Present.
246- Acromioclavicular joint: (0) Extensive articulation; (1) Limited articulation (either pointed acromion, pointed distal end of clavicle, or both).
247- Curvature of the clavicle: (0) Boomerang-shaped; (1) Slightly curved.
248- Clavicle - lateral (distal) end expanded with helical articular surface: (0) Tapering or truncated; (1) Expanded with helical articular surface.
249- Scapula - supraspinous fossa: degree of development along the length: (0) Present only in the "acromional region" of the scapula, and on the cranial (dorsal) border of the scapula and positioned anterior to the glenoid); (1) Weakly developed (present only along a part of the scapula and positioned lateral to the glenoid); (2) Fully developed (present along the entire dorsal border of the scapula).
250- Proportion of supraspinous vs. infraspinous fossae (width measured across the "saddle region" of the spine, or near the mid-length of the scapula): (0) Supraspinous "fossa" on the cranial aspect of the scapula and much narrower than infraspinous fossa; (1) Supraspinous width is 50% to 80% that of infraspinous fossa; (2) Fossae subequal; (3) Supraspinous over 150% that of infraspinous fossa.
251- Scapula - acromion process: (0) Short stump, level with or behind the glenoid; (1) Elongate and extending below the glenoid; (2) Pointed process, oriented anteriorly.
252- Scapula - a distinctive fossa for the teres major muscle on the lateral aspect of the scapular plate: (0) Absent; (1) Present.
253- Procoracoid: (0) Present and distinct; (1) Fused to the sternal apparatus.
254- Procoracoid foramen: (0) Present; (1) Absent (assuming the procoracoid is fused to the sternal apparatus in living therians).
255- Coracoid: (0) Large, with posterior process; (1) Small, without posterior process.
256- Anterior process of the coracoid: (0) Indistinctive; (1) Distinctive; (2) Distinctive and forming a broad plate.
257- Coracoid process bridging over posteriorly toward the vertebral border of scapula (or fused with the latter): (0) Absent; (1) Present.
258- Size of the anterior-most element ('manubrium') relative to the subsequent sternebrae in the sternal apparatus: (0) Large; (1) Small.
259- Orientation ('facing' of the articular surface) of the glenoid (relative to the plane or the long axis of the scapula): (0) Nearly parallel and facing posterolaterally; (1) Oblique and facing more posteriorly; (2) Perpendicular.
260- Shape and curvature of the glenoid: (0) Saddle-shaped, oval and elongate; (1) Uniformly concave and more rounded in outline.
261- Medial surface of the scapula: (0) Convex; (1) Flat.
262- Suprascapular incisure (defined as the prominent emargination on the cranial border of the supraspinus fossa): (0) Absent; (1) Present.

Forelimb and Manus

263- Humeral head: (0) Subspherical, weakly inflected; (1) Spherical, strongly inflected.
264- Intertubercular groove of the humerus: (0) Shallow and broad; (1) Narrow and deep.
265- Size of the lesser tubercle of the humerus relative to the greater tubercle: (0) Wider; (1) Narrower.
266- Torsion between the proximal and distal ends of the humerus: (0) Strong (≥30 degrees); (1) Moderate (30–15 degrees); (2) Weak.

267- Ventral extension of the deltopectoral crest or the position of the deltoid tuberosity: (0) Short and limited to the proximal part of the humeral shaft; (1) Extending ventrally (distally) at least 1/3 the length of the shaft.
268- Teres tuberosity on medial side of humerus. (0) Absent; (1) Present; (2) Hypertrophied.
269- Ulnar articulation on the distal humerus: (0) Bulbous ulnar condyle; (1) Cylindrical trochlea in posterior view with a vestigial ulnar condyle in anterior view; (2) Cylindrical trochlea without an ulnar condyle (cylindrical trochlea extending to the anterior/ventral side).
270- Radial articulation on the distal humerus: (0) Distinct and rounded radial condyle in both anterior (ventral) and posterior (dorsal) aspects (that does not form a continuous synovial surface with the ulnar articulation in the ventral/anterior view of the humerus); (1) Rounded radial condyle anteriorly but cylindrical posteriorly; (2) Capitulum (forming a continuous synovial surface with the ulnar trochlea; cylindrical in both anterior and posterior aspects).
271- Entepicondyle and ectepicondyle of the humerus: (0) Robust; (1) Weak.
272- Sigmoidal shelf for the supinator ridge extending proximally from the ectepicondyle:
273- Coronoid process of semilunar notch of ulna: (0) Absent; (1) Present and level to olecranon process; (2) Present and higher than olecranon process.
274- Styloid process of the radius: (0) Weak; (1) Strong.
275- Enlargement of the scaphoid: (0) Not enlarged (scaphoid ≤150% of the lunate); (1) Enlarged (scaphoid twice the size of the lunate); (2) Enlarged with a distolateral process.
276- Size and shape of the hamate (unciform): (0) About equal size to the triquetrum, anteroposteriorly compressed; (1) Hypertrophied, much larger than the triquetrum, mediolaterally compressed.
277- Trapezium morphology and proportion: (0) Elongate to cuboidal, larger than or subequal to the trapezoid; (1) Bean-shaped or fusiform, smaller than the trapezoid.
278- Triquetrum-lunate proportion: (0) Triquetrum nearly twice the size of the lunate; (1) Triquetrum subequal to the lunate.
279- Relative length of metacarpals (MC) to proximal phalanx (PP) of digit III: (0) PP shorter than MC; (1) PP longer than MC.

Pelvic Girdle

280- Anterior process of the ilium: (0) Short (less than the diameter of the acetabulum); (1) Long, 1-1.5 times the diameter of the acetabulum; (2) Elongate, more than 1.5 times the diameter of the acetabulum.
281- Posterior process of the ilium: (0) Present; (1) Reduced or absent.
282- Acetabular dorsal emargination: (0) Open (emarginated); (1) Closed (with a complete rim).

Patagomaia: 1, closed (with a complete rim).

283- Sutures of the ilium, ischium, and pubis within the acetabulum: (0) Present; (1) Fused.

Patagomaia: 1, fused.

284- Ischiatic dorsal margin and tuberosity: (0) Dorsal margin concave (emarginated) and ischiatic tuberosity present; (1) Dorsal margin concave and ischiatic tuberosity hypertrophied; (2) Dorsal margin straight and ischiatic tuberosity small.

Patagomaia: 2, Dorsal margin straight and ischiatic tuberosity small.

285- Posterior spine of the ischium: (0) Short and pointed; (1) Expanded with oblique posterior spine; (2) Expanded and truncated.
286- Epipubic bone: (0) Present; (1) Absent.
287- Width of epipubis: (0) Narrow; (1) Wide.
288- Fusion of the sacral vertebrae with the proximal caudal vertebrae: (0) Absent; (1) Present.

289- Fusion of the ischium with the caudal vertebrae: (0) Absent; (1) Present.
290- Preacetabular tubercle on the ilium for M. rectus femoris: (0) Absent; (1) Present.
Patagomaia: 0, absent.
291- Fully encircled synovial surface inside the acetabulum: (0) Absent; (1) Present.
Patagomaia: 0, absent.
292- Lesser psoas tuberosity or process on the pubis: (0) Absent; (1) Present.
Patagomaia: 1, present.

Hindlimb and Pes

293- Inflected head of the femur set off from the shaft by a neck: (0) Neck absent and head oriented dorsally; (1) Neck present, head spherical and inflected medially.
Patagomaia: 1, Neck present, head spherical and inflected medially.
294- Fovea for the acetabular ligament on the femoral head: (0) Absent; (1) Present.
Patagomaia: 1, present.
295- Orientation of the greater trochanter: (0) Directed dorsolaterally; (1) Directed dorsally.
Patagomaia: 1, directed dorsally.
296- Level of greater trochanter relative to femoral head: (0) Mid-level of femoral head; (1) Top level of femoral head.
Patagomaia: 0, mid-level of femoral head.
297- Position of the lesser trochanter: (0) On medial side of the shaft; (1) On the ventromedial or ventral side of the shaft.
Patagomaia: 1, On the ventromedial or ventral side of the shaft.
298- Size of the lesser trochanter: (0) Large; (1) Small to absent.
Patagomaia: 1, small.
299- The third trochanter of femur: (0) Absent; (1) Present; (2) Present as a continuous ridge connected to the greater trochanter.
300- Patellar facet (‘groove’) of the femur: (0) Absent; (1) Shallow and weakly developed; (2) Well-developed.
Patagomaia: 1, shallow and weakly developed.
301- Proximo-lateral tubercle or tuberosity of the tibia: (0) Large and hook-like; (1) Indistinct; (2) Fused to fibula.
Patagomaia: 1, indistinct.
302- Distal tibial malleolus: (0) Weak; (1) Distinctive.
303- Differentiation of lateral tibio-astragalar condyle from the medial tibio-astragalar condyle: (0) Absent; (1) Present.
304- Fibula contacting the distal end of the femur: (0) Present; (1) Absent; (2) Fibula contacting through fusion with the tibia.
305- Fused distal portions of the tibia and fibula: (0) Absent; (1) Present.
306- Enlarged parafibular structure of the fibula: (0) Absent; (1) Present; (3) Present and hypertrophied.
307- Parafibula types: (0) Separate bone and unfused to the fibular; (1) Fused to fibula as an enlarged process.
308- Distal fibular styloid process: (0) Weak or absent; (1) Distinct; (2) Elongate to form a full contact with lateral surface of astraglar trochlea.
309- Fibula contacting the calcaneus (= ‘tricontact in upper ankle joint’): (0) Extensive contact; (1) Reduced; (2) Absent.
310- Superposition (overlap) of the astragalus over the calcaneus (lower ankle joint): (0) Little or absent; (1) Weakly developed; (2) Present.
311- Astragalo-navicular articulation – symmetry to the neck: (0) Articulating facet indistinctive; (1) Asymmetrical: present only on the lateral side of the “neck region”; (2) Symmetrical with regard to the astragalar neck.
312- Astragalar neck basal width (justification for separating this character from the navicular facet expansion is that the latter concerns symmetry, whereas this character deals with proportion; the distributions of these two character are

different in some basal eutherians and crown marsupials): (0) Neck narrower than the head (constriction posterior to navicular facet); (1) Neck about same width as the head (with parallel sides posterior to navicular facet); (2) Widest point of neck at mid-length (widening is not developed near the base of the neck); (3) Astragalar neck widest at the base.

313- Astragalonavicular contact aspect ratio: (0) Navicular contact transversely wider than dorsoventrally thick; (1) Navicular contact dorsoventrally thicker than transversely wide.

314- Expansion and dorso-ventral orientation of navicular contact in the astragalar head region: (0) Restricted anteriorly (navicular contact narrower than the base of the head); (1) Asymmetrical spread only to the medial side of the astragalar "head-neck region"; (2) Navicular facet and sides of the neck form a rectangular outline; (3) Symmetrical spread of the navicular facet to both the lateral and the medial sides of the neck (symmetrical with regards to the main axis of the neck); (4) Navicular facet spread underneath the head-neck region so that part of navicular facet faces ventrally, and astragalar head superpositioned on part of the navicular bone.

315- Astragalo-navicular contact shape: (0) Flat to convex; (1) Crest-in-groove: Transverse groove on astralar head to receive crest from navicular.

316- Astragalar trochlea (defined as a saddle-shaped upper ankle joint): (0) Absent; (1) Present, but weak (defining crest on the medial astragalo-tibial facet weakly developed); (2) Present, with clear separation of the medial and lateral tibial facets.

317- Well-defined medio-tibial crest (more or less parallel to the tibio-fibular crest) on the astragalus: (0) Absent; (1) Present.

318- Astragalar medial plantar tuberosity: (0) Absent; (1) Present, but weakly developed; (2) Present, and ventrally flaring or protruding.

319- Distal end of the calcaneal tubercle: (0) Short, dorso-ventrally compressed, without a terminal swelling; (1) doro-ventrally compressed, with a terminal swelling; (2) Elongate, vertically deep, and mediolaterally compressed, with terminal swelling.

320- Ventral orientation of terminal swelling of calcaneal tuber: (0) Absent; (1) Present.

321- Morphology of the peroneal process of the calcaneus: (0) Laterally expanded shelf, larger than the combined length of the sustentacular and astragalar facets, lateral to the astragalar facet; (1) With a distinct and long peroneal process, laterally projecting; (2) With a distinct peroneal process, demarcated by a deep peroneal groove at the base; (3) Laterally directed, small peroneal shelf demarcated from the anterior (cuboidal) edge of the calcaneus; (4) Anterolaterally directed, hypertrophied peroneal process/shelf; (5) Peroneal structure laterally reduced (lateral surface is straight from the calcaneal tubercle).

322- Placement of the base of the peroneal process relative to the level of the cuboid facet of the calcaneus: (0) Peroneal structure posterior to the level of the cuboid facet; (1) Peroneal structure developed anteriorly at the same level as the cuboid facet; (2) Peroneal structure hypertrophied, extending anteriorly beyond the level of the cuboid facet.

323- Peroneal groove of the calcaneus: (0) Indistinct, on the anterolateral aspect of the lateral shelf; (1) Distinct, located in deep separation of the peroneal process from the calcaneal body; (2) Developed, either on the lateral side of the process or on the anterolateral extremity of the peroneal process.

324- Alignment of the cuboid to the main axis of the calcaneus (horizontal plane): (0) On the anterior (distal) end of the calcaneus (the cuboid is aligned with the long axis of the calcaneus); (1) On the anteromedial aspect of the calcaneus (the cuboid is skewed to the medial side of the long axis of the calcaneus):

325- Orientation of the calcaneocuboid joint in dorso-ventral plane: (0) Calcaneocuboid facet on the calcaneus oriented ventrally (more visible in plantar view than in dorsal view); (1) Calcaneocuboid facet oriented anteriorly (distally); (2) Calcaneocuboid facet oriented ventromedially or medio-obliquely.

326- Saddle-shaped calcaneocuboid joint: (0) Calcaneocuboid facet on the calcaneus relatively flat to slightly concave; (1) Saddle-shaped (differentiation of dorsal vs. proximal calcaneocuboid "facets" so that the whole calcaneocuboidal joint is saddle-shaped).
327- Lower ankle joint - orientation of the sustentacular facet of the calcaneus in relation to the horizontal plane: (0) Nearly vertical; (1) Oblique (≤70 degrees) to nearly horizontal.
328- Antero-posterior placement of the sustentacular facet relative to the astragalar facet on the calcaneus: (0) Directly anterior to the astragalar facet and vertically oriented on the medial edge of the calcaneus; (1) On the dorsal aspect and positioned anteromedial to the astragalar facet on the calcaneus; (2) On the dorsal aspect, medial to the astragalar facet; (3) On the dorsal aspect, anterior to the astragalar facet.
329- Confluence of the sustentacular facet and the astragalar facet on the calcaneus: (0) Absent; (1) Present.
330- Ventral outline of the sustentacular process of the calcaneus: (0) Indistinctive; (1) Medially directed shelf, with rounded outline; (2) Protruding triangle, posteromedially directed;
331- Antero-posterior position of the sustentacular facet/process (using the most salient point of the facet/process in ventral view as landmark) relative to the length of the calcaneus: (0) Near the mid-point; (1) Near the anterior (proximal) one-third.
332- Shape of posterior calcaneo-astragalar process/protuberance and its contiguous fibular contact (if the fibula contact is present in medial view) on the calcaneus: (0) Indistinctive (boundary not defined and confluent with fibular contact); (1) Well defined, and oblong to ellipsoidal; (2) Nearly spherical and bulbous, more transversely developed than character state 1; (3) Transversely confluent with the sustentacular facet.
333- Placement of the Calcaneo-Astraglar Facet (CAF) structure (structure of the calcaneoastragalar contact): (0) On the medial side of the body of the calcaneus; (1) On the dorsal side of the body of the calcaneus, but bordering on the body's medial margin (without a protruding outline); (2) On the dorsal side of the body of the calcaneus and protruding beyond the body's medial margin; (3) Withdrawn and separated from the medial margin and placed along the lateral margin of the body of the calcaneus.
334- Orientation of Calcaneo-Astraglar Facet (CAF) relative to Calcaneo-Fibulo-Facet (CFF): (0) CAF anterior to CFF; (1) CAF medial to CFF.
335- Anterior ventral (plantar) tubercle of the calcaneus: (0) Absent; (1) Present, at the anterior edge (just lateral to the cuboid facet); (2) Present, set back from the anterior edge.
336- Anteroventral groove or depression of the calcaneus: (0) Absent; (1) Present.
337- Shape of the body of the calcaneus at the level of the posterior calcaneoastragalar facet: (0) Dorso-ventrally compressed; (1) Mediolaterally compressed.
338- Ventral curvature of the calcaneal tubercle: (0) Present; (1) Absent.
339- Proportion of the navicular and cuboid (transverse width measured in dorsal view): (0) Navicular narrower than or subequal to cuboid; (1) Navicular wider than cuboid.
340- Proportion of the entocuneiform, mesocuneiform, and ectocuneiform (in ventral view): (0) Mesocuneiform and ectocuneiform small, their combined width smaller than the width of the entocuneiform; (1) Mesocuneiform and ectocuneiform large, their combined width (in dorsal view) exceeding the width of the entocuneiform.
341- Saddle-shaped contact between entocuneiform and proximal end of metatarsal 1: (0) Absent; (1) Present.
342- Medio-plantar aspect of the cuboid deeply notched by the peroneus longus tendon: (0) Absent; (1) Present.
343- Prehallux: (0) Absent; (1) Present.

344- End-to-end contact of metatarsal V and the peroneal process of the calcaneus: (0) Absent; (1) Present.
345- Relationships of the proximal end of metatarsal V to the cuboid: (0) Metatarsal V is off-set from the lateral side of the cuboid; (1) Metatarsal V is so far off-set to the side of the cuboid that it contacts the calcaneus; (2) Metatarsal V is level with (not off-set from) the anterior end of the cuboid.
346- Ventrolateral tubercle at the proximal end of metatarsal V: (0) Absent or indistinctive; (1) Present, at or anterior to the anterior edge of the calcaneus; (2) Present, off-set posteriorly from the anterior edge of the calcaneus.
347- Angle of metatarsal III to the calcaneus (which indicates how much the sole of the foot is 'bent' from the long axis of the ankle): (0) Metatarsal III aligned with (or parallel to) the long axis of the calcaneus; (1) Metatarsal III arranged obliquely from the long axis of the calcaneus.
348- Metatarsal II and metatarsal III proximal ends: (0) II and III even or II more proximal than III; (1) III more proximal than II.
349- Opposable hallux: (0) Absent; (1) Present.
350- Relative length of metatarsals and proximal phalanx of digit III: (0) PP shorter than MT; (1) PP longer than MT.

Other Postcranial Characters

351- Ossified patella: (0) Absent; (1) Present.
352- Sesamoid bones in the digital flexor tendons: (0) Absent; (1) Present, unpaired; (2) Present, paired.
353- External pedal (tarsal) spur: (0) Absent; (1) Present.
354- Pes digital grouping: (0) Didactylous; (1) Syndactylous.
355- Epiphyses in long bones of zeugopodials: (0) Absent; (1) Present.

Basicranium

356- External size of the cranial moiety of the squamosal: (0) Narrow; (1) Broad; (2) Expanded posteriorly to form the skull roof table.
357- Participation of the cranial moiety of the squamosal in the endocranial wall of the braincase: (0) Absent; (1) Present.
358- Multiple vascular foramina (for rami temporales) in the squamosal and parietal: (0) Absent; (1) Present.
359- Multiple vascular foramina (for branches of external ethmoidal artery) in the dorsal surface of the frontal: (0) Absent; (1) Present.
360- Topographic relationships of the dentary-squamosal contact (or glenoid) and the cranial moiety of the squamosal (only applicable to taxa with the dentary-squamosal joint; this character is best seen in ventral view): (0) Contact on the internal aspect of the zygoma, without a constricted neck; (1) Contact on the zygoma, with a constricted neck; (2) Contact on the cranial moiety of squama; (3) On zygoma, without a constricted neck.
361- Cross-section profile of the squamosal anterior to its zygomatic root: (0) Rounded or triangular and tapering anteriorly; (1) Dorsoventrally expanded and mediolaterally compressed, and not tapering anteriorly.
362- Postglenoid depression on the squamosal: (0) Present as the post-craniomandibular joint sulcus ("external auditory meatus" on the zygoma); (1) Absent; (2) Present on the skull base.
363- Squamosal - entoglenoid process: (0) Absent or vestigial; (1) Present, but separated from the postglenoid process; (2) Present, enlarged and connected to the postglenoid process.
364- Position of the craniomandibular joint: (0) Posterior or lateral to the level of the fenestra vestibuli; (1) Anterior to the level of the fenestra vestibuli.
365- Orientation of the glenoid on the squamosal: (0) On the inner side of the zygoma and facing ventromedially; (1) On the platform of the zygoma and facing ventrally.

366- Postglenoid process of the squamosal: (0) Absent; (1) Postglenoid crest raised below the fossa, but without a distinctive process; (2) Distinctive process; (3) Distinctive process buttressed by ectotympanic.
367- Postglenoid foramen position: (0) Posterior to the glenoid area; (1) Medial to the postglenoid process; (2) Anterior to the postglenoid process.
368- Postglenoid foramen presence vs. absence and composition: (0) Absent; (1) Present, in the squamosal; (2) Present, between the squamosal and petrosal; (3) Present, between the squamosal and ectotympanic.
369- Medial margin of the glenoid fossa: (0) Formed by the squamosal; (1) Formed by the alisphenoid.
370- Squamosal - epitympanic recess (this character may be ordered): (0) No contribution to the “epitympanic area” of the petrosal; (1) Small contribution to the posterolateral wall of the epitympanic recess; (2) Large contribution to the lateral wall of the epitympanic recess; (3) Squamosal forming a large part of enlarged epitympanic sinus.
371- Contribution of the basisphenoid wing (parasphenoid ala) to the external bony housing of the cochlea: (0) Participates in the rim of the fenestra vestibuli; (1) Does not reach the rim of the fenestra vestibuli; (2) Absent or excluded from the cochlear housing.
372- Relationship of the cochlear housing to the lateral lappet of the basioccipital: (0) Entirely covered by the basioccipital; (1) Medial aspect covered by the basioccipital; (2) Partially (~about half width on the medial side) covered by the basioccipital; (3) Fully exposed as the promontorium.
373- Thickened rim of the fenestra vestibuli: (0) Present; (1) Absent.
374- Cochlear housing fully formed by the petrosal: (0) Absent; (1) Present.
375- Ventromedial surface of the promontorium: (0) Flat; (1) Inflated and convex.
376- Lateral wall and overall external outline of the promontorium: (0) Triangular, with a steep and slightly concave lateral wall; (1) Elongate and cylindrical; (2) Bulbous and oval shaped.
377- Cochlea: (0) Cochlear recess (without a canal); (1) Short canal; (2) Elongate canal, to the fullest extent of the promontorium; (3) Slightly curved; (4) Elongate and partly coiled; (5) Elongate and coiled to at least 360°.
378- Internal acoustic meatus - cribriform plate: (0) Absent; (1) Present.
379- Internal acoustic meatus depth: (0) Deep with thick prefacial commissure; (1) Shallow with thin prefacial commissure.
380- Primary bony lamina within the cochlear canal: (0) Absent; (1) Present.
381- Secondary bony lamina for the basilar membrane within the cochlear canal: (0) Absent; (1) Present.
382- Crista interfenestralis: (0) Horizontal, broad, and extending to the base of the paroccipital process; (1) Vertical, delimiting the back of the promontorium; (2) Horizontal, narrow, and connecting to the caudal tympanic process.
383- Post-promontorial tympanic recess: (0) Absent; (1) Present.
384- Rostral tympanic process of the petrosal promontorium: (0) Absent or low ridge; (1) present as a ridge of the promontorium.
385- Caudal tympanic process of the petrosal: (0) Absent; (1) Present; (2) Present, notched; (3) Present, hypertrophied and buttressed against the exoccipital paracondylar process.
386- Petrosal - tympanic process: (0) Absent; (1) Present.
387- Rear margin of the auditory region: (0) Marked by a steep wall; (1) Extended onto a flat surface.
388- Prootic canal: (0) Absent; (1) Present, vertical; (2) Present, horizontal and reduced.
389- Position of the sulcus for the anterior distributary of the transverse sinus relative to the subarcuate fossa: (0) Anterolateral; (1) Posterolateral.

390- Lateral trough floor anterior to the tympanic aperture of the prootic canal and/or the primary facial foramen: (0) Open lateral trough, no bony floor; (1) Bony floor present; (2) Lateral trough absent.
391- Anteroventral opening of the cavum epiptericum: (0) Present; (1) Present, with reduced size (due to the anterior expansion of the lateral trough floor); (2) Present, partially enclosed by the petrosal; (3) Present, enclosed by the alisphenoid and petrosal; (4) Present, as large piriform fenestra.
392- Enclosure of the geniculate ganglion by the bony floor of the petrosal in the cavum supracochleare: (0) Absent; (1) Present.
393- Hiatus Fallopii: (0) Present, in the petrosal roof of the middle ear; (1) Present, at the anterior end of the petrosal; (2) Absent (applicable only to those taxa with a cavum supracochleare).
394- Foramen ovale - composition: (0) Between the petrosal and alisphenoid; (1) Secondary foramen partially or fully enclosed by the alisphenoid, in addition to the primary foramen between the petrosal and alisphenoid; (2) In the petrosal (anterior lamina); (3) Between the alisphenoid and squamosal; (4) Within the alisphenoid.
395- Foramen ovale - position: (0) On the lateral wall of the braincase; (1) On the ventral surface of the skull.
396- Number of exit(s) for the mandibular branch of the trigeminal nerve (V3): (0) One; (1) Two.
397- Quadrate ramus of the alisphenoid: (0) Forming a rod underlying the anterior part of the lateral flange; (1) Absent.
398- Alisphenoid canal (for the ramus inferior and/or ramus infraorbitalis): (0) Absent;(1) Present.
399- Anterior lamina exposure on the lateral braincase wall: (0) Present; (1) Reduced or absent.
400- Orientation of the anterior part of the lateral flange: (0) Horizontal shelf; (1) Ventrally directed; (2) Medially directed and contacting the promontorium; (3) Vestigial or absent.
401- Vertical component of the lateral flange (‘L-shaped’ and forming a vertical wall to the pterygoparoccipital foramen): (0) Present; (1) Absent.
402- Vascular foramen in the posterior part of the lateral flange (and anterior to the pterygoparoccipital foramen): (0) Present; (1) Absent.
403- Relationship of the lateral flange to the crista parotica (or the anterior paroccipital process that bears the crista): (0) Widely separated; (1) Narrowly separated; (2) Continuous.
404- Pterygoparoccipital foramen (for the ramus superior of the stapedial artery): (0) Laterally open notch; (1) Foramen enclosed by the petrosal or squamosal; (2) Absent.
405- Position of the pterygoparoccipital foramen relative to the level of the fenestra vestibuli: (0) Posterior or lateral; (1) Anterior.
406- “Bifurcation of the paroccipital process” - presence vs. absence (this is modified from the character used in several previous studies): (0) Absent; (1) Present.
407- Posterior paroccipital process of the petrosal: (0) No ventral projection below the level of the surrounding structures; (1) Projecting below the surrounding structures.
408- Morphological differentiation of the anterior paroccipital region: (0) Anterior paroccipital is bulbous and distinctive from the surrounding structures; (1) Anterior paroccipital region has a distinct crista parotica.
409- Epitympanic recess: (0) Absent; (1) Present.
410- Epitympanic recess topographic relationship: (0) Lateral to crista parotica; (1) Posterior to crista parotica.
411- Tympanohyal contact with the cochlear housing: (0) Absent; (1) Present.
412- Relationship of the squamosal to the paroccipital process: (0) Squamosal covers the entire paroccipital region; (1) No squamosal cover on the anterior paroccipital region; (2) Squamosal covers a part of the paroccipital region, but not the crista

parotica (the squamosal wall and the crista parotica are separated by the epitympanic recess).
413- Medial process of the squamosal reaching toward the tympanic cavity: (0) Absent;(1) Present (near or bordering on the foramen ovale).
414- Stapedial artery sulcus on the petrosal: (0) Absent; (1) Present.
415- Transpromontorial sulcus for the internal carotid artery on the cochlear housing: (0) Absent; (1) Present.
416- Deep groove on the anterior pole of the promontorium: (0) Absent; (1) Present.
417- Perbullar canal or sulcus for the internal carotid artery: (0) Absent; (1) Present.
418- Epitympanic wing medial to the promontorium: (0) Absent; (1) Present.
419- Basioccipital pharyngeal crest: (0) Absent (1) Present.
420- Paired basioccipital foramina (0) Absent; (1) Present.
421- Ectopterygoid process of the alisphenoid: (0) Absent; (1) Present.
422- Tympanic process of the alisphenoid: (0) Absent; (1) Present, but limited to the “piriform” region of the basicranium; (2) Intermediate; (3) Well-developed, extending to near the jugular foramen.
423- Hypotympanic recess in the junction of the alisphenoid, squamosal, and petrosal: (0) Absent; (1) Present.
424- Separation of the fenestra cochleae from the jugular foramen: (0) Absent; (1) Separate but within the same depression; (2) Separate (not within the same depression).
425- Channel of the perilymphatic duct: (0) Open channel and sulcus; (1) At least partially enclosed channel.
426- Jugular foramen size relative to the fenestra cochleae (applicable only to those taxa with a jugular foramen fully separated from the fenestra cochleae): (0) Jugular subequal to the fenestra cochleae; (1) Jugular larger than the fenestra cochleae.
427- Relationship of the jugular foramen to the opening of the inferior petrosal sinus: (0) Confluent; (1) Separate.
428- Stapedial muscle fossa size: (0) Absent; (1) Present, small; (2) Present, large (twice the size of the fenestra vestibuli).
429- Alignment of the stapedial fossa relative to the crista interfenestralis: (0) Aligned with crista interfenestralis; (1) Lateral to the crista interfenestralis.
430- Hypoglossal foramen: (0) Indistinct, either confluent with the jugular foramen or sharing a depression with the jugular foramen; (1) Separated from the jugular foramen; (2) Separated from the jugular foramen; the latter with a circular, raised external rim.
431- Number of separate hypoglossal foramina: (0) Single; (1) Double.

Middle Ear Ossicle Characters

432- Geometry (shape) of the incudo-mallear contact: (0) Trochlear (convex and cylindrical) surface of the incus; (1) Trough; (2) Saddle-shaped contact on the incus; (3) Flat surface.
433- Alignment of the incus and the malleus: (0) Posterior-anterior; (1) Posteromedial to anterolateral; (2) Dorsoventral.
434- Twisting of the dorsal plate relative to the trochlea on the quadrate: (0) Dorsal plate aligned with the trochlea; (1) Dorsal plate twisted relative to the trochlea, (2) Dorsal plate twisted and elevated from the trochlea; (3) Dorsal plate reduced to a conical process (crus longum).
435- Presence of a quadrate/incus neck (slightly constricted region separating the dorsal plate or crus breve from the trochlea; this represents the differentiation between the ‘body’ and crus breve of the incus): (0) Absent; (1) Present.
436- Dorsal plate (= crus brevis) of the quadrate/incus: (0) Broad plate; (1) Pointed triangle; (2) Reduced.
437- Incus - angle of the crus brevis to crus longum of the incus (this is equivalent to the angle between the dorsal plate and the stapedial process of the quadrate): (0)

Alignment of the stapedial process (crus longum) and the dorsal plate (crus breve) (or an obtuse angle between the two structure) (distinctive process is lacking, stapes/incus contact is on the medial side of the quadrate trochlea); (1) Perpendicular or acute angle of the crus breve and crus longum (“A- shaped” incus).

438- Primary suspension of the incus/quadrate on the basicranium: (0) By quadratojugal in addition to at least one other basicranial bone; (1) By squamosal only; (2) By petrosal (either by the preserved direct contact of the incus or by inference from the presence of a well-defined crista parotica).

439- Quadratojugal: (0) Present; (1) Absent.

440- Morphology of the stapes: (0) Columelliform–macroperforate; (1) Columelliform–imperforate (or microperforate); (2) Bicrurate–perforate.

441- Stapedial ratio: (0) Less than 1.4; (1) 1.4-1.8; (2) ≥1.8.

442- Bullate stapedial footplate: (0) Absent; (1) Present.

443- Malleolar neck: (0) Absent; (1) Present.

444- Length of the malleus manubrium: (0) Shorter than the combined width of the surangular and prearticular anterior to the incudo-malleolar joint; (1) longer than the combined width of surangular and prearticular.

445- Thickness of malleolar manubrium: (0) robust; (1) gracile.

446- Distinctive angle or bending of Meckel’s bone (=anterior portion of ossified postdentary rod) anterior to the level of ectotympanic (angular) bone: (0) Absent; (1) Present.

447- Medio-lateral contact vs. separation of Meckel’s element (either independent or as an ossified component of the “postdentary rod”) from the posterior (pterygoid) region of mandible: (0) Presence of medio-lateral contact either in adult or in embryonic stage until Meckel’s cartilage re-absorption; (1) Embryonic Meckel’s cartilage medio-laterally separated from the posterior part of mandible; (2) Ossified Meckel’s cartilage medio-laterally separated from the posterior part of mandible:

448- Ectotympanic size/shape (may be ordered): (0) Plate-like; (1) Curved and rod-like; (2) Ring-shaped; (3) Slightly expanded (fusiform); (4) Expanded; (5) Tube-like.

449- Ectotympanic arc: (0) ≤ 70 degrees: (1) 90 – 135 degrees; (2) ≥ 135 degrees.

450- Anterior process of the ectotympanic (angular): (0) Present; (1) Absent.

451- Position/orientation of the incisura tympanica: (0) Posteroventral; (1) Posterior; (2) Postero-dorsal; (3) Dorsal.

452- Fusion of the ectotympanic to other cranial bones: (0) Absent; (1) Fused to other bones.

453- Entotympanic and its contribution to the bullar structure: (0) Absent; (1) Present.

Other Cranial Characters

454- Posterior extent of the bony secondary palate: (0) Anterior to the posterior end of the tooth row; (1) Level with the posterior end of the tooth row; (2) Extending posterior to the tooth row; (3) Extending to the basisphenoid-basioccipital suture.

455- Posterior median spine (or torus) on the palate: (0) Absent; (1) Present.

456- Pterygopalatine ridges: (0) Present; (1) Absent.

457- Transverse process of the pterygoid: (0) Present and massive; (1) Present but reduced (as the hamulus); (2) Greatly reduced (with a vestigial crest on pterygoid) or absent.

458- Pterygoids contact on midline of pharyngeal roof: (0) Present; (1) Absent.

459- Ventral opening of the minor palatine foramen: (0) Encircled by the pterygoid (and ectopterygoid if present) in addition to the palatine; (1) Encircled by the palatine and maxilla, separated widely from the subtemporal margin; (2) Encircled completely by the palatine (or between palatine and maxilla), large, with thin bony bridge from the subtemporal margin; (3) Large, posterior fenestration; (4) Notch.

460- Transverse canal foramen: (0) Absent; (1) Present.

461- Carotid foramen position: (0) Within the basisphenoid; (1) Within the basisphenoid/basioccipital suture; (2) Within the basisphenoid/petrosal suture; (3) Through the opening of the cavum epiptericum.
462- Overhanging roof of the orbit: (0) Absent; (1) Present, formed by the frontal.
463- Exit(s) of the infraorbital canal: (0) Numerous small foramina of similar size; (1) At least a single large, with smaller, anteroventral accessory foramina; (2) One large foramen.
464- Composition of the posterior opening of the infraorbital canal (maxillary foramen): (0) Between the lacrimal, palatine, and maxilla; (1) Exclusively enclosed by the maxilla; (2) Enclosed by the maxilla, frontal and palatine.
465- Size and shape of the lacrimal: (0) Small, oblong-shaped on the facial part of the rostrum; (1) Large, triangle-shaped on the facial portion of rostrum; (2) Crescent shaped on the facial portion of the rostrum; (3) Reduced to an anteroposteriorly narrow strap confined to the antorbital margin; (4) Absent from the facial portion of the rostrum.
466- Location of the lacrimal foramen: (0) Within the orbit; (1) On the facial side of the lacrimal (anterior to or on the anterior orbital margin).
467- Number of lacrimal foramina: (0) One; (1) Two.
468- Lacrimal foramen composition: (0) Within the lacrimal; (1) Bordered by or within the maxilla.
469- Maximum vertical depth of the zygomatic arch relative to the length of the skull (this character is designed to indicate the robust vs. gracile nature of the zygomatic arch): (0) Between 10-20%; (1) Between 5-7%; (2) Zygoma incomplete.
470- Ultimate upper molar implanted in the anterior root of zygoma: (0) Absent. (1) Present.
471- Maxillary zygomatic tuberosity. (0) Absent; (1) Present.
472- Frontal/alisphenoid contact: (0) Dorsal plate of the alisphenoid contacting the frontal at the anterior corner; (1) Dorsal plate of the alisphenoid with more extensive contact with the frontal (~50% of its dorsal border); (2) Absent.
473- Frontal-maxilla facial contact: (0) Absent; (1) Present.
474- Nasal-frontal suture - medial process of the frontals wedged between the two nasals: (0) Absent; (1) Present.
475- Posterior width of the nasal bones: (0) Broader than the width at the mid-length of the nasal; (1) Reduced/narrow; (2) Parallel-sided with straight sutures (consistent width throughout length).
476- Pila antotica: (0) Present; (1) Absent.
477- Fully ossified medial orbital wall of the orbitosphenoid: (0) Absent; (1) Present, forming the ventral floor of the braincase but not the entire orbital wall; (2) Present, forming both the braincase floor and the medial orbital wall.
478- Separation of the optic foramen from the sphenorbital fissure: (0) Absent; (1) Present.
479- Exit for maxillary nerve: (0) Separate from sphenorbital fissure, behind alisphenoid; (1) Separate from sphenorbital fissure, within alisphenoid; (2) Confluent with sphenorbital fissure.
480- Separate anterior opening of orbitotemporal canal: (0) Absent; (1) Present.
481- Orbital opening for the minor palatine nerve: (0) Absent; (1) Present.
482- Anterior part of the jugal on the zygoma: (0) Anterior part of the jugal extends to the facial part of the maxilla and forms a part of the anterior orbit; (1) Anterior part of the jugal does not reach the facial part of the maxilla and is excluded from the anterior orbit margin.
483- Jugal lateral exposure on zygoma: (0) Long, extending to at least 2/3 of the zygoma; (1) Short, limited to anterior 1⁄2 of the zygoma; (2) Not exposed on lateral aspect of zygoma.

484- Posterior part of the jugal: (0) Contributes to the squamosal glenoid; (1) Borders on but does not contribute to the squamosal glenoid; (2) Terminates anterior to the squamosal glenoid.
485- Maxillary in the sub-temporal margin of the orbit: (0) Absent; (1) Present; (2) Present and extensive; (3) Present and extremely extended to hamulus.
486- Orbital process of the frontal borders on the maxilla within orbit: (0) Absent; (1) Present.
487- Anterior ascending vascular channel (for the arteria diploëtica magna) in the temporal region: (0) Open groove; (1) Partially enclosed in a canal; (2) Completely enclosed in a canal or endocranial; (3) Absent.
488- Posttemporal canal for the arteria and vena diploëtica: (0) Present, large; (1) Small; (2) Absent.
489- Nuchal crest: (0) Overhanging the concave or straight supraoccipital; (1) Weakly developed with convex supraoccipital.
490- Sagittal crest: (0) Prominently developed; (1) Weakly developed; (2) Absent.
491- Tabular bone: (0) Present; (1) Absent.
492- Occipital slope: (0) Occiput sloping posterodorsally (or vertically oriented) from the occipital condyle; (1) Occiput sloping anterodorsally from the occipital condyle (such that the lambdoidal crest is leveled anterior to the occipital condyle and condyle is fully visible in dorsal view of the skull).
493- Occipital artery groove on the occiput extending dorsal to the posttemporal foramen: (0) Absent; (1) Present.
494- Foramina on the dorsal surface of the nasals: (0) Absent; (1) Present.
495- Septomaxilla: (0) Present, with the ventromedial shelf; (1) Present, without the ventromedial shelf; (2) Absent.
496- Internarial/dorsal process of the premaxilla: (0) Present on nasal midline suture;(1) Absent/extremely reduced.
497- Posterodorsal process of the premaxilla length: (0) Short (i.e, does not extend beyond level of anterior maxillary tooth); (1) intermediate (i.e., extends beyond level of anterior maxillary tooth); (2) long (i.e., contacts frontal posteriorly).
498- Facial part of the premaxilla borders on the nasal: (0) Absent; (1) Present.
499- Premaxilla - palatal process relative to the canine alveolus: (0) Does not reach to the level of the canine alveolus; (1) Reaches the level of the canine alveolus.
500- Incisive foramina size: (0) Small (one or two incisors); (1) Intermediate (three or four incisors); (2) Large (more than half the palatal length).
501- Palatal vacuities: (0) Absent; (1) Present, near palatomaxillary border; (2) Present, either positioned near or extended to the posterior edge of bony palate.
502- Major palatine foramina: (0) Present. (1) Absent.
503- Ossified ethmoidal cribriform plate of the nasal cavity: (0) Absent; (1) Present.
504- Posterior excavation of the nasal cavity into the bony sphenoid complex: (0) Absent; (1) Present; (2) Present and partitioned from the nasal cavity.

Cranial Vault and Brain Endocast Characters

505- External bulging of the braincase in the parietal region: (0) Absent; (1) Expanded (the parietal part of the cranial vault is wider than the frontal part, but the expansion does not extend to the lambdoidal region); (2) Greatly expanded (expansion of the cranial vault extends to the lambdoidal region).
506- Anterior expansion of the vermis (central lobe of the cerebellum): (0) Absent; (1) Present.
507- Overall size of the vermis: (0) Small; (1) Enlarged.
508- Lateral cerebellar hemisphere (excluding the paraflocculus): (0) Absent; (1) Present.
509- External division on the endocast between the olfactory lobe and the cerebral hemisphere (well-defined transverse sulcus separating the olfactory lobes from the

cerebrum): (0) Absence of external separation of the olfactory lobe from cerebral hemisphere; (1) Enlarged olfactory lobes; (2) Clear division of transverse sulcus.
510- Encephalization quotient: (0) Below 0.13; (1) Between 0.15-0.25, (2) Above 0.26.
511- Expansion of the posterior cerebral hemisphere (for each hemisphere, not the combined width of the posterior hemispheres): (0) Absent; (1) Present.

Soft-tissue characters
512- Trophoblasts in the placenta: (0) Absent; (1) Present.
513- Müllerian ducts (oviduct and uterus) pass in between the ureters: (0) Absent; (1) Present.
514- Placental types: (0) Placenta absent; (1) Placenta present with vascularized chorio-allantois; (2) Placenta present but without vascularized chorio-allantois.

Features added for wear facet study by Luo et al.[119]
515- Multi-row and multicuspate molar opposition - Lower molar lingual row tallest anterior cusp a1 occluding into lingual embrasure between upper molars: (0) Absent;(1) Present.
516- Multi-row and multicuspate molar crown: saddle-shaped transverse crest between lingual cusp row (usually the tallest a1 on lowers, or A1 on uppers) and buccal cusp row (usually the tallest b2, or B2 on uppers): (0) Absent; (1) Present.

Added skull roof characters from Krause et al.[63]
517- Frontal anterior extent location: (0) Posterior to anterior border of orbit; (1) Anterior to orbit but posterior to anterior tip of lacrimal; (2) Anterior to lacrimal.
518- Morphology of frontal-parietal suture in dorsal view: (0) V-shaped, apex directed posteriorly; (1) U-shaped, convex posteriorly; (2) U-shaped, convex anteriorly; 3) Roughly transverse.
519- Contact between nasals and parietals: (0) Absent; (1) Present.

Added characters from Huttenlocket et al.[120]
520- Upper incisor alveolus depth: (0) Shallow (approximately 1.5x the alveolar diameter or less); (1) Deep (at least 2x the alveolar diameter or more): e.g., non-Megaconus eleutherodontids, Vintana.
521- Upper incisor alveolus orientation: (0) Vertical; (1) Procumbent: e.g., eleutherodontids, Vintana.
522- PMX facial process-nasal suture length (only applicable to taxa coded as '1' for character 497): (0) Less than 75% of maxilla-nasal suture length; (1) 75% to subequal to that of the maxilla.
523- Supraoccipital margin forms horizontal shelf over foramen magnum: (0) Absent; (1) Present.
524- Supraoccipital dorsal height: (0) Tall, incorporating the nuchal line; (1) Short, restricted from nuchal line.
525- Lambdoidal crest orientation in lateral view: (0) Vertical orientation; (1) Forms a posterior flare and overhang on lateral portion of lambdoidal crest; (2) Forms a posterior flare and overhang on median portion of lambdoidal crest (e.g., crown therians and basal therians).
526- Postparietal (if present): (0) Shallow and broad; (1) Forms large, subcircular shield, slightly taller than wide.
527- Parietal contribution to occiput: (0) Slight; (1) Enlarged, forming 30% of the occiput or more.
528- Maxilla-palatine suture orientation in ventral view: (0) Squared or arched; (1)Wedged anteromedially to midline (A-shaped) so that maxilla anterolaterally bounds palatine.
529- Posterior margin of bony secondary palate shape: (0) Level or truncated; (1) A-shaped notch.

530- Alisphenoid processus ascendens anteroposterior extent: (0) Anteroposteriorly broad; (1) Tall and narrow.
531- Maxilloturbinal supporting ridge: (0) Absent; (1) Present.
532- Ossified maxilloturbinal bones: (0) Absent; (1) Present.
533- Klinorhynchy: (0) Absent; (1) Present, anterior portion of skull is flexed anteroventrally.
534- Preoptic flexure on anteroventral portion of brain endocast: (0) Absent; (1) Present.
535- Mesiolateral divergence of left and right maxillary tooth rows: (0) Absent; (1) Present.
536- Pterygoid anterior expansion in ventral view: (0) Absent; (1) Present, pterygoid extends forward to contact maxilla in ventral view, excluding palatine medially.
537- Parieto-pachyostal sagittal crest: (0) Absent; (1) Present, parietal is greatly thickened on the sagittal midline.
538- Stepwise pattern ("en echelon" pattern of Jenkins et al. 1997) in the profile of upper premolar-molar series (applicable only to molars with more than one rows of multiple cusps): (0) Absent; (1) Present.
539- Dual-Mortar-Pestle occlusion of basined molars that are also partially multi-cusp-row (applicable only to taxa with multiple cusp-rows): (0) Absent; (1) Present.
540- Relative width of calcaneus as measured in length-width ratio. (0) Longer than wide, L/W ratio equal or greater than 150%; (1) Length sub-equal to width, L/W is 140%, or less.

Added characters from Zhou et al.[110]

541- Contact of medial ends of the two clavicles at the midline: (0) Present, point-contact or abutting contact; (1) No contact of clavicles at midline – two clavicles respectively contact the interclavicle/sternal manubrium. Note: All extant marsupials and placentals should be scored (1).
542- Urogenital sinus and vagina morphology: (0) Presence of cloaca (no differentiation of vagina from urogenital sinus, the latter confluent with rectum); (1) Differentiation of vagina from urogenital sinus: most extant placentals (except tenrecids, golden moles and soricids), and marsupials (except marsupial moles).
543- Vas deferens looping over ureter in adult males: (0) Absence; (1) Present: all extant placental species.
544- Descent of testis and location of testes in adult males: (0) No descent of testis (Testes abdominal, or testicondy); (1) Testis descended either ascrotal (testes in pelvic or inguinal position, but ascrotal and not in an external scrotum), or scrotal.
545- Development of scrotum: (0) Acrotal - testes in abdominal, pelvic, or inguinal position; (1) Scrotal – testes in scrotum.
546- Presence of ossified basihyal: (0) Absent; (1) Present.
547- Morphology of basihyal: (0) Rod-like basihyal; (1) Antero-posteriorly wide basihyal (strap-like or plate-like basihyal, which is widened in antero-posterior dimension, 25% or more of the transverse length of the bone).
548- Anterior cornu of hyoid apparatus: (0) Formed by a single, elongate hyoid rod without internal segmentation; (1) Jointed short segments of anterior cornua (scored for this character state where the cerato-basihyal joint is preserved, and scored also if the cerato-epihyal joint or the dorsal end of ceratohyal is preserved).
549- Thyrohyals: (0) Not ossified; (1) Ossified, rod-like along the shaft; (2) Ossified and strap-like in mid shalf; (3) Ossified, and oblong plate.
550- The expanded dorsal end of thyrohyal: (0) Absent; (1) Present and club-like or fan-like; (2) Present, broad and rhomboidal or semicircle-like.
551- Fusion of basihyal and thyrohyals: (0) Unfused (can be scored if the ends of basihyal are preserved); (1) Fused.
552- Fused basihyal and thyrohyals form an angled "V-bone": (0) Absent; (1) Present.
553- Morphology of ceratohyal: (0) Rod-like; (1) Strap-like; (2) Plate-like (broad, semicircle or rhomboidal shape); (3) Shortened and block-like.

554- Cartilaginous or ossified epihyal element in adult: (0) Absent; (1) Present
555- Ossified stylohyal in adult: (0) Absent; (1) Present.
556- Integro-cornuate versus discreto-cornuate condition of the jointed anterior cornu of hyoids: (0) Discreto-cornuate; (1) Integro-cornuate.

REFERENCES

1. Argot, C. Functional-adaptive analysis of the hindlimb anatomy of extant marsupials and the paleobiology of the Paleocene marsupials *Mayulestes ferox* and *Pucadelphys andinus*. *J Morphol* **253**, 76-108 (2002).

2. Luo, Z. X., & Ji, Q. New study on dental and skeletal features of the Cretaceous "symmetrodontan" mammal *Zhangheotherium*. *J Mammal Evol* **12**, 337-357 (2005).

3. Warburton, N. M., Yakovleff, M., & Malric, A. Anatomical adaptations of the hind limb musculature of tree-kangaroos for arboreal locomotion (Marsupialia: Macropodinae). *Australian J Zool* **60**, 246-258 (2012).

4. Chen, M., & Luo, Z. X. Postcranial skeleton of the Cretaceous mammal *Akidolestes cifellii* and its locomotor adaptations. *J Mammal Evol* **20**, 159-189 (2013).

5. O'Leary, M.A. et al. The placental mammal ancestor and the post–K-Pg radiation of placentals. *Science* **339**, 662–667 (2013).

6. Argot, C. Postcranial analysis of a carnivoran-like archaic ungulate: the case of *Arctocyon primaevus* (Arctocyonidae, Mammalia) from the late Paleocene of France. *J Mammal Evol*, **20**, 83-114 (2013).

7. Candela, A. M. & Picasso, M. B. Functional anatomy of the limbs of Erethizontidae (Rodentia, Caviomorpha): indicators of locomotor behavior in Miocene porcupines. *J Morphol* **269**, 552-593 (2008).

8. Taylor, M. E. The functional anatomy of the hindlimb of some African Viverridae (Carnivora). *J Morphol* **148**, 227-253 (1976).

9. Abello, M. A. & Candela, A. M. Paleobiology of *Argyrolagus* (Marsupialia, Argyrolagidae): an astonishing case of bipedalism among South American mammals. *J Mammal Evol* **27**, 419-444 (2020).

10. Fostowicz−Frelik, Ł. The hind limb skeleton and cursorial adaptations of the Plio−Pleistocene rabbit *Hypolagus beremendensis*. *Acta Palaeontol Pol* 52, 447–476 (2007).

11. Rose, K. D. Postcranial skeleton of Eocene Leptictidae (Mammalia), and its implications for behavior and relationships. *J Vert Paleontol* **19**, 355-372 (1999).

12. Argot, C. Functional adaptations of the postcranial skeleton of two Miocene borhyaenoids (Mammalia, Metatheria), *Borhyaena* and *Prothylacinus*, from South America. *Palaeontology* 46, 1213-1267 (2003).

13. Chinsamy, A. & Raath, M.A. Preparation of fossil bone for histological examination. *Pal Afr* **29**, 39–44 (1992).

14. Warshaw J. In *Mammalian evolutionary morphology: A tribute to frederick S. Szalay* (eds. Sargis, E. J. & Dagosto, M.) 385–425 (Dordrecht, Springer, 2008).

15. Horner, J.R., Ricqlès, A. & Padian, K. Variation in skeletochronological indicators of the hadrosaurid dinosaur *Hypacrosaurus*: implications for age assessment of dinosaurs. *Palaeobiology* **25**, 295–304 (1999).

16. Horner, J.R., Ricqlès, A. & Padian, K. Long bone histology of the hadrosaurid dinosaur *Maiasaura peeblesorum*: growth dynamics and physiology based on an ontogenetic series of skeletal elements. *J Vertebr Paleontol* **20**, 115–129 (2000).

17. Padian, K. & Horner, J.R. In *The Dinosauria. 2nd ed.* (eds. Weishampel, D.B., Dodson, P. & Osmolska, H.) 660–671 (Berkeley, University of California Press 2004).

18. Fostowicz-Frelik, L. & Sulej, T. Bone histology of *Silesaurus opolensis* Dzik, 2003 from the Late Triassic of Poland. *Lethaia* **43**, 137–148 (2010).

19. Knoll, F., Padian, K. & Riqclès, A. Ontogenetic change and adult body size of the early ornithischian dinosaur *Lesothosaurus diagnosticus*: implications for basal ornithischian taxonomy. *Gondwana Res* **17**, 171–179 (2010).

20. García Marsà, J.A., Agnolín, F.L. & Novas, F.E. Bone microstructure of *Lewisuchus admixtus* Romer, 1972 (Archosauria, Dinosauriformes). *Hist Biol* **31**, 157–162 (2017).

21. Ricqlès, A. Recherches paléohistologiques sur les os longs des tétrapodes. II. Quelques observations sur la structure des os logns des thériodontes. *Ann Paléont* **40**, 1–52 (1969).

22. Botha-Brink, J., Abdala, F. & Chinsamy-Turan, A. In *Forerunners of mammals: radiation, histology, biology* (Chinsamy-Turan, A., ed) 223–246 (Indiana, Indiana University Press, Bloomington, 2012).

23. García Marsà, J.A., Agnolín, F.L. & Novas, F.E. Comparative bone microstructure of two non-mammaliaform cynodonts from the Late Triassic (Carnian) Chañares formation of Northwestern Argentina. *Hist Bio* doi, 10.1080/08912963.2022.2149332 (2022)

24. Ray, S., Botha, J. & Chinsamy, A. Bone histology and growth patterns of some nonmammlian therapsids. *J Vertebr Paleontol* **24**, 634–648 (2004).

25. Ray, S., Mukherjee, D. & Bandyopadhyay, S. Growth patterns of fossil vertebrates as deduced from bone microstructure: case studies from India. *J Biosci* **34**, 661–672 (2009)

26. Chinsamy, A., & Hurum, J. H. Bone microstructure and growth patterns of early mammals. *Acta Palaeontol Pol* **51**, 325–338 (2006).

27. Botha-Brink, J., Bento-Soares, M. & Martinelli, A. Osteohistology of late Triassic prozostrodontian cynodonts from Brazil. *PeerJ* **6**, e5029 (2018)

28. Enlow, D.H. & Brown, S.O. Comparative histological study of fossil and recent bone tissues. Part III. *Texas J. Sci.* **10**, 187–230 (1958).

29. Buffrénil, V., Muizon, C., Dumont, M., Laurin, M. & Lambert, O. In *Vertebrate skeletal histology and paleohistology* (Buffrénil, V., Ricqlès, A.J., Zylberberg, L. & Padian, K., eds.) 564–616 (Boca Raton and London, CRC Press, 2021).

30. Hurum, J. H., & Chinsamy-Turan, A. In *Forerunners of mammals: radiation, histology, biology* (Chinsamy-Turan, A, ed) 248-270 (Indiana University Press, Bloomington, 2012).

31. Köhler, M., Marín-Moratalla, N., Jordana, X. & Aanes, R. Seasonal bone growth and physiology in endotherms shed light on dinosaur physiology. *Nature* **487**, 358–361 (2012).

32. Csiki-Sava, Z., Vremir, M., Meng, J., Brusatte, S. L. & Norell, M. A. Dome-headed, small-brained island mammal from the Late Cretaceous of Romania. *PNAS* **115**, 4857-4862 (2018).

33. Chen, M., Strömberg, C. A. & Wilson, G. P. Assembly of modern mammal community structure driven by Late Cretaceous dental evolution, rise of flowering plants, and dinosaur demise. *PNAS* **116**, 9931-9940 (2019)

34. Krause, D. W. et al. Skeleton of a Cretaceous mammal from Madagascar reflects long-term insularity. *Nature* **581**, 421-427 (2020).

35. Gurovich, Y. Additional specimens of sudamericid (Gondwanatheria) mammals from the early Paleocene of Argentina. *Palaeontology* **51**, 1069–1089 (2008).

36. Gingerich, P.D., Smith, B.H. & Rosenberg, K. Allometric scaling in the dentition of primates and prediction of body weight from tooth size in fossils. *Am J Phys Anthropol* **58**, 81–100 (1982).

37. Legendre, S. Les communautés de mammifères du Paléogène (Eocène supérieur et Oligocène) d'Europe occidentale: structures, milieux et évolution. *Münchner Geowiss Abh, A, Geol Paläontol* **16**, 1–110 (1989).

38. Bloch, J.I., Rose, K.D. & Gingerich, P.D. New species of *Batodonoides* (Lipotyphla, Geolabididae) from the Early Eocene of Wyoming: Smallest known mammal? *J Mammal* **79**, 804–827 (1998).

39. Gordon, C.L. A first look at estimating body size in dentally conservative marsupials. *J Mammal Evol* **10**, 1064–7554 (2003).

40. Wilson, G.P. et al. Adaptive radiation of multituberculate mammals before the extinction of dinosaurs. *Nature* **483**, 457–460 (2012).

41. Slater, G. J. Phylogenetic evidence for a shift in the mode of mammalian body size evolution at the Cretaceous-Palaeogene boundary. *Methods Ecol Evol* **4**, 734-744 (2013).

42. Rougier, G. W., Martinelli, A. G. & Forasiepi, A. M. *Mesozoic mammals from South America and their forerunners*. Springer Nature (2021).

43. Bonaparte, J.F. A new and unusual Late Cretaceous mammal from Patagonia. *J Vertebr Paleontol* **6**, 264–270 (1986).

44. Bonaparte, J.F. New Late Cretaceous mammals from the Los Alamitos Formation, northern Patagonia. *Natl Geogr Res* **6**, 63–93 (1990).

45. Chornogubsky, L. New remains of the dryolestoid mammal *Leonardus cuspidatus* from the Los Alamitos Formation (Late Cretaceous, Argentina). *Paläontol Z* **85**, 343–350 (2011).

46. Bonaparte, J.F. Sobre *Mesungulatum houssayi* y nuevos mamíferos cretácicos de Patagonia. *4° Congr Argentino Paleontol Bioestr* **2**, 48–61 (1986).

47. Martinelli, A. G. et al. New cladotherian mammal from southern Chile and the evolution of mesungulatid meridiolestidans at the dusk of the Mesozoic era. *Sci Rep* **11**, 7594 (2021).

48. Harper, T., Parras, A., & Rougier, G. W. *Reigitherium* (Meridiolestida, Mesungulatoidea) an enigmatic Late Cretaceous mammal from Patagonia, Argentina: morphology, affinities, and dental evolution. *J Mammal Evol* **26**, 447-478 (2019).

49. Rougier, G. W. et al. New Specimens of *Reigitherium bunodontum* from the Late Cretaceous La Colonia Formation, Patagonia, Argentina and Meridiolestidan Diversity in South America. *J Mammal Evol* **28**, 1051-1081 (2021).

50. Bonaparte, J.F. New Dryolestida (Theria) from the Late Cretaceous of Los Alamitos, Argentina, and paleogeographical comments. *N Jb Geol Paläontol Abh* **224**, 339-371 (2002)

51. Rougier, G.W., Chornogubsky, L., Casadio, S., Paéz Arango, N. & Giallombardo, A. Mammals from the Allen Formation, Late Cretaceous, Argentina. *Cretaceous Res* **30**, 223–238 (2009).

52. Scillato-Yané, G.J. & Pascual, R. Un peculiar Xenarthra del Paleoceno Medio de Patagonia (Argentina). Su importancia en la sistemática de los Paratheria. *Ameghiniana* **21**, 316–318 (1985).

53. Goin, F. J. et al. First Mesozoic mammal from Chile: The southermost record of a Late Cretaceous gondwanatherian. *Bol Mus Nac Hist Nat Chile* **69**, 5–31 (2020).

54. Chimento, N. R., Agnolin, F. L., Tsuihiji, T., Manabe, M., & Novas, F. E. New record of a Mesozoic gondwanatherian mammaliaform from Southern Patagonia. *Sc Nat* **107**, 1-7 (2020).

55. Chimento, N. R. et al. First monotreme from the Late Cretaceous of South America. *Commun Biol* **6**, 146 (2023).

56. Woodburne, M. O. & Tedford, R. H. The first Tertiary monotreme from Australia. *Am Mus Nov* **2588**, 1–11 (1975).

57. Flannery, T. F. et al. A review of monotreme (Monotremata) evolution. *Alcheringa* **46**, 3-20 (2022).

58. Janis, C.M. In *Body Size in Mammalian Paleobiology* (Damuth, J. & MacFadden, B.J. eds.) 255–300 (Cambridge University Press, Cambridge, 1990).

59. Luo, Z.X., Crompton, A.W. & Sun, A.L. A new Mammaliaform from the Early Jurassic and evolution of mammalian characteristics. *Science* **292**, 1535–1540 (2001).

60. Foster, J.R. Preliminary mass estimates for mammalian genera of the Morrison formation (Upper Jurassic; North America). *PaleoBios* **28**, 114–122 (2009).

61. Kirk, E. C., Hoffmann, S., Kemp, A. D., Krause, D. W., & O'Connor, P. M. Sensory anatomy and sensory ecology of *Vintana sertichi* (Mammalia, Gondwanatheria) from the Late Cretaceous of Madagascar. *J Vert Paleontol* **34(sup1)**, 203-222 (2014).

62. Martin, T. et al. A Cretaceous eutriconodont and integument evolution in early mammals. *Nature* **526**, 380-384 (2015).

63. Krause, D. W. et al. First cranial remains of a gondwanatherian mammal reveal remarkable mosaicism. *Nature* **515**, 512–517 (2014).

64. Rougier, G. W., Forasiepi, A. M., Hill, R. V. & Novacek, M. J. New mammalian remains fromthe Late Cretaceous La Colonia Formation, Patagonia, Argentina. *Acta Palaeontol Pol* **54**, 195–212 (2009).

65. Rougier, G. W., Apesteguía, S. & Gaetano, L. C. Highly specialized mammalian skulls from the Late Cretaceous of South America. *Nature* **479**, 98-102 (2011).

66. Forasiepi, A. M., Coria, R. A., Hurum, J., & Currie, P. J. First Dryolestoid (Mammalia, Dryolestoidea, Meridiolestida) from the Coniacian of Patagonia and new evidence on their early radiation in South America. *Ameghiniana* **49**, 497-504 (2012).

67. Hu, Y., Meng, J., Wang, Y. & Li, C. Large Mesozoic mammals fed on young dinosaurs. *Nature* **433**, 149-152 (2005).

68. Rougier, G. W., Novacek, M. J., Pascual, R., Gelfo, J. N. & Cladera, D. New Late Cretaceous mammals from Argentina and the survival of Mesozoic lineages in the Patagonian Early Tertiary. *J Vert Paleontol* **20(3, Suppl)**, 65A (2000).

69. Krause, D. W., Groenke, J. R., Hoffmann, S., Rogers, R. R., & Rahantarisoa, L. J. Introduction to *Adalatherium hui* (Gondwanatheria, Mammalia) from the Late Cretaceous of Madagascar. *J Vert Paleontol* **40(sup1)**, 4-18 (2020).

70. Thewissen, J. G. M., & Gingerich, P. D. Skull and endocranial cast of *Eoryctes melanus*, a new palaeoryctid (Mammalia: Insectivora) from the early Eocene of western North America. *J Vert Paleontol* **9**, 459-470 (1989).

71. Van Valkenburgh, B. In *Body Size in Mammalian Paleobiology* (Damuth, J. & MacFadden, B.J. eds.) 181–205 (Cambridge University Press, Cambridge, 1990).

72. Millien, V., & Bovy, H. When teeth and bones disagree: body mass estimation of a giant extinct rodent. *J Mammal* **91**, 11-18 (2010).

73. Anderson, J. F., Hall-Martin, A. & D. A. Russell. Long-bone circumference and weight in mammals, birds and dinosaurs. *J Zool Soc London A* **207**, 53–61 (1985).

74. Christiansen, P. Scaling of the limb long bones to body mass in terrestrial mammals. *J Morphol* **239**, 167–190 (1999).

75. Christiansen, P. & Harris, J. M. Body size of *Smilodon* (Mammalia: Felidae). *J Morphol* **266**, 369–384 (2005).

76. Figueirido, B., Pérez−Claros, J.A., Hunt, R.M. Jr & Palmqvist, P. Body mass estimation in amphicyonid carnivoran mammals: A multiple regression approach from the skull and skeleton. *Acta Palaeontol Pol* **56**, 225–246 (2011).

77. Campione, N. E. & D. C. Evans. A universal scaling relationship between body mass and proximal limb bone dimensions in quadrupedal terrestrial tetrapods. *BMC Biol* **10**, 60 (2012).

78. Campione, N. E. Extrapolating body masses in large terrestrial vertebrates. *Paleobiology* **43**, 693-699 (2017).

79. Basú, C., Falkingham, P.L. & Hutchinson, J.R. The extinct, giant giraffid *Sivatherium giganteum*: skeletal reconstruction and body mass estimation. *Biol Lett* **12**, 20150940 (2016).

80. Young, C. B. Static allometry of a small-bodied omnivore: body size and limb scaling of an island fox and inferences for Homo floresiensis. *J Human Evol* **149**, 102899 (2020).

81. Romano, M., Manucci, F. & Palombo, M. R. The smallest of the largest: new volumetric body mass estimate and in-vivo restoration of the dwarf elephant

Palaeoloxodon ex gr. *P. falconeri* from Spinagallo Cave (Sicily). *Hist Biol* **33**, 340-353 (2021).

82. Dantas, M. A. Estimating the body mass of the late Pleistocene megafauna from the South America Intertropical Region and a new regression to estimate the body mass of extinct xenarthrans. *J South Am Earth Sci* **119**, 103900 (2022).

83. Legendre, S. Analysis of mammalian communities from the late Eocene and Oligocene of southern France. *Palaeovertebrata* **16**, 191-212 (1986).

84. Lyson, T.R. et al. Exceptional continental record of biotic recovery after the Cretaceous–Paleogene mass extinction. *Science* **366**, 977-983 (2019).

85. Bellani, G. G. *Felines of the world: discoveries in taxonomic classification and history*. Academic Press (2019).

86. Grano, M. An unusual urban refuge for the crested porcupine, *Hystrix cristata* (Linnaeus, 1758) (Mammalia Rodentia): the ancient Catacombs of Priscilla in Rome (Italy). *Biodiv J* **7**, 345-346 (2016).

87. Jiménez, J. E., Yáñez, J. L., Tabilo, E. L., & Jaksić, F. M. Body size of Chilean foxes: a new pattern in light of new data. *Acta Theriol* **40**, 321-326 (1995).

88. Novaro, A. J. *Pseudalopex culpaeus*. *Mamm Species* **558**, 1-8 (1997).

89. Cravino, J. L. et al. Análisis holístico de la predación en corderos: un estudio de caso, con énfasis en la acción de "Zorros" (Mammalia: Canidae). *Vet (Montevideo)*, **35**, 24-42 (2000).

90. Zapata, S. C., Travaini, A., Delibes, M., & Martínez-Peck, R. Food habits and resource partitioning between grey and culpeo foxes in southeastern Argentine Patagonia. *Stud Neotrop Fauna Environ* **40**, 97-103 (2005).

91. Soler, L., González, M. J. P., & Casanave, E. B. In *El Aguará Guazú en la Argentina* (Orozco, M., González Ciccia, P. & Soler, L., eds.) 43-57 (Vazquez Mazzini Editores, Buenos Aires, 2015).

92. Lambert, J. E., Fellner, V., McKenney, E., & Hartstone-Rose, A. Binturong (*Arctictis binturong*) and kinkajou (*Potos flavus*) digestive strategy: implications for interpreting frugivory in Carnivora and Primates. *PLoS One* **9**, e105415 (2014).

93. Long, J. L. *Introduced mammals of the world: their history, distribution and influence*. CSIRO publishing (2003).

94. Lavirière, S. *Lontra longicaudis*. *Mamm Species* **609**, 1-5 (1999).

95. Gompper, M. E. & Decker, D. M. *Nasua nasua*. *Mamm Species* **580**, 1-9 (1998).

96. Robinson, J.G. & Redford, K.H. Body size, diet, and population density of Neotropical forest mammals. *Am Nat* **128**, 665-680 (1986).

97. Pautasso, A. A., Fandiño, B., & Raimondi, V. B. Mamíferos de la provincia de Santa Fe, Argentina. *Com Mus Provincial Cienc Nat "Florentino Ameghino" (NS)* **13**, 1-248 (2008).

98. Koehler, C. E. & Richardson, P. R. K. (1990). *Proteles cristatus*. *Mamm Species* **363**, 1-6 (1990).

99. Larivière, S. *Lontra provocax. Mamm Species* **610**, 1-4 (1999).

100. Belant, J. L. Field immobilization of raccoons (*Procyon lotor*) with telazol and xylazine. *J Wildl Dis* **40**, 787-790 (2004).

101. Nowak, R. M. *Walker's Mammals of the word. Fifth edition*. Johns Hopkins University Press (1991).

102. Jackson, J. E., Branch, L. C., & Villarreal, D. *Lagostomus maximus*. *Mamm Species* **543**, 1-6 (1996).

103. Courtenay, O. & Maffei, L. In *Canids: Foxes, Wolves, Jackals and Dogs-Status Survey and Conservation Action Plan* (C. Sillero-Zubiri, M. Hoffmann & DW Macdonald, eds) 32-38 (IUCN/SSC Canid Specialist Group, Cambridge, 2004).

104. Weber, M. & Gonzalez, S. Latin American deer diversity and conservation: a review of status and distribution. *Ecoscience* **10**, 443-454 (2003).

105. Fooden, J. & Aimi, M. Systematic review of Japanese macaques, *Macaca fuscata* (Gray, 1870). *Fieldiana Zool* **104**, 1-198 (2005).

106. Fedigan, L. M. *Primate paradigms: Sex roles and social bonds*. The University of Chicago Press (1992).

107. Nowak, R. M. *Walker's mammals of the world: monotremes, marsupials, afrotherians, xenarthrans, and sundatherians*. Johns Hopkins University Press, (2018).

108. Eisenberg, J. F. & Redford, K. H. *Mammals of the Neotropics, Volume 3: The Central Neotropics: Ecuador, Peru, Bolivia, Brazil.* The University of Chicago Press, Chicago (1999).

109. Guichón, M.L., Doncaster, C.P. & Cassini, M.H. Population structure of coypus (*Myocastor coypus*) in their region of origin and comparison with introduced populations. *J Zool* **261**, 265-272 (2003).

110. Zhou, C. F., Bhullar, B. A. S., Neander, A. I., Martin, T., & Luo, Z. X. New Jurassic mammaliaform sheds light on early evolution of mammal-like hyoid bones. *Science* **365**, 276-279 (2019).

111. Goloboff, P. A., Farris, J. S. & Nixon, K. C. TNT, a free program for phylogenetic analysis. *Cladistics* **24**, 774–786 (2008).

112. Goloboff, P. A. & Catalano, S. A. TNT version 1.5, including a full implementation of phylogenetic morphometrics. *Cladistics* **32**, 221–238 (2016).

113. Swofford, D. L. & Begle, D. P. PAUP User's Manual. Center for Biodiversity, Illinois Natural History Survey, Champaign, Illinois (1993).

114. Coddington, J. & Scharff, N. Problems with zero-length branches. *Cladistics* **10**, 415–423 (1994).

115. Bremer, K. The limits of amino acid sequence data in angiosperm phylogenetic reconstruction. *Evolution* **42**, 795–803 (1988).

116. Bremer, K.R. Branch support and tree stability. *Cladistics* **10**, 295–304 (1994).

117. Felsenstein, J. Confidence limits on phylogenies: an approach using the bootstrap. *Evolution* **39**, 783–791 (1985).

118. Goloboff, P., Farris, J. & Nixon, K. T.N.T: Tree Analysis Using New Technology. program available from the authors and www.zmuc.dk/public/phylogeny/tnt (2003).

119. Luo, Z.-X., Gatesy, S. M., Jenkins Jr., F. A., Amaral, W. W., Shubin, N. H. Mandibular and dental characteristics of Late Triassic mammaliaform *Haramiyavia* and their ramifications for basal mammal evolution. *PNAS* **112**, E7101–E7109 (2015).

120. Huttenlocker, A. K., Grossnickle, D. M., Kirkland, J. I., Schultz, J. A., Luo, Z.-X. Late surviving stem mam mal links the lowermost Cretaceous of North America and Gondwana. *Nature* **558**, 108–112 (2018).
